# Early Gravettian genomic data from Western Europe reveal population movements before the LGM

**DOI:** 10.1126/sciadv.aeh6128

**Published:** 2026-10-23

**Authors:** Pere Gelabert, Susanna Sawyer, Vanessa Villalba-Mouco, Olivia Cheronet, Victoria Oberreiter, Diego Gárate, Christian Normand, Lawrence G. Straus, Manuel R. González Morales, Igor Gutiérrez-Zugasti, David Cuenca-Solana, Joëlle Darricau, Henry de Lumley, José-Miguel Tejero, Mareike Stahlschmidt, Thomas Higham, Maddalena Giannì, Laura G. van der Sluis, Carla Gómez-Montes, Miriam Ibáñez-Herranz, Michelle Hämmerle, Brina Zagorc, Florian Exler, Florian Brück, Stefan Franz, Fernanda Tenorio Cano, Kristin Stewardson, Lijun Qiu, Ana B. Marín-Arroyo, Michaela Lucci, Alessia Nava, Francesco Genchi, Donato Coppola, Chiara La Marca, Alfredo Coppa, David Reich, Ron Pinhasi

**Affiliations:** ^1^ Department of Evolutionary Anthropology, University of Vienna, Vienna, Austria.; ^2^ Human Evolution and Archaeological Sciences (HEAS), University of Vienna, Vienna, Austria.; ^3^ Institute of Evolutionary Biology, CSIC-Universitat Pompeu Fabra, Barcelona, Spain.; ^4^ University of Veterinary Medicine, Konrad Lorenz Institute of Ethology, Savoyenstraße 1, 1160 Vienna, Austria.; ^5^ Instituto Internacional de Investigaciones Prehistóricas de Cantabria (Universidad de Cantabria, Gobierno de Cantabria, Banco Santander), Santander, Spain.; ^6^ Univ. Toulouse Jean Jaurès, TRACES UMR 5608, Maison de la Recherche, 5, Allée Antonio Machado, 31058 Toulouse cedex 9, France.; ^7^ Association Eusko Arkeologia, Jauregia, 64120 Larceveau-Arros-Cibits, France.; ^8^ Department of Anthropology, University of New Mexico, Albuquerque, NM, USA.; ^9^ Grupo I+D+i EvoAdapta, Departamento de Ciencias Históricas, Universidad de Cantabria, Santander, Spain.; ^10^ Centre de Recherche en Archéologie, Archéosciences, Histoire (CReAAH), UMR 6566, Rennes, France.; ^11^ Association Gaztelu, Oxocelhaya Borda, F-64640 Saint-Martin-d’Arberoue, France.; ^12^ Institut de Paléontologie Humaine (IPH), Fondation Albert-1Er Prince de Monaco, Paris, France.; ^13^ Centre Européen de Recherches Préhistoriques de Tautavel (CERPT), Tautavel, France.; ^14^ Seminari d’Estudis i Recerques Prehistòriques (SERP), University of Barcelona, Barcelona, Spain.; ^15^ Institute of Anthropological and Spatial Studies, Research Centre of the Slovenian Academy of the Sciences and Arts, Ljubljana, Slovenia.; ^16^ Department of Human Evolutionary Biology, Harvard University, Cambridge, MA, USA.; ^17^ Department of Genetics, Harvard Medical School, Boston, MA, USA.; ^18^ Department of Environmental Biology, Sapienza University of Rome, Rome, Italy.; ^19^ Department of Odontostomatological and Maxillofacial Sciences, Sapienza University of Rome, Rome, Italy.; ^20^ Department of Oriental Studies, Sapienza University of Rome, Rome, Italy.; ^21^ Museo di Civiltà preclassiche della Murgia meridionale, Ostuni 72017, Italy.; ^22^ Soprintendenza Archeologia Belle Arti e Paesaggio per le Province di Brindisi, Lecce e Taranto, Italy.; ^23^ Department of History, Anthropology, Religions, Art, Performing Arts, Sapienza University of Rome, Rome, Italy.; ^24^ Unitelma Sapienza, Rome, Italy.; ^25^ Howard Hughes Medical Institute, Boston, MA, USA.; ^26^ Broad Institute of MIT and Harvard, Cambridge, MA, USA.; ^27^ The Second Hospital of Jilin University, Jilin University, Changchun, Jilin Province 130041, China.; ^28^ Joint International Research Laboratory of Ageing Active Strategy and Bionic Health in Northeast Asia of Ministry of Education, Jilin University, Changchun, Jilin Province 130041, China.

## Abstract

Anatomically modern humans first settled in Europe at least 45,000 years ago, yet limited genomic data from 45 to 20 thousand years (ka) constrain reconstructions of Upper Palaeolithic population dynamics. Before the Last Glacial Maximum (LGM; ∼26.5 to 19 ka), Gravettian groups (∼34 to 25 ka) were widespread across Europe and genetically diverse, including the Fournol and Věstonice lineages. Here, we present genome-wide data from three Gravettian individuals: two from the Franco-Cantabrian region (Chufín, Spain, and Isturitz, France) and one from Italy (Ostuni1b). Our analyses reveal previously undetected gene flow between eastern and western early Gravettian populations in southwestern Europe, challenging models of regional genetic continuity from the Aurignacian to the Solutrean (∼24 to 19 ka). To investigate population history during the LGM, a period for which human skeletal remains are extremely rare, we analyzed sedimentary ancient DNA from Solutrean Layer 122 at El Mirón Cave (Cantabria, Spain; ∼22,000 calibrated years before the present), recovering ∼14,000 human single-nucleotide polymorphisms. These data support persistence of Fournol-related ancestry in southwestern Europe throughout the LGM.

## INTRODUCTION

The Gravettian [∼34 to 25 thousand years (ka)] was a distinctive and widespread archaeological culture in Europe that spanned the period preceding and the onset of the Last Glacial Maximum (LGM) (*1*, *2*). Despite broad similarities in lithic and osseous technologies, burial practices, and portable artworks, the Gravettian was not entirely homogeneous. Analyses of material culture have suggested the existence of several distinct regional groups during this period (*3*). At the same time, the study of portable art (*3*), lithic tools (*2*), and bone tools (*4*) as well as the morphological study of Gravettian human remains (*5*, *6*) collectively point to substantial interconnectivity across the continent, implying the existence of large-scale, pan-European social networks.

Unexpectedly, this pattern of widespread cultural interaction is not mirrored in the genetic background of these populations. Ancient genomic data reveal at least two distinct ancestry clusters that remained relatively stable throughout the Gravettian cultural horizon in Europe. The first is a western and southwestern (SW) ancestry known as the Fournol cluster, which derived from the ∼35-ka individual Goyet Q116-1 (Aurignacian, Belgium) (*7*, *8*). The second is the Věstonice cluster, primarily represented by genomes from Dolní Věstonice (Czechia) alongside other Central European and Italian individuals (*7*, *8*). This ancestry likely resulted from an earlier admixture event between a lineage represented by the individuals Kostenki14 and Sungir (37 to 34 ka) (*9*, *10*) and a population that was both related to the Aurignacian-related Goyet Q116-1 and the probably-Aurignacian Bacho Kiro BK-1653 individual (*8*, *11*, *12*). The timing and geographic context of these admixture events, as well as their relationship to cultural processes or climatic changes, remain poorly understood due to the limited number of specimens that have yielded genome-wide data. Published Gravettian genomes from central and western Europe remain sparse, including individuals from Dolní Věstonice and Pavlov (Czech Republic); Krems-Wachtberg (Austria); Paglicci and Ostuni (Italy); Goyet (Belgium); Mollet III and Reclau Viver (Spain); and La Rochette, Fournol, and Ormesson (France) (fig. S1) (*7*, *8*, *13*). Consequently, the current dataset is insufficient to fully assess the degree of genetic isolation between these clusters, their temporal dynamics, or the demographic shifts preceding the emergence of the Solutrean in SW Europe (∼24.5 to 20 ka) and the Epigravettian in Italy and southeastern Europe (∼24 to 12 ka).

Despite this apparent genetic structure during the whole Gravettian culture, evidence for interaction between these ancestry groups is not entirely absent. The clearest documented instance involves Late Gravettian individuals from Goyet Cave, Belgium (∼27 ka), who have mixed ancestry derived from both the Věstonice and Fournol clusters. This admixture suggests an east-to-west expansion of Věstonice-related ancestry between the Early/Middle and Late Gravettian, establishing a longitudinal cline across pre-LGM European populations (*8*). It is relevant to mention that the Goyet cave is thought to be at the western geographical boundary of the Věstonice ancestry. Similar signals of interaction have yet to be documented in Southern Europe. Within this framework, the Franco-Cantabrian and Italian regions are of particular interest; they later served as major LGM refugia and preserve some of the richest Upper Palaeolithic records in Europe, a pattern shaped by both a long-standing regional research tradition and a karstic landscape that has provided numerous cave sites occupied since early prehistory (*14*–*17*). However, our ability to interpret these regions is often constrained by the scarcity of skeletal remains suitable for investigating population continuity or admixture.

In the absence of fossils, sedimentary DNA (sedaDNA) provides an alternative means of linking archaeological assemblages to human populations (*18*, *19*). Moreover, harnessing sedaDNA from successive deposits can enable fine-scaled diachronic analyses [but see (*20*, *21*)], which are difficult to achieve through palaeogenomic studies of Palaeolithic human remains given their scarcity (*22*, *23*). Given that archaeological cultures like the Gravettian, Epigravettian, and Solutrean persisted for millennia yet now rely on genome-wide data from fewer than 50 individuals, this approach is essential for resolving long-standing questions regarding human demographic history (*7*, *8*, *13*). Previous work at El Mirón Cave (Cantabria, Spain) has demonstrated the preservation of human mitochondrial DNA (mtDNA) in Palaeolithic sediments (*22*), motivating the effort to recover autosomal data from Solutrean deposits. While sedaDNA provides a powerful means of accessing otherwise inaccessible genetic information, it also presents substantial challenges related to DNA degradation, contamination, and the mixing of genetic material from multiple individuals.

In this study, we aim to refine current models of Gravettian population structure and assess the extent and nature of long-distance genetic interactions in pre-LGM Europe. We bridge these gaps by reporting new genome-wide data from three Gravettian skeletal individuals alongside human sequencing data retrieved from Solutrean sediments, coanalyzed with the already published data. The newly sequenced skeletal remains include an individual from Isturitz Cave [Basque Country, SW France, 26,030 ± 220 before the present (B.P.), OxAX-2616-50], a site long recognized for its exceptionally rich and long archaeological record and its potential role as a regional aggregation locality (*24*–*26*); an individual from Chufín Cave (*14*, *27*) (Cantabria, Spain, 22,793 ± 116 B.P., VIE-1682), representing a later Gravettian presence in Cantabria; and an individual from Ostuni Cave in Southern Italy (*28*) (Ostuni1b), which provides insight into population structure within the South-Central European Gravettian. In addition, we generated human sequencing data from Solutrean sediments at El Mirón Cave (*22*).

## RESULTS

### Genome-wide data from Gravettian-related skeletal human remains

We generated genome-wide data from three Gravettian individuals from SW and Southern Europe: Isturitz, Chufín, and Ostuni1b (Fig. 1, A and C; section S1 to S3; and tables S1 and S2). The Isturitz genome, now the oldest genome-wide dataset from a modern human in SW Europe (Fig. 1B), was produced using TWIST ancient DNA capture (table S1). For Chufín and Ostuni1b, we combined TWIST capture with shotgun sequencing (tables S1 and S2). In total, we prepared four libraries from two extracts of a Chufín tooth, eight libraries from two extracts of an Isturitz bone fragment, and two libraries from an Ostuni1b bone sample extract. Previous studies have shown that combining capture and shotgun data has only a limited impact on allelic bias in population genetic analyses (*29*).

**Fig. 1.**
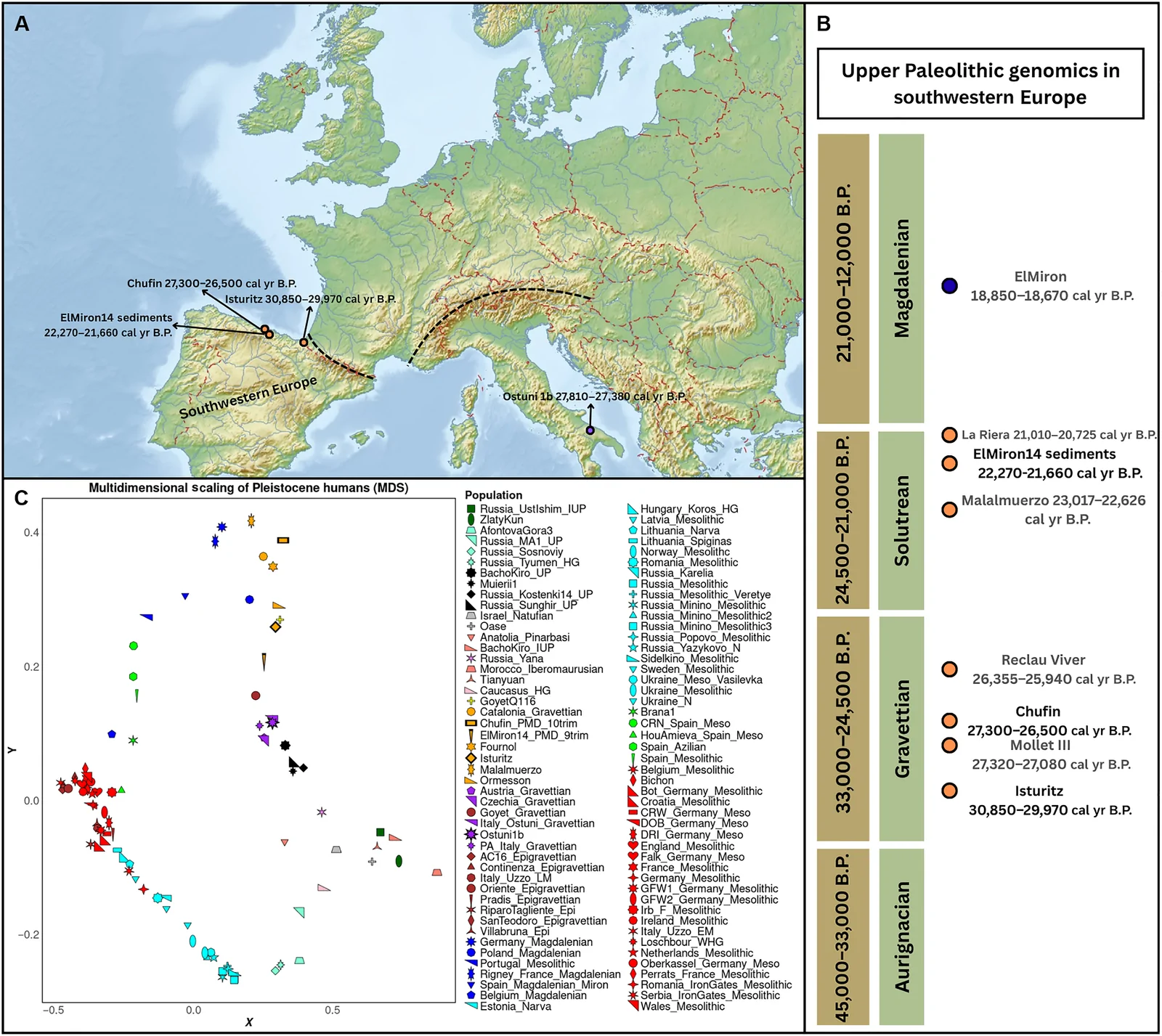
Genomic data from four new individuals from Gravettian and Solutrean cultures. (**A**) Location of the sampled individuals and estimated dates. The map was obtained from https://mapswire.com under a CC BY 4.0 license (https://creativecommons.org/licenses/by/4.0/). Dotted lines represent mountainous regions. (**B**) Published human genomic data from SW Europe. The bold samples are the newly reported in this study. The vertical axis depicts chronologies and cultures of the SW Europe Palaeolithic. (**C**) Multidimensional scaling (MDS) plot of ancient human data from the Late Pleistocene. ElMiron14_PMD_9trim, Chufin_PMD_10trim, and Isturitz are associated with the Fournol cluster individuals; Ostuni1b is associated with the Vêstonice cluster and is closely related to its mother, labeled Ostuni.

We recovered 454,258 single-nucleotide polymorphisms (SNPs) from the Gravettian adult female from Isturitz and 467,368 SNPs from the male fetus Ostuni1b, both with estimated contamination levels below 5% (table S1). These two samples are among the Gravettian individuals with the highest SNP counts now available. Using READ v2 (*30*), we identified Ostuni1b as a first-degree relative of the previously published Ostuni 1 female (fig. S8), most likely her son (section S3). This means that the date of Ostuni1b is approximately dated to the same age, 27,810 to 27,430 calibrated years before the present (cal yr B.P.) (*7*). For the Chufín adult female tooth, we detected mitochondrial contamination from modern human DNA ranging between 5 and 10% across libraries (table S2). To minimize its impact, we restricted SNP calling to damaged molecules in the Chufín dataset. In total, we recovered 19,895 SNPs overlapping the 1,233,013-site “1240k” panel for the resulting dataset (“Chufin_PMD_10trim”).

#### Low-coverage sedaDNA genome-wide data

To expand the Solutrean genomic data catalog, now only represented by three genetic samples (table S5), two of which (La Riera and La Piage) are of very low quality, we generated genome-wide data from an ElMiron14 sediment sample, the best preserved sample from our previous mtDNA analysis of sediments from this site (*22*). The sampled level is directly radiocarbon dated to 22,100 to 21,730 cal yr B.P., representing the final phases of the Solutrean occupation of the cave (*31*). To generate sufficient data for genome-wide analysis, we produced nine libraries from two extracts of ElMiron14 (*22*). Each library was indexed and sequenced an average of ten times (in some cases, following TWIST in-solution enrichment), generating a total of 1,028,866,146 sequences (table S3). This strategy minimizes duplication by using different fractions of each library in successive sequencing runs. Of the 101 individual sequencing runs, two-thirds (68) were performed after in-solution enrichment with the TWIST 1.4 M (*29*) human nuclear capture kit, and one-third (33) were shotgun sequenced (section S4). Using the combined shotgun and in-solution capture strategy, we recovered more than 294,109 unique human reads from the ElMiron14 sediment sample, covering 51,081 SNPs from the 1240k panel. After applying damage restriction and trimming read ends, 14,737 SNPs from ElMiron14 were retained for downstream analyses (“ElMiron14_PMD_9trim”), generating sufficient genomic data to directly link the archaeological package of the cave with a specific genomic ancestry in the absence of human remains. The similar coverage observed across the autosomes and the X chromosome suggests that most of the recovered DNA likely originated from more male individuals than females (section S4.3). In addition, the classification of the animal DNA found in the shotgun libraries resembles the previously reported diversity (table S27).

#### East-west gene flow in Gravettian genomes of Western Europe

We analyzed genome-wide data from the newly generated individuals together with published genomes from other Upper Palaeolithic Europeans to reconstruct the temporal dynamics of genetic ancestry in SW Europe, with a particular focus on the Franco-Cantabrian region (table S5). Before this study, the earliest genomic data from an individual geographically close to SW Europe came from Fournol Cave in SW France (29,020 to 28,450 cal yr B.P.). The genetic profile of this individual defined a population cluster interpreted as reflecting genetic continuity from Western European Aurignacian groups, represented by Goyet Q116-1 (35,630 to 34,720 cal yr B.P.), through to the peak of the LGM, when the Solutrean culture existed in southern France and Iberia (*15*, *32*–*35*). This phase is represented by the Solutrean individual from Malalmuerzo Cave in southern Spain (23,020 to 22,630 cal yr B.P.) (*13*). Subsequently, this ancestry merged with that of Epigravettian populations from the Italian Peninsula, giving rise to the Magdalenian-related ancestry observed in northern Iberia, exemplified by the “Red Lady” burial from El Mirón (18,850 to 18,800 cal yr B.P.) (*7*).

We determined the mtDNA haplogroups of all newly generated individuals, which fall within lineages typical of Upper Palaeolithic and early Holocene populations across Eurasia (*7*, *8*, *36*). The Chufín female belonged to mitochondrial U8 haplogroup, the same as BK-1653 from Bacho Kiro Cave (Initial Upper Palaeolithic, Bulgaria) (*11*). Within this cluster, haplogroup U8a is also observed among individuals belonging to the Goyet Q2 genetic cluster. Although U8a is a rare subclade of U8 in present-day Europe, it occurs at relatively high frequencies among present-day Basques (*37*). Chufín Cave in Cantabria is relatively close to the Basque Country. The individuals from Isturitz Cave and Ostuni1b both carry mitochondrial haplogroup M, the same haplogroup reported for Ostuni1b’s mother, Ostuni1 (*7*). Haplogroup M was relatively common in Europe before the LGM, including in individuals such as Goyet Q116-1 but became very rare among post-LGM populations (*8*, *38*). The ElMiron14 sediment sample from El Mirón Cave was previously reported to carry haplogroup U2′3′4′7′8′9, a lineage observed during the Solutrean with higher frequencies in Western Europe (*8*, *13*). The new sequencing data (section S4 and table S4) support the attribution, showing variation within haplogroup U consistent with the presence of multiple individuals.

The three new SW European samples cluster with the previously described Fournol-like individuals both in multidimensional scaling (MDS) and principal components analysis (PCA) (Fig. 1B and figs. S2 and S3). Using several combinations of *f*_4_(Mbuti,X;ClusterA,ClusterB) (where X is Isturitz, ElMiron14_PMD_9trim, or Chufin_PMD_10trim), we validated that Isturitz, ElMiron14_PMD_9trim, and Chufin_PMD_10trim share the higher genetic ancestry with Fournol and not with the Vêstonice cluster (tables S5 and S6). These results suggest that the Goyet Q116-1–related ancestry, the only reported Aurignacian-related genomic ancestry, was already present in the Franco-Cantabrian region before 30 ka and persisted until ∼21 ka, as is demonstrated by the sedaDNA from the late Solutrean level 122 of El Mirón (*31*) as well as the published data from La Riera cave (eastern Asturias, Spain: 21,010 to 20,725 cal yr B.P.) (*8*).

Next, we explored possible genetic diversity among the SW and Western European Upper Palaeolithic genomes using different combinations of *f*_4_ statistics (*39*). The results of the statistic *f*_4_(Mbuti,X;Isturitz,Fournol) (Fig. 2B and table S7) indicate that all studied Iberian individuals exhibit greater affinity to Fournol than to Isturitz, although Isturitz is geographically closer to Iberia. This pattern may reflect a temporal signal during the Gravettian, as the Isturitz sequence is about 2000 years older than Fournol. Applying the test *f*_4_(Mbuti,X;Isturitz,GoyetQ116-1) (Fig. 2A and table S7), we found that Věstonice-like individuals exhibit greater affinity to Isturitz than to Goyet Q116-1. In contrast, Solutrean individuals from Iberia show no significant affinity to Isturitz compared with Goyet Q116-1, although the values show more similarity to Goyet Q116-1 (Fig. 2A). We then used the statistic *f*_4_(Mbuti,Villabruna;GoyetQ116-1,X) to assess whether any of the newly generated samples show affinity to Epigravettian ancestry. As expected, none of the newly reported genomes has significant affinity to Villabruna (Fig. 2C and table S7). Last, using the statistic *f*_4_(Mbuti.DG,X;GoyetQ116-1,Czechia_Gravettian), we observed that while the newly generated samples generally show greater affinity to Goyet Q116-1, the Isturitz individual displays values similar to those of Magdalenian samples (Fig. 2D and table S7). This pattern suggests a greater number of shared alleles between Isturitz and Czechia_Gravettian than between other Gravettian samples from SW or Western Europe, which is shown by the test *f*_4_(Mbuti,Czechia_Gravettian;GoyetQ116-1,Isturitz) = 0.003, SE = 7.02 × 10^–4^, *Z* = 4.904. Together, the data raise the possibility that the Isturitz individual received gene flow from eastern or central European populations, which is uncommon in the rest of the SW European Palaeolithic individuals. To validate these signals, we computed several tests using Russia_Sungir_UP and Russia_Kostenki14_UP ancestries (fig. S4 and table S7). The symmetry statistic *f*_4_(Mbuti.DG,Russia_Sungir_UP;Fournol,Isturitz) = 0.002, SE = 7.52 × 10^–4^, *Z* = 3.516 supports gene-flow between Isturitz and Eastern populations (after 35 ka) when compared with Fournol. This gene flow is also observed in some Věstonice-like individuals, but not in any published individual from SW Europe (fig. S4D and table S7) However, the statistic *f*_4_(Mbuti.DG,Russia_Kostenki14_UP;Fournol,Isturitz) = 5.52 × 10^–4^, SE = 9.18 × 10^–4^, *Z* = 0.60, is nonsignificant (fig. S4B and table S7). These same tests are less significant when Goyet Q116-1 is placed instead of Fournol (fig. S4, A and C, and table S7). The genome from Ormesson (Paris Basin, Gravettian) also shows increased affinity to Russia_Kostenki14_UP, but without statistical significance over Goyet Q116-1 or Fournol (fig. S4, A and B, and table S7).

**Fig. 2.**
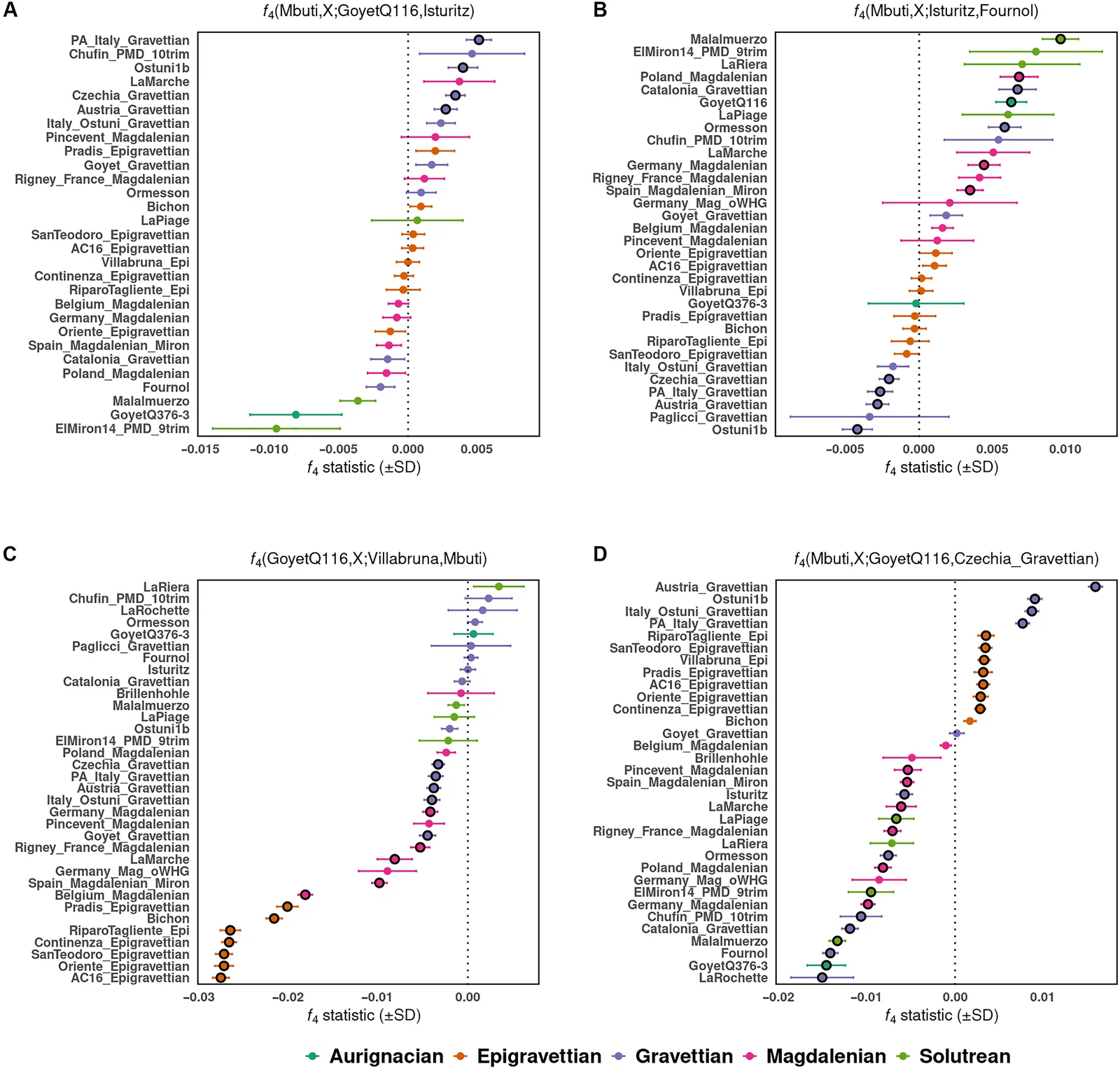
Genomic asymmetries of Isturitz compared to LGM populations of Western and Central Europe. Values represent *f*_4_ statistics. Non–black-outlined circles denote results that are not statistically significant (−3 < *Z* < 3). Colors indicate cultural attribution, and error bars represent one SE. (**A**) Gravettian-Věstonice–related populations share more derived alleles with Isturitz than with Goyet Q116-1. (**B**) Western European Gravettian and Solutrean individuals share more derived alleles with Fournol than with Isturitz. (**C**) The Aurignacian individual Goyet Q116-1 shows greater affinity to all pre-LGM individuals than to the Epigravettian Villabruna individual. (**D**) The Isturitz individual is the pre-LGM sample from the Franco-Cantabrian region showing the greatest affinity to Gravettian-Věstonice–related ancestry. Populations in all panels are sorted by *f*_4_-statistics values.

To explore models that could explain the previously described quantitative values of the *f* statistics relating individuals from SW Europe, we fitted admixture graphs using the find_graphs function in ADMIXTOOLS 2 (*40*). We used a combination of populations from Posth *et al.* (*8*), to which we added the Isturitz genome in 100 independent random searches, producing more than 100,000 possible trees (section S5). The best-fitting graph shows that the genome of Isturitz is consistent with deriving from an admixture of a dominant Fournol-like ancestry and an influx from a population most closely related to the ancestors of individuals from Věstonice (Fig. 3A) (*7*, *9*). Consistent with this model, the statistic *f*_4_(Mbuti.DG,Isturitz;Russia_Sungir_UP,Czechia_Gravettian) = 2.325 × 10^−3^ (SE = 5.33 × 10^−4^, *Z* = 4.360) shows that Isturitz shares greater affinity with Věstonice than with Russia_Sungir_UP. These genetic results could be explained by movements of people from Eastern Europe during the Gravettian period, which eventually contributed to this ancestry in Isturitz, or by a pre–Goyet Q116-1–like ancestry in the region, with some affinity to the Czechia_Gravettian cluster. The absence of any Aurignacian genome from the SW does not allow further interpretations. A group of Gravettian individuals from Goyet cave (NW Europe) has previously been reported as an intermediate between the Fournol and Věstonice clusters (*8*). Here, we show that these individuals are not significantly more closely related to Isturitz than to Fournol using *f*_4_(Mbuti.DG,Goyet_Gravettian;Isturitz,Fournol) = 0.0014, SE = 1.33 × 10^−3^, *Z* = 1.097.

**Fig. 3.**
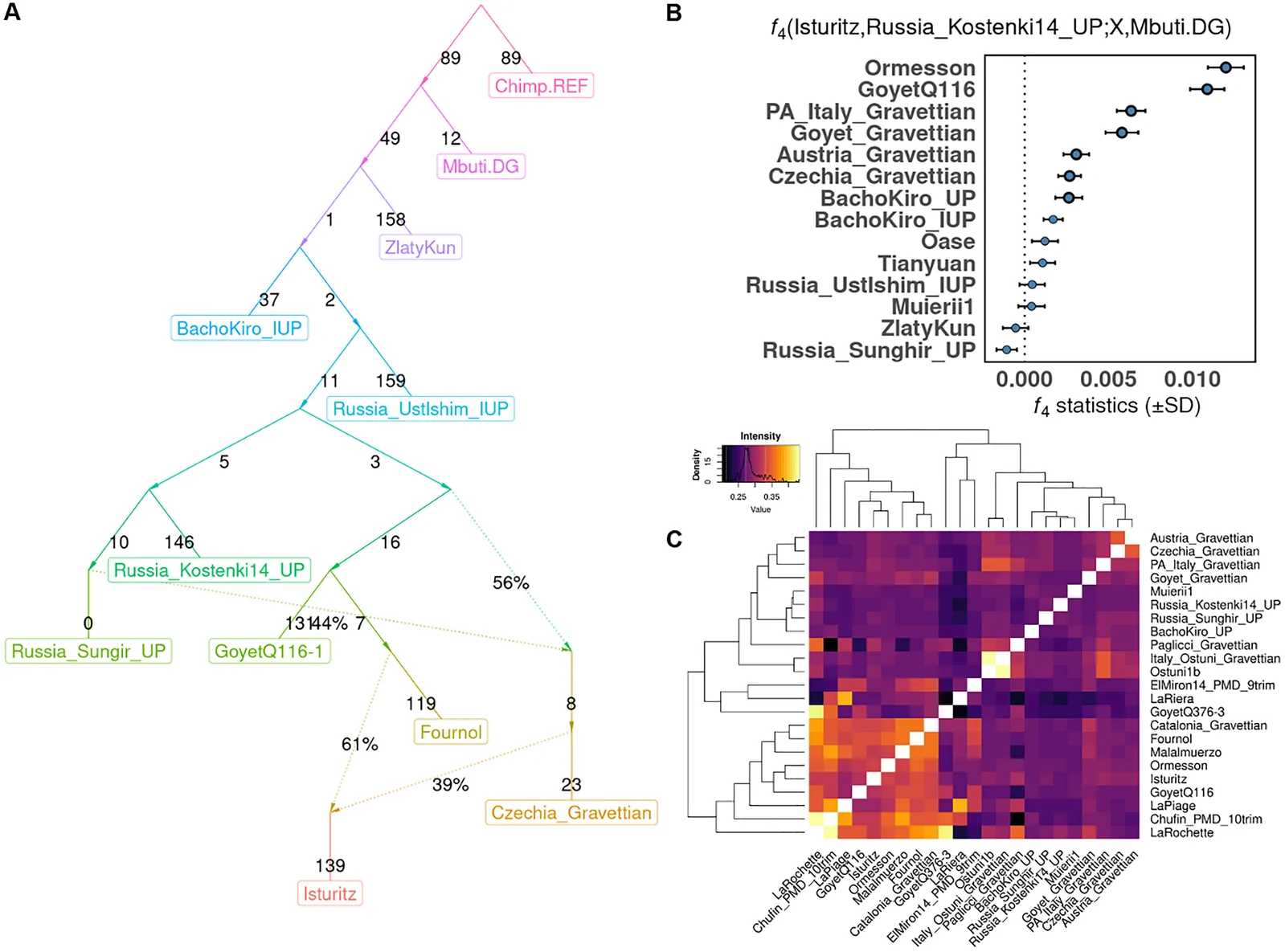
Genome-wide evidence that the Isturitz individual shows ancestry connections to Eastern Upper Palaeolithic populations. (**A**) Admixture graph 2 (the lowest score is 69.975) model showing the phylogenetic position of the Isturitz genome based on the framework of (*6*). In this model, Isturitz derives part of its ancestry from a lineage related to the ancestor of the Věstonice cluster, indicating gene flow from populations associated with Central/Eastern Europe. (**B**) Outgroup *f*_4_ statistics showing that Isturitz shares excess derived alleles with eastern European Upper Palaeolithic individuals (∼30 ka) relative to Kostenki14, including individuals from Bacho Kiro Cave. (**C**) *f*_3_ similarity matrix of Upper Palaeolithic European genomes. Isturitz shows the highest similarity to Ormesson, whereas ElMiron14_PMD_9trim shows affinity to contemporaneous individuals from the Cantabrian region such as La Riera and Chufín. Ostuni1b shows the highest affinity to Ostuni individuals.

To place these results in a broader context, we extended the analysis to Western European individuals associated with the Fournol cluster with >50,000 SNPs covering the 1240k panel (Malalmuerzo, Catalonia_Gravettian, and Ormesson; figs. S12 to S14). Unexpectedly, we also identified admixed ancestry in the genome of Ormesson (NW France, 31,822 to 29,970 cal yr B.P.) (section S5 and fig. S12), for which Kostenki14 was the best-fitting source, but not in the other tested individuals (figs. S13 and S14). This similar pattern was also shown in an *f*_3_ heatmap performed only with the Gravettian, Solutrean, and Aurignacian genomes from Europe, together with Russia_Kostenki14_UP and Russia_Sungir_UP (Fig. 3C). In this plot, the Isturitz individual appears to be the closest related to Ormesson. In the same analysis, ElMiron14_PMD_9trim shows the closest affinity to other Fournol-related individuals. We then used the statistic *f*_4_(Isturitz,Russia_Kostenki14_UP; X,Mbuti.DG), where X is a series of UP individuals older than 30,000 years from the Eurasian Upper Palaeolithic. Isturitz shows only shared derived alleles with Goyet Aurignacian and Gravettian individuals and Bacho Kiro UP individuals, compared with Russia_Kostenki14_14_UP (Fig. 3B). The result is consistent with the previously described results.

We estimated the timing of the identified gene-flow events using DATES_v4010. Our analysis yielded a statistically significant result only for the Isturitz model, which places the admixture event between 32,450 and 31,600 cal yr B.P. Although the date estimate for the Ormesson model did not reach statistical significance, its range largely overlaps with the Isturitz results (table S29). Together, these findings suggest a pulse of gene flow originating between 31,500 and 32,500 cal yr B.P., assuming that the identified gene flow corresponds to a single pulse.

### Low genomic homozygosity in Ostuni1b

We assigned Ostuni1b Y-chromosome haplogroup to the broader C lineage: C*(xC-M217, C-M8, C-V86, C-Y28069, C-B65, and C-M208). This haplogroup is common for pre-LGM individuals such as Vestonice16, Kostenki14, and Goyet Q116-1, showing the expected results (*7*). Using HIrisPlex-S (*41*), we determined that Ostuni1b showed phenotypic traits typical of the Upper Pleistocene in Europe, such as dark skin, dark hair, and brown eyes. These same traits were predicted for the other two newly reported individuals, Chufín and Isturitz, as well as in the sedaDNA data from ElMiron14 (section S5).

In the MDS plot, Ostuni1b clusters very closely with his mother, Ostuni1 (Fig. 1), as expected for first-degree relatives (see the Supplementary Materials). To investigate the ancestry of Ostuni1b’s father, we compared Ostuni1b and his mother using the statistic *f*_4_(Mbuti,X;Ostuni1,Ostuni1b). If the father had a substantially different ancestry from the mother, then the statistic would show a directional deviation. However, we observe no significant differences, indicating that the father and mother likely came from the same population within the limits of our resolution (fig. S5A). Furthermore, using the genetic data of Ostuni1b alongside other Věstonice cluster individuals (with >50,000 SNPs), we demonstrate that the Fournol-related individuals do not show significant affinity to any specific Věstonice-associated Gravettian group (section S5, table S7, and fig. S15), which rules out a clear candidate group for the inferred genetic pulse in Western Europe.

Using hapROH, we estimated that Ostuni1b carries ∼80 centimorgans (cM) of runs of homozygosity (RoHs) longer than 4 cM. No RoH segments longer than 8 cM were detected, suggesting no evidence of close parental relatedness (fig. S5B). This pattern closely mirrors the data observed in the Isturitz individual and stands significantly lower than that of many other Gravettian individuals, although Isturitz shows one fragment bigger than 20 cM. We then attempted to estimate the effective population sizes of Isturitz and Ostuni1b related populations (fig. S5 and table S8). These two individuals could be modeled as originating from populations with effective sizes of 235 and 160, respectively; these values are higher than any other Gravettian available individual.

## DISCUSSION

Our understanding of the genomic make-up of Gravettian populations and their movements across the cultural span remains based on only a few pre–35-ka genomes and around 25 Gravettian-associated individuals spanning nearly 10,000 years (*7*, *8*). Although the broad genetic structure of Gravettian populations is now relatively well characterized, regional population dynamics and patterns of mobility within the Gravettian remain poorly understood. Likewise, the timing and processes underlying the emergence of Solutrean- and Epigravettian-associated lineages, as well as their eventual contribution to Magdalenian-associated gene pools, remain unresolved. Here, we expand the available dataset by introducing genomic data from three new individuals. Among these are two individuals, Ostuni1b and Isturitz, who represent some of the highest-coverage Gravettian genomic datasets now available, each yielding more than 400,000 recovered SNPs. Notably, our analyses reveal that these individuals likely originated from populations with larger effective sizes than other Gravettian individuals, a pattern that may be driven by geographical diversity. In contrast, the heavily degraded Chufín tooth yielded substantially fewer data, preventing a detailed investigation of its ancestry.

Using multiple analytical approaches, we identify evidence of east-west gene-flow within Gravettian populations in Isturitz and Ormesson genomic data. Previous work has already documented mixed ancestry in individuals from Goyet Cave in Belgium (28 to 26 ka), who occupy an intermediate genetic position between the Věstonice and Fournol clusters (*8*). Here, we extend this pattern by showing that the genome of a female from Isturitz Cave (∼30 ka), which primarily belongs to the Fournol cluster, also exhibits detectable affinity to Věstonice-related ancestry. A comparable signal is observed in the previously published Gravettian individual from Ormesson. Notably, both the Isturitz and Ormesson individuals predate 30,000 years ago. Together, these findings challenge the previously proposed model, which posited that the Franco-Cantabrian region and the rest of Western Europe were exclusively associated with Fournol-related ancestry ∼30 ka (*8*, *13*). Despite the constraints of a limited sample size, we used the DATES method to temporally constrain this gene flow, assuming this the result of a single event. Our analysis identifies a highly probable admixture pulse occurring between 32,500 and 31,500 cal yr B.P. This time frame would be coincident with early stages of the Gravettian in Western Europe (*42*, *43*).

Two main scenarios could explain the presence of eastern-related ancestry detected in both Isturitz and Ormesson, as well as previously documented in Goyet. In the first scenario, this signal reflects gene flow between populations associated with the western and eastern Gravettian genetic clusters during the Gravettian period itself. In the second scenario, it may derive from an earlier ancestry component predating the lineage represented by Goyet Q116-1, which could already have been present in western European Aurignacian populations.

The first scenario is supported by Isturitz’s exceptional assemblages of portable art objects, among the richest in Southern Europe and closely similar to items throughout the Pyrenean and Cantabrian regions during different Last Glacial cultural periods (*24*, *25*, *44*). This evidence reinforces the archaeological theory that the site was a key hub of cultural and biological exchange during much of the Upper Palaeolithic, located at the critical passage between continental Europe and the Iberian Peninsula at the western end of the Pyrenees. The site of Isturitz has long been recognized as a major aggregation (or “super”) site (*24*–*26*, *44*–*48*). So-called aggregation sites refer (albeit with debate) to locations (e.g., large, favorably situated caves) characterized by prolonged and intensive occupation by multiple human groups for a wide variety of purposes, including those of collective hunts, goods and information exchange, socialization, and mating. They are characterized in the Upper Palaeolithic by the presence of abundant portable artworks, often alongside rock art, and by other archaeological features such as structured living spaces with numerous hearths, large amounts of faunal remains, high artefact density and diversity, and evidence of repeated visits. This east-west genetic axis also aligns with macroregional dental morphology analyses that suggested transcontinental movements around 28 ka (*6*). Crucially, however, our genomic data argue against a major population replacement or discontinuity at this juncture. Newly reported data from Isturitz and Chufín, sediment DNA from El Mirón, and reanalyzed Western European datasets (*8*, *13*) collectively demonstrate robust regional genomic continuity in Southwest Europe between 30 and 20 ka, contradicting the 28-ka turnover inferred from dental phenotypes (*6*).

Alternatively, the signal may represent a deeper ancestral component that predated the Goyet Q116-1 lineage and was already integrated into some Western European Aurignacian populations. While the presence of Věstonice-related ancestry in pre–30-ka specimens, such as the Ormesson individual, is consistent with this deeper temporal root, its apparent absence in subsequent Fournol-associated samples after 30 ka suggests either genetic dilution or a population expansion from a source group lacking the Věstonice signal. However, two primary factors challenge this “deep-root” model. First, while similar admixture signatures exist in Belgian Gravettian individuals around 28 to 26 ka, the signal is notably absent in earlier Aurignacian samples from the same region (*8*). Second, the signal vanishes from all sampled SW European individuals post–30 ka, a pattern that supports a punctuated admixture event rather than a continuous, deep ancestral presence. Here, the degraded Chufín tooth yielded limited data (∼20,000 SNPs), it provides a critical temporal anchor at 26 ka, further validating the postadmixture genomic stability of SW Europe throughout the Gravettian. These findings may suggest that the overlap between Gravettian and Aurignacian cultures proposed in SW Europe [∼35,000 to 33,000 years before the present (yr B.P.)] (*43*), which is close in time to the proposed gene-flow pulse 32,500 to 31,500 yr B.P. may reflect a period of substantial population reorganization in Western Europe, which may explain the overlapping cultural traditions.

Last, we successfully recovered thousands of nuclear markers from a single sedaDNA sample, providing an additional temporal signal for the persistence of Fournol-related ancestry in SW Europe, which is extremely little represented during the Solutrean with only three genome-wide samples. This result highlights the capacity of sedaDNA for uncovering previously inaccessible genetic dynamics in absence of human remains (*18*, *19*, *22*, *23*) and decisively connects archeological records with human genetic identities. Several questions raised by this study remain unresolved, including the potential presence of additional admixture events and the identity of Aurignacian populations in SW Europe that predate the Isturitz Individual. These issues may be further addressed through future sedaDNA analyses. In particular, sediments from Aurignacian contexts in SW Europe, such as level C4 of the Salle Saint-Martin in Isturitz Cave, known for its rich Proto-, Early-, and Aurignacian occupations (*25*, *44*) represent promising targets. Such contexts are especially valuable, as comparable archaeological deposits remain rare across the broader region and may help clarify the origin and nature of the eastern ancestry signal detected in this study. However, sedaDNA analyses in archaeological settings also pose specific challenges, including contextualization (*21*), the potential mixing of genetic material from multiple individuals, and the need to maximize endogenous DNA recovery as we have shown in this work.

Overall, our results suggest that population structure and gene flow in western Europe during the Gravettian were more complex and dynamic than previously assumed, in line with recent findings (*6*, *8*). The presence of eastern Gravettian ancestry in SW Europe before the LGM challenges the prevailing model of genetic continuity from the Aurignacian to the Solutrean in this region. Future work integrating additional skeletal genomes and sedaDNA datasets will be essential to further resolve the processes that shaped the genetic landscape of Upper Palaeolithic populations in Europe.

## MATERIALS AND METHODS

### Provenance

The long bone from Isturitz was sampled at the Institut de Paléontologie Humaine (IPH; France) in 2013 by Ron Pinhasi. The bone had been deposited at the institute following excavations carried out during the 1950s. It was directly radiocarbon dated at the University of Oxford. After sampling, the bone was returned to the IPH in accordance with the terms of the permit. The Chufín tooth was discovered during excavations conducted by the team of Diego Garate in 2021 and was sampled in Vienna, where it was directly radiocarbon dated. The remaining material was subsequently returned to the University of Cantabria. The bone from Ostuni1b was sampled at the Ostuni Museum in 2023 by Alfredo Coppa. Its date was inferred through its kinship relationship with Ostuni 1, and the remaining material was returned to the Ostuni Museum. Sediment samples from El Mirón (excavations directed by Manuel Gonzalez Morales, Lawrence Straus, Igor Gutierrez-Zugasti, and David Cuenca-Solana) were collected in 2023 by Pere Gelabert, and the remaining material is now stored at the University of Vienna. All sampling and analyses were conducted under the required permits and complied with all relevant authorization requirements.

### Experimental methods

A complete description of the methods is presented in the Supplementary Materials. ElMiron14 (Spain), Chufín (Spain), and Ostuni1b (Italy) were processed entirely at the Palaeogenomics Laboratory of the University of Vienna (Austria), in dedicated clean-room facilities, following strict contamination control protocols. The Isturitz (France) sample was processed jointly at the University of Vienna and Harvard Medical School (USA) using coordinated procedures (Fig. 1). We applied contamination control measures to mitigate the effect of modern DNA contamination. We included negative controls at each step of the wet lab pipeline to control for potential contamination of reagents. Fifty miligrams of sediment or bone was extracted per sample following the protocol of Dabney and Meyer (*49*) with adaptations from Korlević *et al*. (*50*). Sample ElMiron14 was extracted twice from two different 50-mg aliquots to increase the chances of retrieving human DNA. The DNA was eluted in 50 μl of TET buffer [10 mM tris-HCl, 1 mM EDTA, and 0.05% Tween 20 (pH 8.0)], and double-stranded libraries were prepared from 25 μl of the extract, as described by Meyer and Kircher (*51*), without shearing the DNA into smaller fragments. We used a MinElute PCR Purification kit from QIAGEN to clean up the samples and eluted in 40 μl of EBT buffer (1 mM EDTA and 0.05% Tween 20). The number of polymerase chain reaction (PCR) cycles per sample was determined through real-time PCR. Libraries were double indexed (*52*) and amplified with PfuTurbo Cx HotStart DNA Polymerase from Agilent. A subsequent cleanup was performed with 1.2× NGS cleanup magnetic beads per sample, introducing a size selection. We eluted in 25 μl of EBT buffer (1 mM EDTA and 0.05% Tween 20). Each indexed library was further amplified in preparation for in-solution capture using KAPA HiFi HotStart DNA Polymerase and IS5/IS6 primer pairs (*51*). We enriched some of the libraries (tables S1 to S3) with the 1.4 million reads from TWIST. We followed the protocol “Protocol_NGS_HybridizationTE_31OCT19_Rev1” from TWIST with the changes for aDNA detailed in (*29*). As we were pipetting manually, we washed the bead-bait combination four times with wash buffers as detailed in (*29*). After denaturing the target DNA from the probes in a PCR cycler at 95°C for 5 min, we performed another quantitative PCR before amplifying half of the captured library using KAPA HiFi HotStart DNA Polymerase and the primer pairs IS5/IS6. We cleaned up with magnetic beads. Quality controls were performed using Qubit and TapeStation. The captured libraries were sequenced at the Vienna BioCenter Core Facilities (VBCF) on an Illumina NovaSeq 6000 platform.

### Alignment of genomic data

Sequencing reads were trimmed using cutadapt (version 1.2.1) (*53*) with a minimum length of 30 and removal of polyA tails. We also processed the sequencing reads using fastp 0.25.0 (*54*) with the following parameters (--trim_poly_g --trim_poly_x -poly_x_min_len 5 -poly_g_min_len 5 --length_required 30 --qualified_quality_phred 30). This pipeline ensured the retention of high-quality reads. The resulting reads were aligned to the human reference genome (GRCh37) and the revised Cambridge reference sequence (rCRS) mitochondrial genome using BWA aln (version 0.7.5) (*55*), with seeding disabled and a gap penalty of 2 set for the open end. Duplicate mapped reads were removed using Picard Tools 2.21.4 (*56*) with default parameters. Reads with mapping qualities below 30 were also removed. Unique and filtered reads were analyzed with Qualimap-2.2.1 (*57*) to assess whole-genome coverage depth. MapDamage-2.0.9 (*58*) was used to estimate deamination levels and assess the authenticity of the data.

### Processing of ElMiron14 data

For ElMiron14 reads, we converted the resulting BAM files into FASTA files using Samtools 1.12 (*59*). These sequences were processed with BLASTn 2.16.0 (*60*) using the National Center for Biotechnology Information (NCBI) whole-nucleotide database (7 June 2024). The results were analyzed in MEGAN 6.25.10 (*61*) using the following parameters: native LCA, bitscore of 50, minimum identity of 80, and top score 10, grouping the Hominidae reads. The resulting reads were subtracted from the original BAM file and realigned against the human reference using the same parameters.

For the mtDNA (tables S3 and S4), we used the same methodological approach described by Gelabert *et al.* (*22*), capturing with commercial myBaits mtDNA baits. These libraries were sequenced in paired-end 150 reaction reads on a NovaSeq X platform, VBCF. The reads were then clipped using cutadapt (version 1.2.1) with a minimum length of 30 and removal of polyA tails. The clipped reads were then collapsed with PEAR 0.9.11 (*62*) with a minimum overlap of 11 bases. The resulting reads were aligned against the rCRS mtDNA reference genome using BWA aln (version 0.7.5) (*55*), with seeding disabled and a gap penalty of 2 set for the open end. Duplicate mapped reads were removed using Picard Tools 2.21.4 (*56*) with default parameters. Reads with mapping qualities below 30 were also removed. Unique and filtered reads were analyzed with Qualimap-2.2.1 (*57*) to assess whole-genome coverage depth. MapDamage-2.0.9 (*58*) was used to estimate deamination levels and assess the authenticity of the data. The reads were then converted to fastq using Samtools 1.12 (*59*). These sequences were processed with BLASTn 2.16.0 (*60*) using the NCBI whole-nucleotide database (7 June 2024). The results were analyzed in MEGAN 6.25.10 (*61*) using the following parameters: native LCA, bitscore of 50, minimum identity of 80, and top score of 5, grouping the primate reads. These were aligned again to the human reference genome and processed with Schmutzi 2.0.0 (*63*) to determine the consensus sequence. Last, we examined each position with Samtools mpileup and called the most abundant haplogroup with Haplogrep 3.2.1 (*64*). We plotted the deamination and read length distributions. We also examined the sequences manually counting each of the alleles in the segregating positions (table S4). We used quicksand 2.6 (*65*) following the authors specifications to classify the rest of the mtDNA diversity present in the sample to explore the faunal and floral diversity present in the newly generated reads (section S5).

### Quality control and postprocessing

To determine the presence of contamination, we applied three methods based on mtDNA diversity: Schmutzi 2.0.0 (*63*), ContamMix (*7*), and Calico 2.0 (*66*). In the case of Ostuni1b, we also used angsd 0.941 (*67*) to determine contamination in the X chromosome. After this contamination determination, we processed the reads from Chufín and ElMiron14 with pmdtools 0.60 (*68*), keeping those with PMD scores greater than 3. Last, we used GATK 3.8.1.0 (*69*) to realign indels using RealignerTargetCreator and IndelRealigner, along with Mills_and_1000G_gold_standard.indels.b37.vcf database. We then determined deamination using MapDamage 2.2.2 (*58*). We trimmed the reads accordingly using bamutil-1.0.15’s trimbam option (*70*), removing bases until the last base had <5% damage (9 bases for Miron14 reads and 10 bases for Chufín). Genotypes were called using the pileup caller of sequenceTools (*71*), filtering out bases with a base-quality score below 30. At this stage, we called genotypes at the positions of the 1240k SNP targets. These calls were merged with ancient and present-day individuals (table S5 and section S5) using PLINK 1.9 (*72*). The full list of samples is available in table S5.

We generated consensus sequences of the mtDNA genomes using Schmutzi 2.0.0 and predicted the mtDNA haplogroup using Haplogrep 3.2.1 (*64*). We predicted the Y chromosome of the Ostuni1b individual using Yleaf v3.2.1 (*73*), restricting the analysis to positions with three reads.

### Population genetic analyses

We used 1074 individuals from Western Eurasian populations from the AADR repository (*74*), genotyped at 597,573 positions on the Affymetrix Human Origins SNP array, to perform Principal Components Analysis with smartPCA (*39*). In this PCA, we projected all the samples we report in this paper and other relevant Palaeolithic samples (table S5). We used the same dataset to perform *f* statistics–based analyses using AdmixTools 8.0.2 (*39*) (tables S6 and S7), following the standard protocols in paleogenomics. The results were plotted using R 4.4.2 (*75*). We constructed an *f*_3_ matrix using all samples with more than 10,000 SNPs and grouped the samples based on former publications. The results were processed with R to build an MDS plot. We also used AdmixTools 8.0.2 to compute *f*_4_-statistics comparisons, and we plotted the ones with more than 4000 SNPs used.

We performed an admixture graph search using the find_graph() function from ADMIXTOOLS 2.0.2 (*40*). To broadly sample topology space and reduce the risk of convergence to local optima, we performed 20 independent searches, each allowing up to two admixture events (max_admix = 2), rooting the graph with *Pan troglodytes* (“Chimp.REF”), and using stop_gen = 50 generations with numgraphs = 50 candidate graphs per generation. Each run was initialized with a different random seed and parallelized across all available CPU cores. All results were combined, and model fit was ranked using the score returned by find_graphs() (negative log-likelihood, with lower values indicating better fit). The best-fitting topology was defined as the single graph with the lowest score across all runs, and we quantified how often this topology recurred among all candidate graphs. The best model and all results were saved for reproducibility, and we visualized the winning topology. The initial tree structure was informed by the model presented in Posth *et al.* 2023 (*8*). Last, we used DATES_v4010 (*76*) to estimate the admixture dates of the identified gene flow events using 0.001 bins and a maximum distance of 1 cM, we run both jackknife to estimate the SEs and runfit for dating.

We applied the ROH analysis approach in (*77*), but only to the Ostuni1b and Isturitz data. We plotted the results with the Python scripts at (https://github.com/hringbauer/hapROH) together with other Gravettian and Aurignacian individuals. We used the hapROH results to predict the effective population sizes of the studied individual populations following the instructions from hapROH, excluding individuals with recent inbreeding. We used aHISplex (*41*) (https://github.com/zmaroti/aHISplex) to predict the eye, hair, and skin color of the newly reported samples.

## Supplementary Materials

### The PDF file includes:

Supplementary Text Figs. S1 to S15 Legends for tables S1 to S8 Tables S9 to S29 References
