# Supplementary material for: Early Gravettian genomic data from Western Europe reveal population movements before the LGM
Source: Sci Adv. 2026 Oct 23;12(43):eaeh6128. doi: 10.1126/sciadv.aeh6128 (PMC13693654; doi:10.1126/sciadv.aeh6128)
Supplement: Supplementary file 1 [file sciadv.aeh6128_sm.pdf]

Science Advances
AAAS

Supplementary Materials for

Early Gravettian genomic data from Western Europe reveal population movements before the LGM

Pere Gelabert *et al.*

Corresponding author: Pere Gelabert, pere.gelabert@univie.ac.at; Ron Pinhasi, ron.pinhasi@univie.ac.at

Sci. Adv. **12**, eaeh6128 (2026)
DOI: 10.1126/sciadv.aeh6128

The PDF file includes:

Supplementary Text
Figs. S1 to S15
Legends for tables S1 to S8
Tables S9 to S29
References

Other Supplementary Material for this manuscript includes the following:

Tables S1 to S8

Supplementary Text

1. Cave of Chufín

1.1 Sample context

The Cave of Chufín is located in the valley of the river Nansa, about 20 kilometres from the present-day sea shore. The archaeological value of Chufín cave became known with the discovery of the first rock art in 1972. Immediately, the site was excavated between 1974 and 1976, and the rock art was studied (*14*, *27*). In 2021, archaeological excavations were resumed by one of us (DGM) to define the site's stratigraphic sequence better and characterise the human occupations that took place there. Initially, two test pits were opened next to the previous excavation areas and to the rock art walls to identify intact sediments. During this first campaign, the human tooth (CHU.53k.3.02) sequenced in this study was recovered from layer 201 of the right sector of the excavation. It was located in a superficial level next to the parietal engravings and associated with archaeological material mainly attributed to the Solutrean period. However, the direct dating of the tooth is slightly older.

The tooth (Fig. S6) was radiocarbon-dated in 2023. The sample was prepared and chemically pretreated at the Higham Lab at the University of Vienna, Austria. Collagen was extracted using a modified Longin collagen method outlined in (*78*) and (*79*). The sample was gelatinised in weakly acidic pH3 water and ultrafiltered using a 30kD Sartorius ultrafilter, before being freeze-dried. Circa 0.3 mg of collagen was measured for $\delta^{13}C$ and $\delta^{15}N$ values using an Elemental Analyser-Isotope Ratio Mass Spectrometer (EA-IRMS) at the Faculty of Life Sciences Silver Laboratory to a precision of ±0.3‰ relative to V-PDB and AIR, respectively. Stable isotope analysis revealed a $\delta^{13}C$ value of -20.1‰ and $\delta^{15}N$ value of 11.68‰, while the atomic C/N ratio was 3.55, which suggests the collagen was of acceptable quality. The remaining collagen was combusted and graphitised with the AGE3 system. The sample was measured at the VERA (Vienna Environmental Research Accelerator) AMS facility and produced a radiocarbon age of 22793 ± 116 yr BP (VIE-1682), which is calibrated 27330-26500 cal BP, falling completely in the Gravettian in the Cantabrian region.

1.2 The sequencing of Chufin tooth

All the analytical procedures were performed at the Paleogenomics laboratory of the University of Vienna. We made two separate samples from Chufin tooth corresponding to powders 12293 and 15022; each powder was extracted once, and two double-stranded libraries were made from each powder. We then sequenced these libraries using different methods and fractions, resulting in 46 sequencing runs. This includes: 11 mtDNA capture samples, 13 shotgun, and 22 subsamples processed with TWIST Ancient DNA in-solution capture (*31*). The samples captured with the TWIST capture have an absence of mitochondria reads, likely because the reagent does not target this part of the genome, as the probes were not successfully included (Table S2). It is apparent from the table that the reads obtained from powder 12293 display anomalous values across several standard sequencing metrics, including unusually elevated edit distances, suggesting the presence of heterogeneous sequence sources or substantial contamination. In all samples derived from this powder, the edit distance exceeds 2, while the deamination rate is approximately 15%. Although this level of deamination is compatible with authentic ancient DNA, its combination with consistently high edit distances indicates a mixed dataset, potentially reflecting a combination of endogenous molecules, contaminant DNA, and/or poorly aligned reads. The consistency of these patterns across all libraries generated from powder 12293 further suggests that the issue originates from the source material rather than from downstream laboratory procedures.

To investigate the possible causes of these anomalies, we conducted additional analyses. First, we performed a BLAST search to identify potential non-human contaminants; however, no clear non-human sources were detected. Estimates of human contamination using different software tools, including Schmutzi 2.0 (*63*), ContamMix (*7*), and Calico (*66*), yielded values of approximately 3–5%. While these levels are relatively low and cannot fully explain the observed anomalies, the possibility of substantial contamination affecting the nuclear genome cannot be ruled out.

Given the difficulty in disentangling these signals and ensuring data authenticity, we excluded all sequencing data derived from powder 12293 from further analyses and we focus on the reads originating from powder 1502.

1.3 Chufin's Mitochondrial Genome

Next, we merged all the reads from powder 15022 and subsequent extract E3031, which includes libraries l2517 and l2518 (17507_iL2amp_2, 17507_iL2.3_reamp, 17507_iL2.4_reamp, 1_L_17507_iL2.2Mb_trio_1CRA, 2_L_17507_iL2.3Mb_trio_1CRA, 3_L_17507_iL2.3Mb_trio_1CRA, 17507_iL2.2_5CRA, 17507_iL2.3_5CRA, 17507_iL2.4_5CRA, 17507_iL2, 17507_iL2.2_re-amp, 17507_iL2.3_re-amp, 17507_iL2.4_re-amp, 17507_iL2_re-amp, 17507_iL3amp_2, 17507_iL3.2_amp, 17507_iL3.3_amp, 17507_iL3.4_amp, 4_L_17507_iL3.2Mb_trio_1CRA, 5_L_17507_iL3.3Mb_trio_1CRA, 6_L_17507_iL3.4Mb_trio_1CRA, 17507_iL3.2_5CRA, 17507_iL3.3_5CRA, 17507_iL3.4_5CRA, 17507_iL3.2_re-amp, 17507_iL3.3_re-amp, 17507_iL3.4_re-amp). After aligning the reads to the reference genome and applying filters for mapping quality (>30) and minimum read length (≥30 bp), we recovered a total of 261,907 unique reads mapping to the mitochondrial genome after duplicate removal, resulting in an average coverage of approximately 1025× across the entire genome. To assess potential contamination, we applied three complementary approaches, Calico, contamMix, and Schmutzi, first on individual sequencing attempts, then on each separated library (Table S2, S9-S11), and finally on the merged dataset.

Contamination estimates for the merged data were as follows: 0.056 (95% CI: 0.048–0.064) using Calico, 0.02 (95% CI: 0.01-0.03) using Schmutzi, and a consensus sequence match of 0.95 (95% CI: 0.92-0.97), corresponding to approximately 5% contamination, using contamMix. These results indicate a generally low level of exogenous human DNA in the dataset. We concluded that the most likely scenario is that the sample shows contamination in a range between 5% to 10%. These contamination estimates require that the nuclear data be filtered by damage to avoid including contaminant reads in the population genetics analyses.

We also evaluated the contamination levels in the blanks generated during the preparation of Chufin sequencing libraries (EB_ChufinTC_iL1/2517, LB_ChufinTC_iL1, EB6_BT, LB5_BT). These blanks contained detectable amounts of human DNA; however, the number of reads remaining after filtering (Table S2) was very low, already indicating exhaustion of the libraries. Furthermore, the reads from these blanks did not exhibit signs of cytosine deamination, suggesting that they are unlikely to interfere with downstream analyses. As all samples were

processed with PMD filtering tools, any residual exogenous sequences are not expected to affect subsequent workflows or inferences.

We used the merged mitochondrial reads to determine the haplogroup with Haplogrep3 (*64*), and determined it to be U8, and this is consistent across all the sequencing runs (Table S2). All the libraries showed the same mutations (against rCRS) which codify for Haplogroup U8: 73G 263G 315.1C 750G 1438G 1811G 2706G 3107C 4769G 6455T 7028T 8860G 9698C 11467G 11719A 12308G 12372A 13145A 14766T 15326G 16093C 16129A 16569A. Haplogrep predicts this haplogroup with 94% support. As the individual is female, no contamination estimate based on the X chromosome could be performed.

1.4 The Nuclear data

As mentioned before, we merged all the reads from powder 15022 and subsequent extract E3031, which includes libraries l2517 and l2518 (17507_iL2amp_2, 17507_iL2.3_reamp, 17507_iL2.4_reamp, 1_L_17507_iL2.2Mb_trio_1CRA, 2_L_17507_iL2.3Mb_trio_1CRA, 3_L_17507_iL2.3Mb_trio_1CRA, 17507_iL2.2_5CRA, 17507_iL2.3_5CRA, 17507_iL2.4_5CRA, 17507_iL2, 17507_iL2.2_re-amp, 17507_iL2.3_re-amp, 17507_iL2.4_re-amp, 17507_iL2_re-amp, 17507_iL3amp_2, 17507_iL3.2_amp, 17507_iL3.3_amp, 17507_iL3.4_amp, 4_L_17507_iL3.2Mb_trio_1CRA, 5_L_17507_iL3.3Mb_trio_1CRA, 6_L_17507_iL3.4Mb_trio_1CRA, 17507_iL3.2_5CRA, 17507_iL3.3_5CRA, 17507_iL3.4_5CRA, 17507_iL3.2_re-amp, 17507_iL3.3_re-amp, 17507_iL3.4_re-amp).

All the reads were aligned to the hg19 reference of the Human genome. After aligning the reads and filtering for a mapping quality above 30 and a minimum length of 30 bp, we recovered 2,518,244 filtered reads (Table S2). We then removed duplicates between the whole set, resulting in 2,518,244, as no duplicates were found (meaning that no molecules were shared between libraries). Based on the estimates of contamination that may point to 5 to 10% of contamination, we decided to restrict the analysis to damaged reads using PMDtools 0.60 (*68*). This resulted in 491,802 reads. We trimmed 10 bp per end following the mapDamage results using trimbam from bamUtil 1.0.15 (*80*), which decreases the included ancient DNA damage to below 5%. We then called pseudo-haploid genotypes using pileupCaller from sequence tools 1.5.4.0 (*71*)), which

produced 19,895 SNPs of the 1240k dataset. We used the differential proportion between the X and Y chromosomes, which yielded a 15-fold difference, to infer that the tooth belongs to a female.

We then explored the differences between the sequencing runs of the Chufín sample (Table S2). We paid special attention to identifying differences between the shotgun and capture strategies in the sample. We found that the **Edit Distance (ED)**, which measures the number of substitutions in the reads relative to the reference, was lower in capture runs than in shotgun runs (mean = 0.693, SD = 0.0320 vs. mean = 0.765, SD = 0.0217; Mann–Whitney U test, $p = 0.002$).

We also observed that **deamination at the 5′ end** (C→T transitions) was higher in shotgun samples than in capture samples (mean = 0.274, SD = 0.00386 vs. mean = 0.158, SD = 0.00855; Mann–Whitney U test, $p = 0.001$).

The most pronounced difference was observed in **read length**, with longer reads in capture runs compared to shotgun runs (mean = 73.5 bp, SD = 1.15 vs. mean = 57.5 bp, SD = 2.17; Mann–Whitney U test, $p = 0.001$).

We also observed a higher overall number of reads recovered in shotgun experiments; however, the **median number of reads was higher in capture experiments** (Table S12; Fig. S9). These results are likely influenced by the previously detected contamination.

The capture design used here, the TWIST Ancient DNA design, uses a single 80-bp probe on each targeted SNP (*31*). Because modern DNA contaminants typically consist of longer fragments than authentic ancient DNA, the long probes in the TWIST panel may preferentially capture these longer molecules, potentially leading to an apparent increase in modern DNA contamination. This effect could account for many of the differences observed in the previous contamination estimates, as visualised in Fig. S9. Consistently, we observe that TWIST Ancient DNA probes tend to recover longer, less deaminated sequences, supporting the notion that this design may amplify the signal of modern contamination relative to authentic ancient molecules.

2. Isturitz cave

Isturitz Cave is part of a karstic network running through Gaztelu Hill in the Arberoue Valley (Basque Country, France) It is located near the western end of the Pyrenees at the key corridor connecting SW France and northern Atlantic Spain at the corner of the Bay of Biscay. Discovered in 1896, the site has been the focus of archaeological research from 1912 to the present, with a notable slowdown between the early 1960s and the mid-1990s. Human occupation at the site began during the Mousterian and extended through nearly the entire Upper Palaeolithic. The density and richness of certain archaeological layers suggest that the cave may have served as a major gathering place at various points in time, and it shows one of the most outstanding art records in Southwest Europe(*81*).

In this vast cave, the Gravettian occupation has been primarily documented in the Isturitz Chamber, also known as the *Grande Salle*, covering an estimated area of around 700 square meters. Two layers were identified by early excavators. The oldest and richest, known as Ist IV and explored by R. and S. de Saint-Périer, has been attributed to the Noailles burin phase of the Gravettian. It yielded over 11,000 lithic tools and nearly 1,400 objects made from hard animal materials. However, recent studies suggest that this represents only a small fraction of the original assemblage. Nonetheless, the material clearly reflects intense and repeated human occupation over an extended period. More broadly, the Gravettian presence at Isturitz Cave appears to fall within a wide chronological range, from at least 32,000 to 28,000 calibrated years BP.

A fibula fragment IS-02A (1950-10-1) (Fig. S7) was discovered in 1950 from stratigraphic level IV. The bone is dated to 28,897-28,022 calBCE (26030±220 BP, OxAX-2616-50), a relatively late phase of the Gravettian at Isturitz. The sample was processed at David Reich's laboratory in Harvard University, preparing 8 UDG-treated libraries captured with TWIST Ancient DNA probes (Table S1). In total, 454,258 SNPs covering the 1240k capture kit were recovered, and no signs of contamination were detected. We used the differential proportion between X and Y chromosomes to infer that the tooth belongs to a female.

The individual is characterised as a female of haplogroup M. We determined that this individual has a 0.019930 (SE=0.007326) Z=2.7 of Neanderthal, consistent with the rest of the individuals from the period (*82*). As we recovered a significant number of SNPs, we also ran hapROH following the recommended pipeline (*83*), and we identified no ROH>4 cM in the genome.

3. Ostuni1b

The Upper Palaeolithic funerary complex of Ostuni, situated in the Santa Maria di Agnano cave (Ostuni, Apulia, Southern Italy), was discovered in 1991 by one of the authors of the present paper (DC). It yielded two primary burials, namely Ostuni 1 and Ostuni 2. Ostuni1 (Os1) individual has been dated to 27,810-27,430 cal BP and was genetically studied in Fu et al, 2016 (*7*). The Ostuni 1 burial contained the exceptionally well-preserved skeleton of a young woman, who was no older than 20 years and in a late stage of pregnancy at the time of death (*28*, *84*). The body was richly decorated with hundreds of perforated marine shells around the wrists and the cranium[18]. She was positioned on her left side in a crouched position, with the right forearm around her abdomen. Within the pelvic region of Os1, an almost complete foetal skeleton - Os1b - was recovered in excellent preservation, with the joint in connection and in the position typically acquired during the later stages of pregnancy. The Ostuni 2 burial contained a poorly preserved adult skeleton (Os2) of indeterminate sex and dated to 26339-25779 cal BC (*28*).

The study of the dental remains of the fetus through virtual histomorphometry has indicated that it died at 31-33 gestational weeks. The same study reports Os1b dental growth trajectories, suggesting a fetal developmental timing for Os1b slightly faster than in contemporary fetal individuals. Three physiological stress episodes were identified in the enamel, which formed during the last two and a half months of life, suggesting that both the Os1 mother and Os1b fetus were under severe physiological stress during the last period of life. These stressors possibly resulted in the death of both the mother and the child (*85*).

To generate data from this individual, we sampled the left petrous bone following the protocol described in Pinhasi et al 2019 (*86*). DNA was extracted, and we prepared two libraries from the E2942 extract: Library L5092 was treated with USER enzyme treatment (NEB) to reduce the damage (*87*). Library L5093 was not treated. For each of the libraries, one fraction was

sequenced using Shotgun Sequencing, and one was captured with the TWIST Ancient DNA capture (*31*) (Table S1). To obtain abundant mtDNA data, we also captured library L5093 with an mtDNA capture kit. We assessed the presence of contamination in both libraries using Calico, Schmutzi and ContamMix, and we did not identify the presence of contamination in the mtDNA genome (Table S1). We determined that the presence of modern contamination in the nuclear genome was below 1% using ANGSD (*67*). We used the differential proportion between X and Y chromosomes, which resulted in a 15-fold difference, to infer that the tooth belongs to a male.

The sequencing blanks used in the experiments (EB_AH1imtL1 [NegPoolAHmt] and LB_AH1imtL1 [NegPoolAHmt]) were also analysed. Although the extraction blank (EB) shows the presence of modern DNA, the mitochondrial genome is already highly saturated in the sample, indicating that any potential contribution from this source would have only a minimal effect on the results. Furthermore, independent contamination estimates based on both mitochondrial and nuclear DNA indicate that the sample does not show evidence of modern DNA contamination (Table S1).

Following previous observations, we did not restrict the base calling to damage-prone reads. To reduce the impact of damage on base calling, we clipped 5 base pairs from each end of nuclear reads, effectively reducing damage levels to below 5%. After processing, we recovered a total of 467,368 SNPs, covering the 1240k dataset.

To confirm that the fetus is biologically linked to the female adult Ostuni 1, we ran READ v2, which indicated that they are consistent with a first-degree relationship (Fig. S8).

4. <u>El Mirón Cave sediments</u>

<u>4.1 Sediment Processing from El Mirón Cave</u>

ElMiron14 is a soil sample from square W10 of level 122 in El Mirón Cave, excavated under the direction of L.G. Straus and M.R. González Morales and dated to 22,270–21,660 calBP (*31*, *88*), corresponding to the Late Solutrean period in Cantabria. This large cave, with a culture-stratigraphic sequence spanning from the Mousterian to the Bronze Age, is located on the northern edge of the Cantabrian Cordillera, about 20 km from the present shore of the Bay of

Biscay. In a previous study where this sample was enriched using mtDNA baits (*22*), we found that it contained substantial amounts of human DNA, and is the sample with the highest number of human reads from the ones screened to date from El Mirón Cave.

We extracted DNA from the sediments two times using 50mg of homogenised sediment each time. These are extracts E1987 and E2668. From E2987, we prepared libraries l1980 and l2594. From E2668, we prepared libraries l2236, l2588, l2589, l2590, l2591, l2592 and l2593 . Different fractions of these libraries were indexed several times, followed by capture with TWIST Ancient DNA in-solution capture (*31*) for some of the samples, and shotgun sequencing for others(*31*) at the Vienna BioCenter Core Facilities (VBCF) on an Illumina NovaSeq 6000 platform. The complete list of samples and their treatments is available in Table S3.

Sequencing reads were trimmed using cutadapt (version 1.2.1)(*53*) with a minimum length of 30 and removal of polyA tails. We also processed the sequencing reads using fastp 0.25.0 (*54*) with the following parameters (--trim_poly_g --trim_poly_x -poly_x_min_len 5 -poly_g_min_len 5 --length_required 30 --qualified_quality_phred 30). This pipeline ensured the retention of high-quality reads. The resulting reads were aligned to the human reference genome (GRCh37) and the revised Cambridge reference sequence (rCRS) mitochondrial genome using BWA aln(*55*) (Version 0.7.5), with seeding disabled and a gap penalty of 2 set for the open end. Duplicate mapped reads were removed using Picard Tools 2.21.4 (*56*) with default parameters. Reads with mapping qualities below 30 were also removed. Unique and filtered reads were analysed with Qualimap-2.2.1 (*57*) to assess whole-genome coverage depth. MapDamage-2.0.9 (*58*) was used to estimate deamination levels and assess the authenticity of the data. We converted the resulting BAM files into FASTA files using Samtools 1.12 (*59*). These sequences were processed with BLASTn 2.16.0 (*60*) using the NCBI whole-nucleotide database (2024-06-07). The results were analysed in MEGAN 6.25.10 (*61*) using the following parameters: Native LCA, bitscore 50, minimum identity of 80, and top score 10, grouping the Hominidae reads. The resulting reads were subtracted from the original BAM file and realigned against the human reference using the same parameters. All the values are presented in Table S3. Altogether, 1,028,866,146 reads were generated across all libraries. Of these, 652,257 were

classified as Hominin based on BLAST results, and finally, 283,315 of these could be confidently attributed to human ancestry.

4.2 Diversity between the samples

After filtering and removal of PCR duplicates, an average of 3,018 reads per library were assigned to humans in the capture experiments, compared to 2,779 reads in the shotgun libraries. Statistical comparison using the Mann–Whitney U test showed no significant difference in the number of human reads recovered between the two sequencing strategies (W = 1040.5, P = 0.636; Fig. S9).

However, a marked difference was observed in the number of informative SNPs recovered across the 1.4M panel. The combined 1.4M capture libraries produced 48,384 informative positions, compared to only 1,515 positions recovered from shotgun sequencing across the same genomic range. This represents a 32-fold increase in data recovery using capture (or 128-fold when accounting for sequencing yields; Table S3). These results highlight the limitations of shotgun sequencing for sedimentary ancient DNA (sedaDNA) samples when the objective is to recover genome-wide human variation.

Despite similar numbers of human reads, other sequencing characteristics differed significantly between capture and shotgun libraries. Statistical comparisons revealed differences in edit distance ($p = 3.392 \times 10^{-8}$), read length ($p = 4.263 \times 10^{-16}$), and deamination levels in recovered reads ($p = 5.483 \times 10^{-16}$). In general, capture-derived reads were longer and exhibited lower levels of deamination than shotgun-derived reads.

To verify that these observations were not due to laboratory contamination, we examined the sequencing blanks used in these experiments (LB_180723, LB_130723, EB_16_10_23_OM, and LB_16_10_OM). No evidence of significant contamination was detected in these controls, suggesting that any modern DNA signal detected in the samples most likely originates from the sample context itself (Table S3), which may explain some of the differences observed between sequencing approaches.

To further investigate variation within the dataset, we explored the presence of diversity across the 101 sequenced libraries using four parameters: read edit distance (ED), read length distribution, 5′ deamination levels, and sequencing yield. For each parameter, we computed the mean, median, and standard deviation, and tested for statistical differences using the Mann–Whitney U test or Kruskal–Wallis test as appropriate. All analyses were conducted in R 4.4.2 (*75*).

A) Edit distance

We first examined differences in edit distance across sequencing attempts grouped by extract and library (Tables S13-S14). Edit distance measures the number of nucleotide substitutions required to transform a read into its best-matching reference sequence and therefore provides a summary of how closely sequenced molecules resemble the expected target sequence. Systematic shifts in ED across extracts or libraries could reflect differences in sequence composition or authenticity, such as variation in endogenous content, fragment damage patterns, or the presence of non-target molecules.

Across extracts (Table S13), the computed statistics were similar. A Mann–Whitney U test returned a p-value of 0.469, indicating no statistically significant difference in edit distance between extracts.

In contrast, differences were observed among libraries (Table S14). A Kruskal–Wallis test indicated significant variation across libraries ($\chi^2 = 36.236$, df = 8, $p = 1.59 \times 10^{-5}$). However, because no differences were observed at the extract level, we do not interpret these library-level differences as evidence that specific libraries are problematic.

B) Read length distribution

We next evaluated potential biases in read length, which could suggest differential levels of modern contamination (Tables S15-S16).

When grouped by extract (Table S15), the Mann–Whitney U test produced a p-value of 0.011, indicating a statistically significant difference in read length between extracts.

However, when read lengths were compared across libraries (Table S16), no significant differences were observed (Kruskal–Wallis $\chi^2 = 12.234$, df = 8, $p = 0.141$).

C) Deamination patterns

We then assessed differences in cytosine deamination at the 5′ ends of reads, a characteristic signature of ancient DNA damage (Tables S17-S18).

Across extracts (Table S17), the Mann-Whitney U test yielded a *p*-value of 0.699, indicating no statistically significant difference in 5′ deamination levels.

Similarly, no significant differences in damage levels were detected across libraries (Table S18; Kruskal-Wallis $\chi^2 = 8.7986$, df = 8, $p = 0.360$).

D) Human read recovery

Finally, we examined differences in the number of human reads recovered across extracts and libraries (Tables S19-S20).

At the extract level (Table S19), the Mann–Whitney U test showed a highly significant difference in human read recovery ($p = 3.75 \times 10^{-8}$).

However, no statistically significant differences were detected across libraries (Table S20; Kruskal–Wallis $\chi^2 = 8.7986$, df = 8, $p = 0.3596$).

E) Comparison between sequencing strategies

After evaluating variation across extracts and libraries, we repeated the same analyses by grouping sequencing attempts according to sequencing strategy (capture vs shotgun) (Table S3; Tables S21-S24).

Edit distance: Capture libraries exhibited higher edit distances than shotgun libraries (Table S21). A Mann–Whitney U test returned $p = 2.86 \times 10^{-8}$, indicating a statistically significant difference between sequencing methods.

Read length: Read length also differed strongly between sequencing strategies (Table S22). Capture reads were substantially longer than shotgun reads (mean 63.4 bp vs 48.6 bp), and the Mann–Whitney U test showed a highly significant difference ($p = 4.26 \times 10^{-16}$).

Deamination: Deamination levels were also significantly different (Table S23). Shotgun reads showed higher 5′ deamination (mean 0.481) compared to capture reads (mean 0.376), with a Mann–Whitney U test p-value of 5.48×10^{-16}.

Human read recovery: Despite these differences in molecular characteristics, the number of human reads recovered did not differ significantly between sequencing strategies (Table S24). The Mann-Whitney U test yielded $p = 0.636$, indicating comparable levels of human read recovery between capture and shotgun libraries.

All analyses described above are summarised visually for the El Mirón 14 and Chufín samples in Fig. S9.

We then performed several tests to observe and quantify the differences in our merged files: Merged_Shotgun, Merged_Capture, Merged, and Merged_under90bp. We then called pseudo-haploid genotypes using PilupCaller from Sequencetools 1.6.0.0 (*71*). The results are presented in Table S25.

All the past observations indicate differences between shotgun and capture genomes; these differences are probably linked to a major attraction of TWIST baits, for longer sequences with less deamination, which would tend to amplify the modern contamination.

Following the observations of the deamination values, we clipped five reads per read end using trimbam from bamutil-1.0.15 (*70*). The resulting final Merged file has 283,315 reads, an average fragment length of 59bp, and 40% of damage.

We first used *f3*-statistics in the form *f3*(X,Y: Mbuti) to determine the similarity between the different comparisons of the Miron sample. The results suggest the presence of modern contamination, as the sample from ElMiron14 shows more attraction for the Human Reference than the other samples (Table S26).

Without proper contamination estimates due to the multi-individual origin of the ElMiron14 reads, but with the evidence from the previous analyses of the presence of modern contamination (Fig. S9, Table S26), we decided to apply a PMD restriction using PMD tools 0.60(*68*). The process resulted in 95,843 reads. We then trimmed 9 bases per read end. The process resulted in 14,737 SNPs covering the 1240k SNP target set. These highly confident SNPs are the ones we used for the ancestry determination of the ElMiron14_pmd sample (Fig. S10).

4.3 Sex Determination

We assessed sex bias by calculating the ratio of X-chromosome coverage to total chromosomal coverage. Across all 22 chromosomes, ElMiron14 yielded an average of 0.529 (SD = 0.203), suggesting a complex, potentially multi-individual sample. To account for the low-coverage nature of the sedaDNA, we performed a weighted assessment using only chromosomes 1–12, which provide more robust coverage. This subset yielded an average ratio of 0.367 (SD = 0.0136), a value consistent with a male genetic profile, thereby providing more reliable evidence for the sample's sex sedaDNA origin.

4.4 The Mitochondrial DNA of ElMiron14

We explored the retrieval of mitochondrial DNA from the 101 sequencing attempts of the 9 libraries. After merging all the reads and removing duplicates, we obtained only 418 unique sequences in total. This result indicates that the capture baits used in the experiment did not allow for substantial mtDNA recovery from the shotgun libraries, probably due to an error in the experimental side (Fig. S10).

We then applied a mitochondrial capture procedure following the same methodological approach described in Gelabert et al, 2025, (*22*), capturing with commercial myBaits mtDNA baits. The sequenced libraries are presented in Table S27. All the libraries show short reads and substantial deamination (Fig. S11). The results of the calls are presented in Table S4.

For the mitochondrial DNA (Table S27), we used the same methodological approach described in Gelabert et al, 2025, (*22*), capturing with commercial myBaits mtDNA baits. These libraries

were sequenced in paired-end 150 reaction reads on a Novaseq X platform, Vienna BioCenter Core Facilities (VBCF). The reads were then clipped using cutadapt (version 1.2.1) with a minimum length of 30 and removal of polyA tails. The clipped reads were then collapsed with PEAR 0.9.11(*62*) with a minimum overlap of 11 bases. The resulting reads were aligned against the rCRS mtDNA reference genome using BWA aln(*55*) (Version 0.7.5), with seeding disabled and a gap penalty of 2 set for the open end. Duplicate mapped reads were removed using Picard Tools 2.21.4(*56*) with default parameters. Reads with mapping qualities below 30 were also removed. Unique and filtered reads were analysed with Qualimap-2.2.1 (*57*) to assess whole-genome coverage depth. MapDamage-2.0.9 (*58*) was used to estimate deamination levels and assess the authenticity of the data. The reads were then converted to fastq using Samtools 1.12 (*59*). These sequences were processed with BLASTn 2.16.0 (*60*) using the NCBI whole-nucleotide database (2024-06-07). The results were analysed in MEGAN 6.25.10(*61*) using the following parameters: Native LCA, bitscore 50, minimum identity of 80, and top score 5, grouping the Primate reads. These were aligned again to the human reference genome and processed with Schmutzi 2.0.0 (*63*) to determine the consensus sequence. We finally examined each position with Samtools mpileup and called the most abundant haplogroup with Haplogrep 3.2.1 (*64*). We plotted the deamination and read length distributions.

Haplogrep predicted the mitochondrial haplogroups as follows: 16333iL1 – U4’9 (77% quality), 16333iL4 – U4’9 (82% quality), and 16333iL5 – U2'3'4'7'8'9 (81% quality). These results indicate the presence of multiple individuals, reflecting the diversity of U haplogroups typical of Pleistocene populations. Collectively, the data suggest that multiple individuals, rather than a single individual, contributed to the genetic material analysed.

4.5 Taxonomical classification of shotgun sedaDNA

To further explore faunal identification in sedimentary ancient DNA derived from shotgun data, demultiplexed reads were first processed with Cutadapt 5.2 ((*53*) to remove adapters, reads shorter than 30 bp and poly-A tails; and were subsequently concatenated into a single file. Taxonomic classification was performed using the Quicksand 2.6 pipeline (*65*). To ensure high-confidence taxa identification, we applied the following criteria: a minimum of 100 mapped reads, detection of characteristic deamination patterns at both ends of the alignments, and at least

10% breadth of coverage. Using these filters, nine mammalian families were identified (Table S28).

We compared these shotgun-derived classifications with those obtained from in-solution mtDNA bait capture (*22*). While most families were detected by both methods, Lagopus, Pyrrhocorax, Aquila, and Talpa were identified only in the shotgun data. Conversely, families with lower recovery yields in the mtDNA-capture dataset, such as Felidae and Ursidae, were absent in the shotgun reads. These discrepancies likely reflect differences in the sensitivity of the computational workflows, the bias towards species or genera included in the baits design, and the specific reference databases employed in each pipeline. Notably, there were no major disagreements regarding the primary genera or species detected; discrepancies were largely limited to low-abundance taxa identified exclusively in the capture dataset or shotgun identifications of species not present in the mtDNA capture. Future research should further investigate these differences in taxonomic resolution when applying differential methods to reconstruct past biodiversity

5. Population genetics and ADMIXTOOLS 2

In order to analyse our newly generated data within the scope of the Upper Palaeolithic population genomics, we built a dataset of several hundred individuals (*7–10*, *13*, *36*, *89–101*), from which we collected the genotypes overlapping the 1240k database. This was used for all the population genomics analyses. We employed the methodology described in (*83*) to identify ROH in Ostunib and Isturitz and the rest of the Gravettian and Aurignacian individuals with more than 400,000 SNPs. We used the ROH detected to estimate the effective population sizes of the populations of the studied individuals

We used ADMIXTOOLS 2 to identify the best-fitting admixture graph models that could explain the differences observed in the *f*-statistics for Isturitz (see Methods). Out of 106,013 generated graphs, 96 were retained as plausible models, defined as those within $\Delta = 2$ of the best score (plausible_graphs <- combined %>% filter(score - best_score <= 2)), a commonly used threshold in likelihood-based model comparison that retains alternative models statistically indistinguishable from the best-fitting one. Within these plausible models, Isturitz was placed in

a specific configuration 75 times, while 21 models included an ancestor from Sungir_UP as a source, and the remainder represented topologies not compatible with the current demographic history. It is important to note that Věstonice (Czechia_Gravettian) is almost the same age as Isturitz, and therefore, the signal likely reflects an unsampled ancestor rather than deriving directly from Věstonice. A binomial test confirmed that this placement was significantly more frequent than expected under a null probability of 0.5 (one-sided $p \approx 0.035$), supporting the interpretation that this topology reflects a genuine genetic signal rather than random variation. We repeated the same procedure for other individuals assigned to the Fournol cluster with more than 50,000 SNPs, Ormesson, Catalonia_Gravettian, and Malalmuerzo (Figs. S12-14).

To test for differential genetic affinity between Western Gravettian or Solutrean individuals (with >50,000 SNPs) and populations from the Vestonice cluster, we employed f_4-statistics of the form f_4(Mbuti,W_Gravettian;CE_Gravettian_A,CE_Gravttian_B) We utilized a block-jackknife approach to estimate standard errors and derived Z-scores to assess statistical significance. A threshold of $|Z| > 3$ was used to identify cases where a test individual shares significantly more genetic drift with one reference population over another, indicating departures from the null hypothesis of treeness. CE referees to Central-Eastern Europe and W to Western Europe. The results show no differential affinity of a Western Gravettian or Solutrean individual to CE Gravettians (Fig. S15).

5.1 Neanderthal ancestry determination

Using F4-ratio (*39*), we determined that the Isturitz genome has about 1.9% Neanderthal ancestry, Ostuni1b has about 1.3%, and the mother (Ostuni1) has about 1.5%. These are typical values for the period, and not significantly different from each other (*82*, *102*). No determination was possible for ElMiron14_PMD_9trim or Chufin_PMD_10trim.

5.2 Phenotypic prediction

We used aHISplex (*41*) (https://github.com/zmaroti/aHISplex) to predict the Eye, Hair and Skin colour of the newly reported samples using the published pipeline that involves imputation, despite our samples are below the required threshold of coverage we obtained for all of them

predictions that involve Brown eyes, Black hair, Dark-Black/Dark skin. Which aligns with the previous knowledge of Upper Pleistocene European Populations. Nevertheless, these predictions should be take.

5.4 Admixture time determination

We used the software DATES_v4010 to estimate the gene-flow dates of the detected events. Following the recommendations, we restricted this analysis to populations or individuals with substantial number of SNPs covers (300,000 SNPs). Specifically we tested the presence of an East-West gene-flow in the Western Gravettian individuals (Ormesson, Isturitz,Fournol, Catalonia_Gravettian) using Russia_Sunghir_UP, Russia_Kostenki14_UP, Czechia_Gravettian as possible sources of Eastern ancestry and keeping GoyetQ116 or Ormesson as Western sources. The results are presented in Table S29. Only the models involving Isturitz have a Z-score>3, however the ones involving Ormesson have low SE and show almost the same dates as Isturitz, supporting a gene-flow event between 31,500 and 32,500 cal BP. We also tried combinations using Ostuni1 as a possible admixed individual but we did not reach any successful date, which corroborated the evidence that it belongs to a single genetic ancestry.

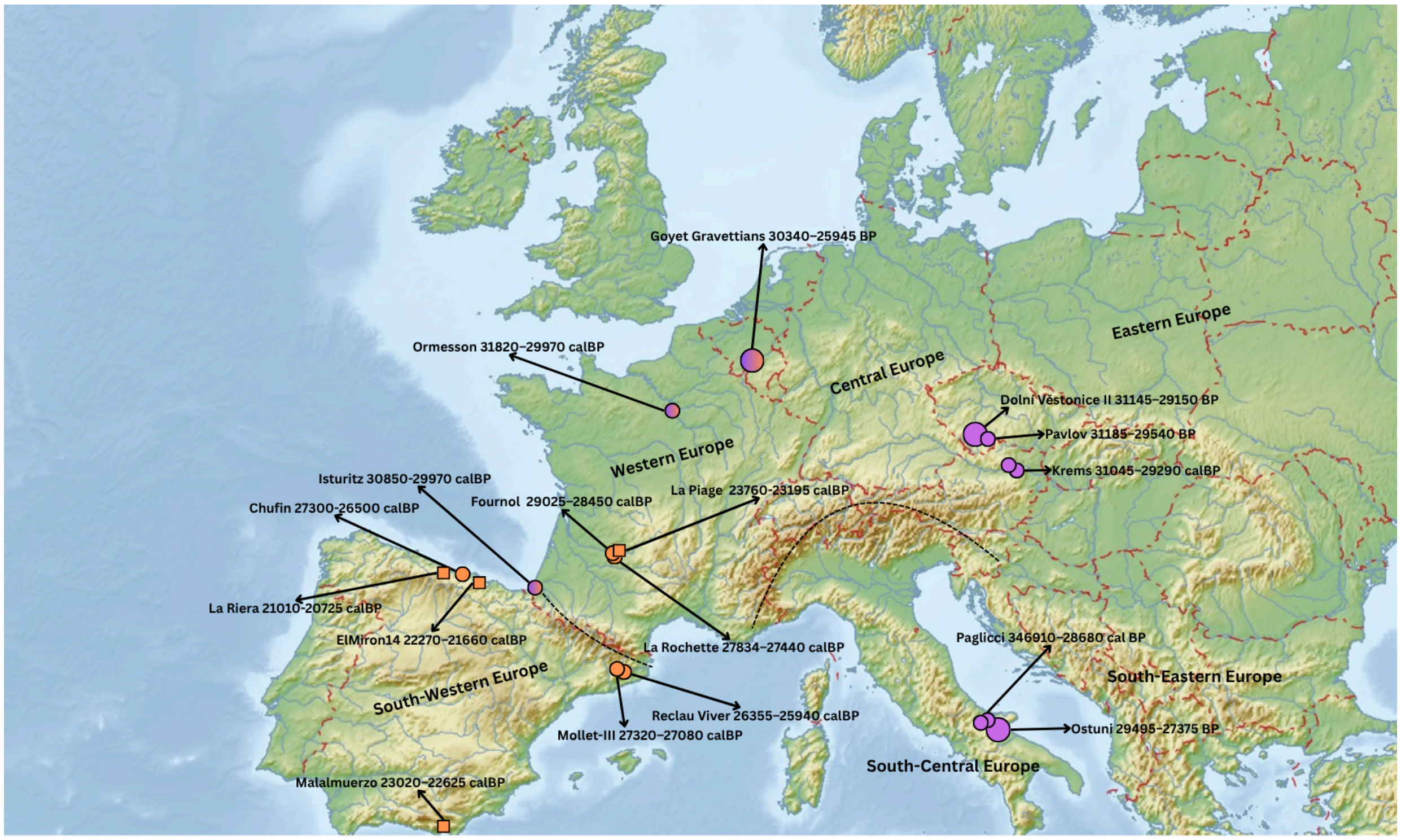

Fig. S1.

Gravettian and Solutrean associated genomic data: Distribution of the available Gravettian and Solutrean genomic data, which spans from 35,000 to 20,000 years bp. The dates refer to direct dates or the range in case of multiple individuals. Orange depicts Fournol-ancestry and Purple-Vestonice ancestry. Noted the case of the admixed individuals in Goyet, Ormesson and Isturitz. Squares represent Solutrean-associated individuals and circles Gravettian-associated ones. The map was obtained from https://mapswire.com/maps/europe-physical-maps/#pid=3 *under a CC-BY 4.0 license* (https://creativecommons.org/licenses/by/4.0/).

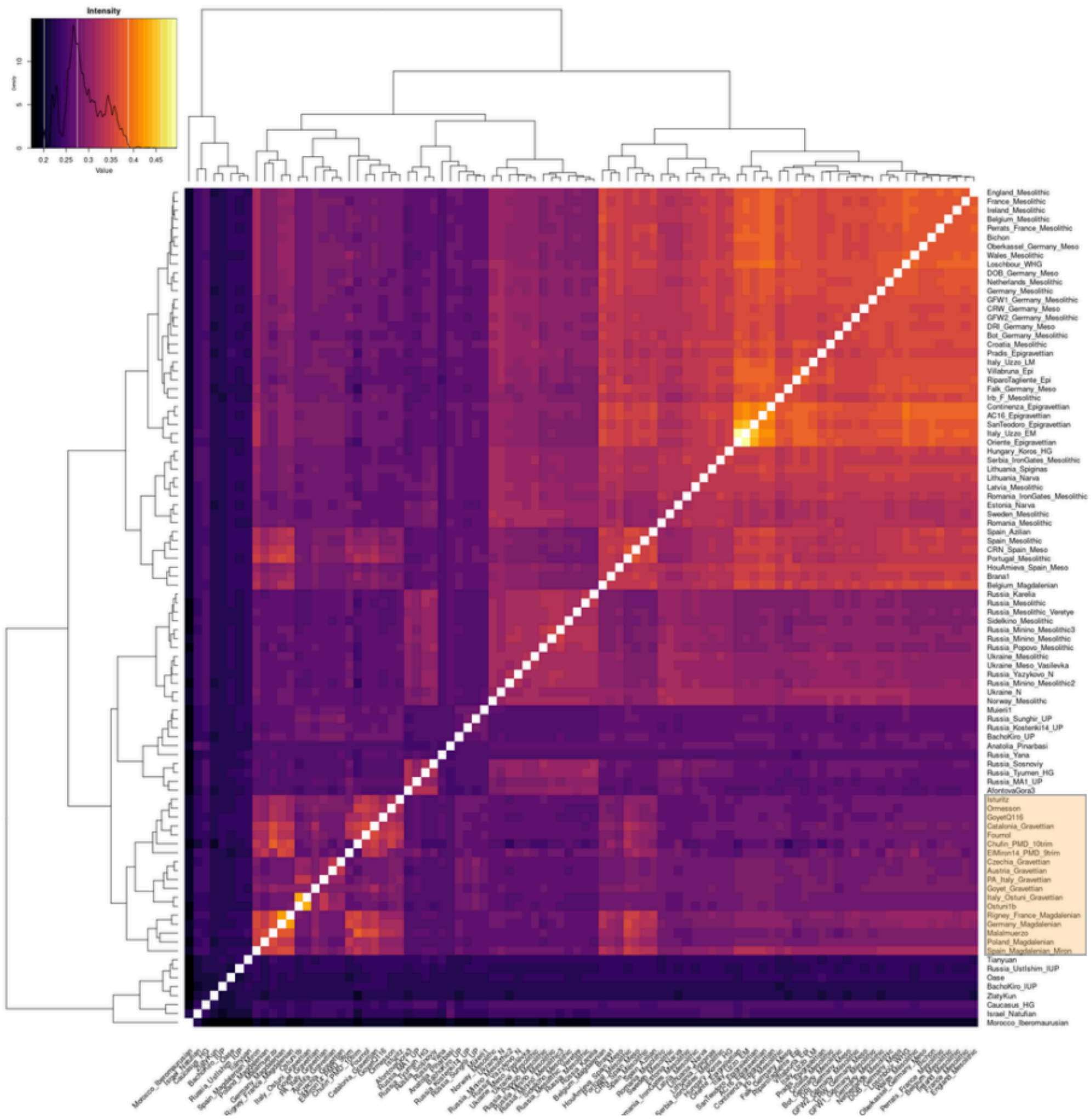

Fig. S2.

***f_3* matrix of Pleistocene and Holocene hunter-gatherers:** f_3 matrix reflecting genetic distances among all possible pairs of individuals, calculated as f_3(HGi, HGj; Mbuti) using samples with more than 10,000 SNPs from Table S5. Samples are grouped according to classifications proposed in previous publications covering Upper Palaeolithic (UP) populations. The newly generated samples, Isturitz, ElMiron14_PMD_9trim, and Chufin_PMD_10trim fall within the Fournol cluster individuals. Ostuni1b is nearly identical to Ostuni. The orange square marks the main genetic cluster that groups the newly generated samples, showing genetic similarity to the pre-LGM European UP individuals.

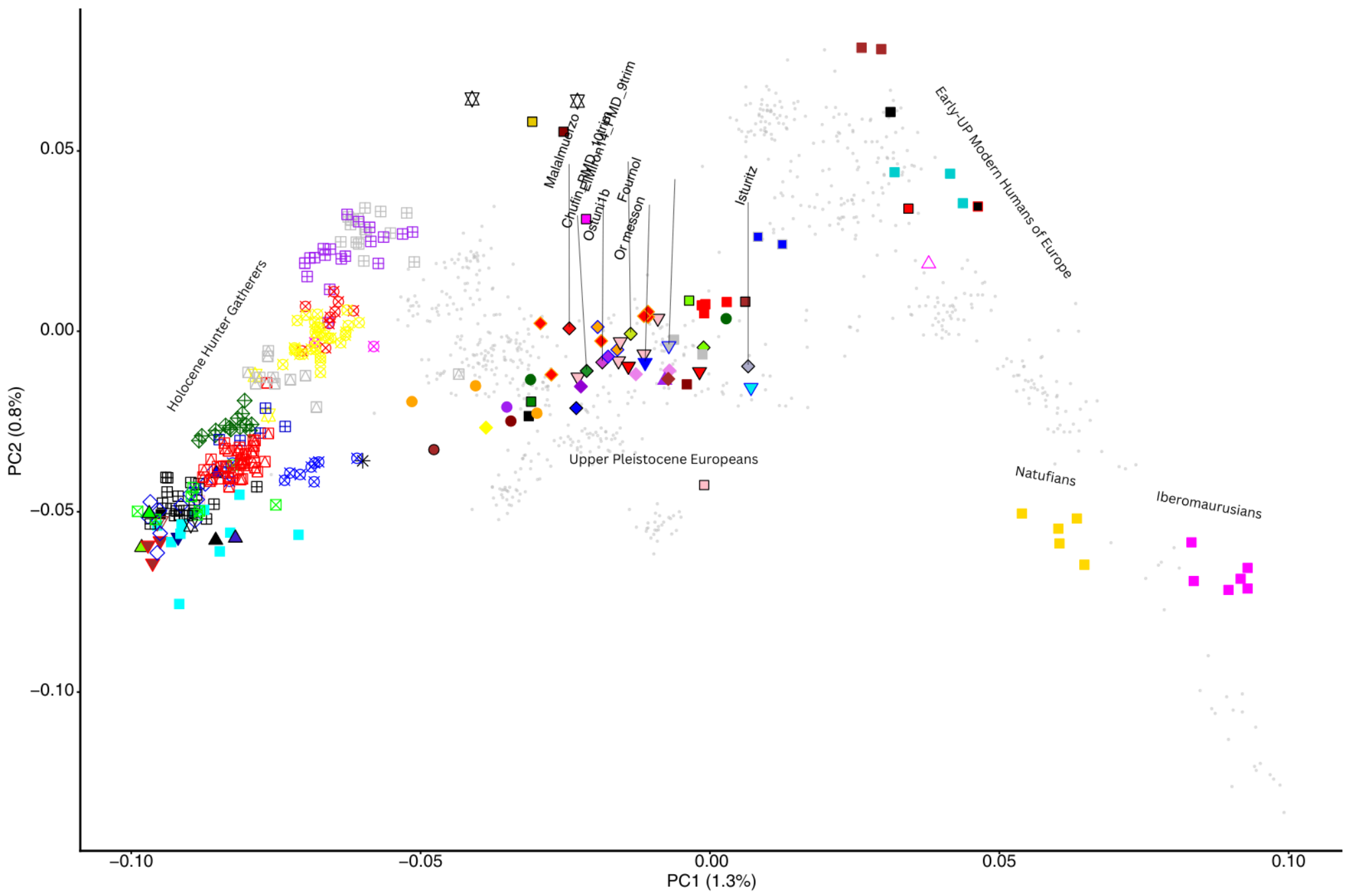

Fig. S3.

Principal component analysis of Pleistocene humans: A Principal Components Analysis (PCA) was performed using modern European genomes as a reference panel, onto which the samples listed in Table S5 were projected. The ancient individuals Isturitz, ElMiron_PMD_9trim, Ostuni1b, and Chufin_PMD_10trim fall within the range of genetic variation observed among Upper Pleistocene European populations, consistent with previously described patterns of genetic diversity for this period.

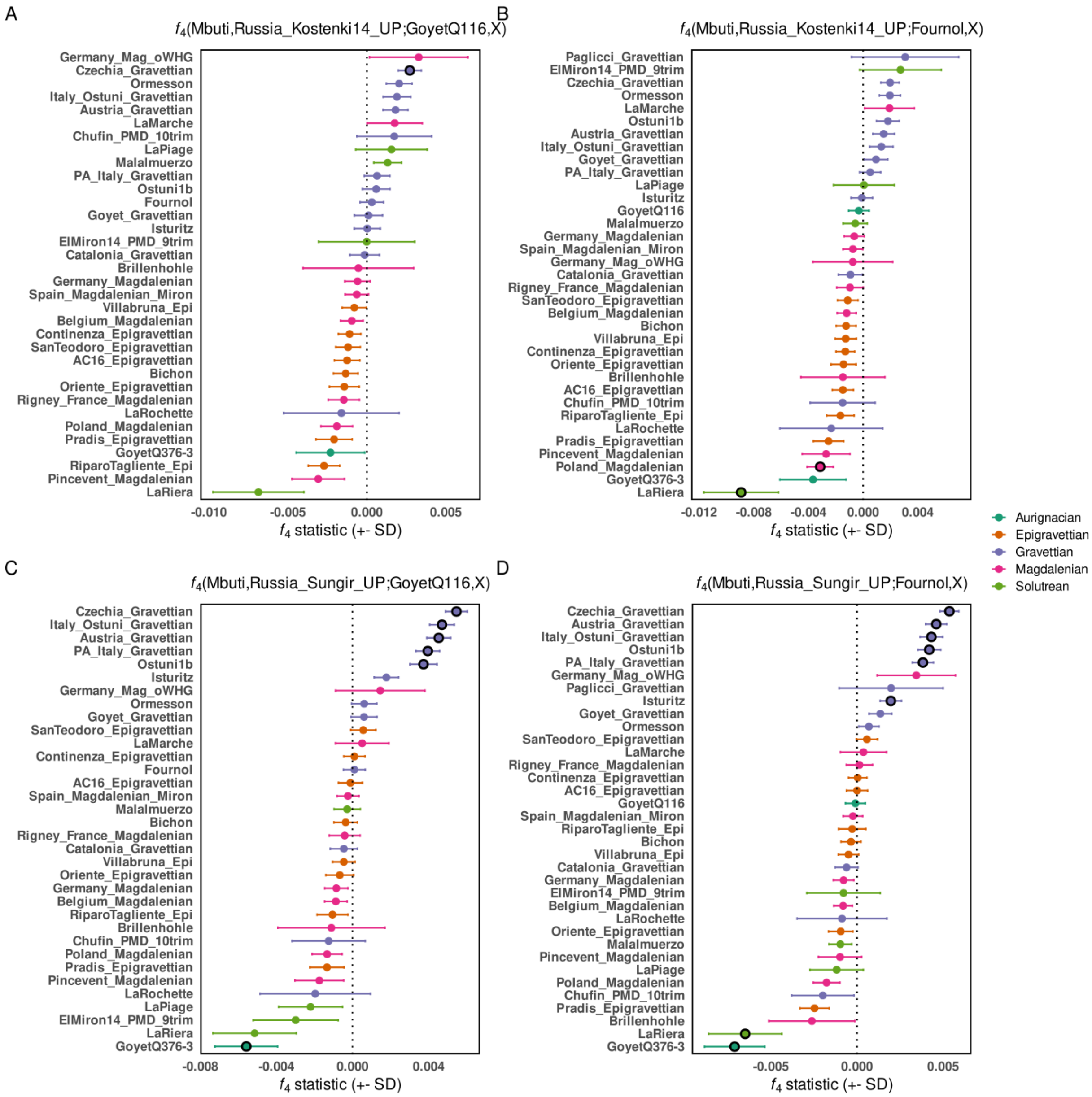

Fig. S4.

Genomic asymmetries among Upper Pleistocene European populations relative to Russia_Sungir_UP and Russia_Kostenki14_UP: The plots show that Russia_Kostenki14_UP displays greater affinity to Czechia_Gravettian when compared with GoyetQ116 (A,B). Comparisons involving Russia_Sungir_UP ancestry relative to GoyetQ116 and Fournol highlight the differential genetic affinities among Gravettian populations across Europe (C,D). The genome of Isturitz is the only genome from southwestern Europe to which Russia_Sungir_UP

shows more affinity in comparison to Fournol (B). Empty circles indicate results that are not statistically significant ($-3 < Z < 3$), and colours represent cultural attribution. Error bars correspond to one standard error.

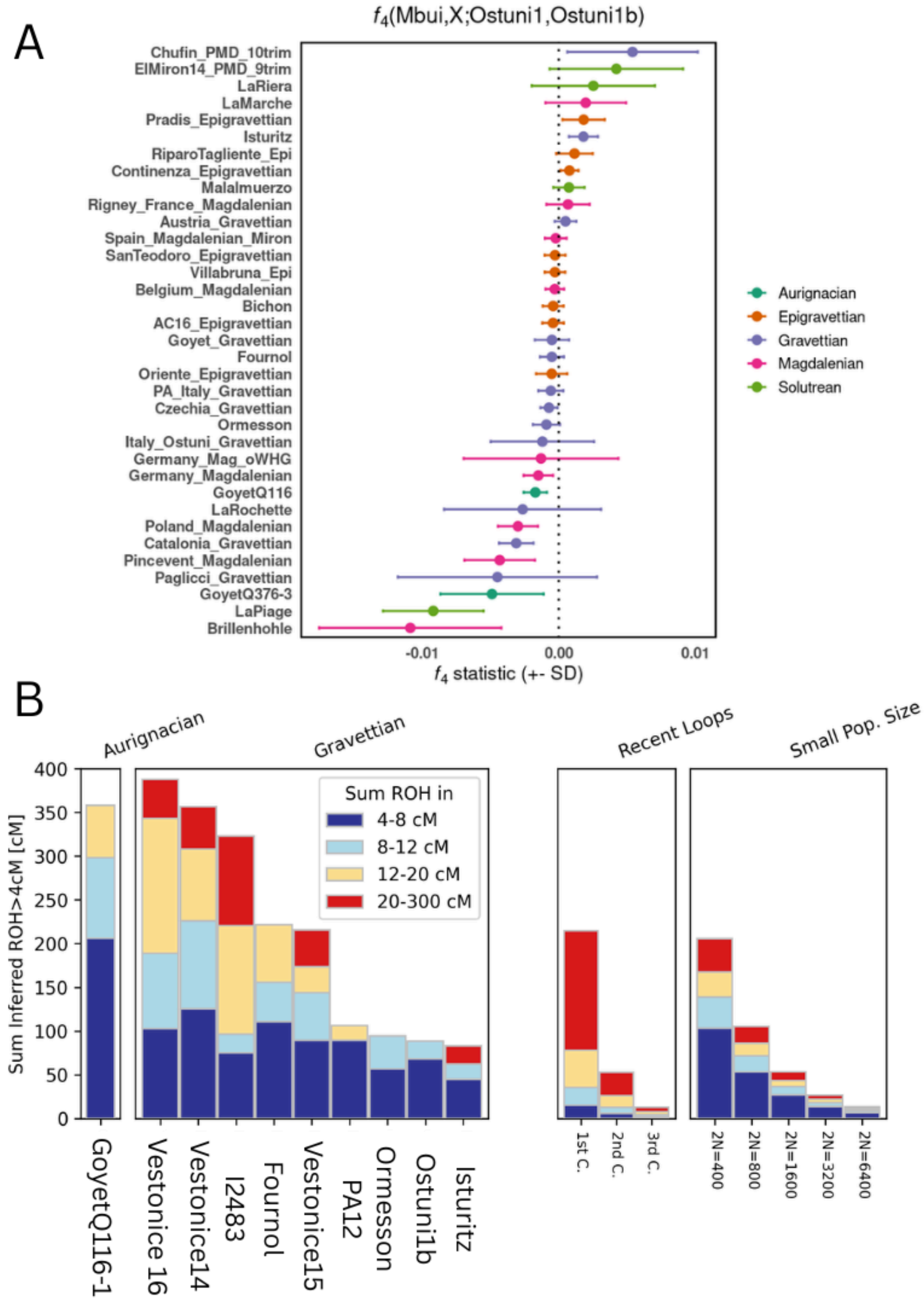

Fig. S5.

Ostuni1 b genome characteristics and ROH of Gravettian-Aurignacian related individuals: A) Comparison of Ostuni1b and Ostuni1 in the form f_4(Mbuti, X; Ostuni1, Ostuni1b). Individuals on the positive side of the plot show a greater affinity to Ostuni1b, while those on the negative side to Ostuni1, no values are significant. B) ROH detected in the newly reported and published Aurignacian and Gravettian data from Central and Western Europe.

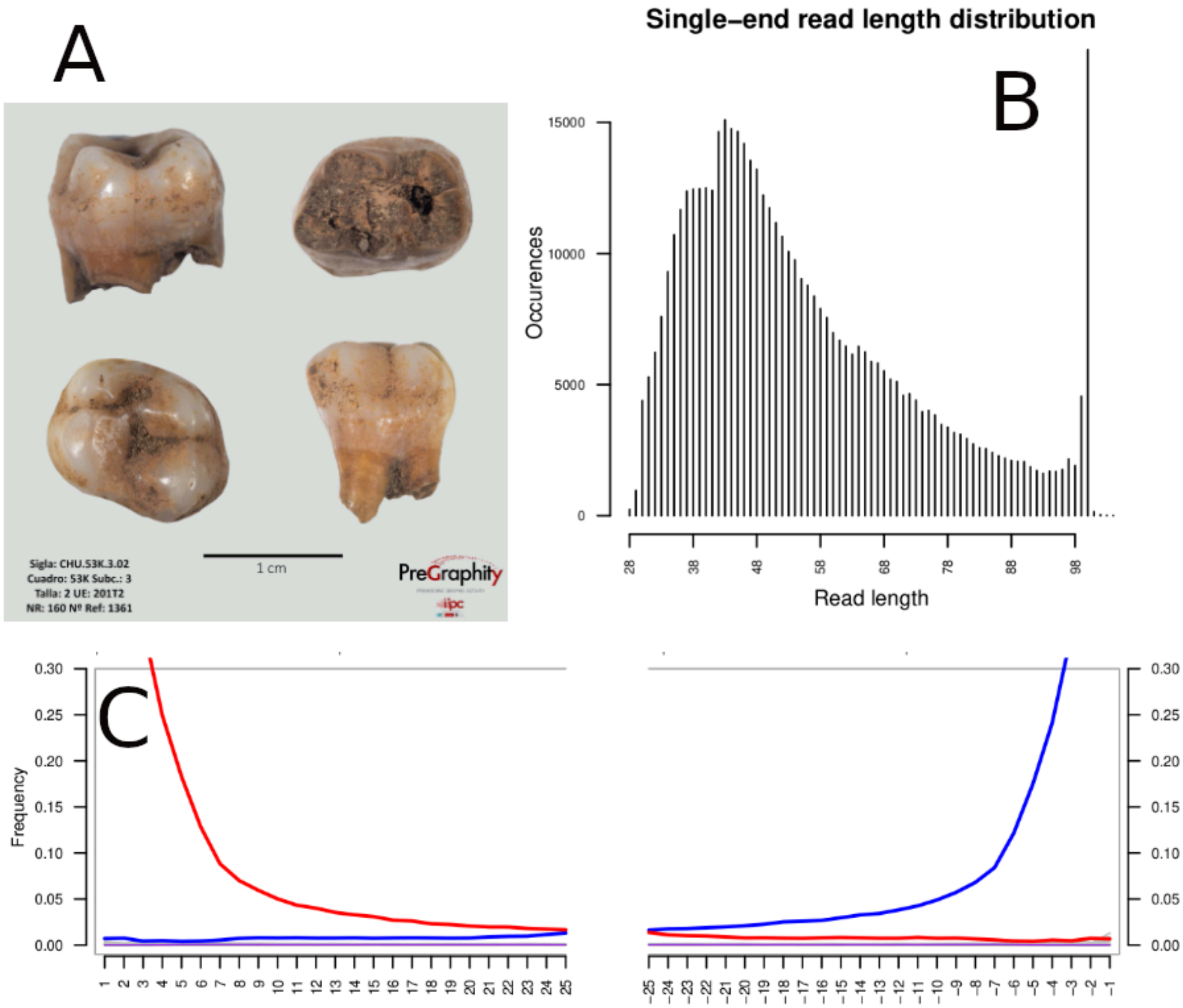

Fig. S6.

Chufín Tooth analyses: A) Photograph of the Chufín tooth specimen; B) Distribution of read lengths from PMD3-filtered reads, showing characteristic fragmentation patterns typical of degraded ancient DNA; C) Cytosine deamination patterns along sequencing reads indicate ancient DNA authenticity, with the top plot showing elevated C-to-T transition rates at the 5' ends.

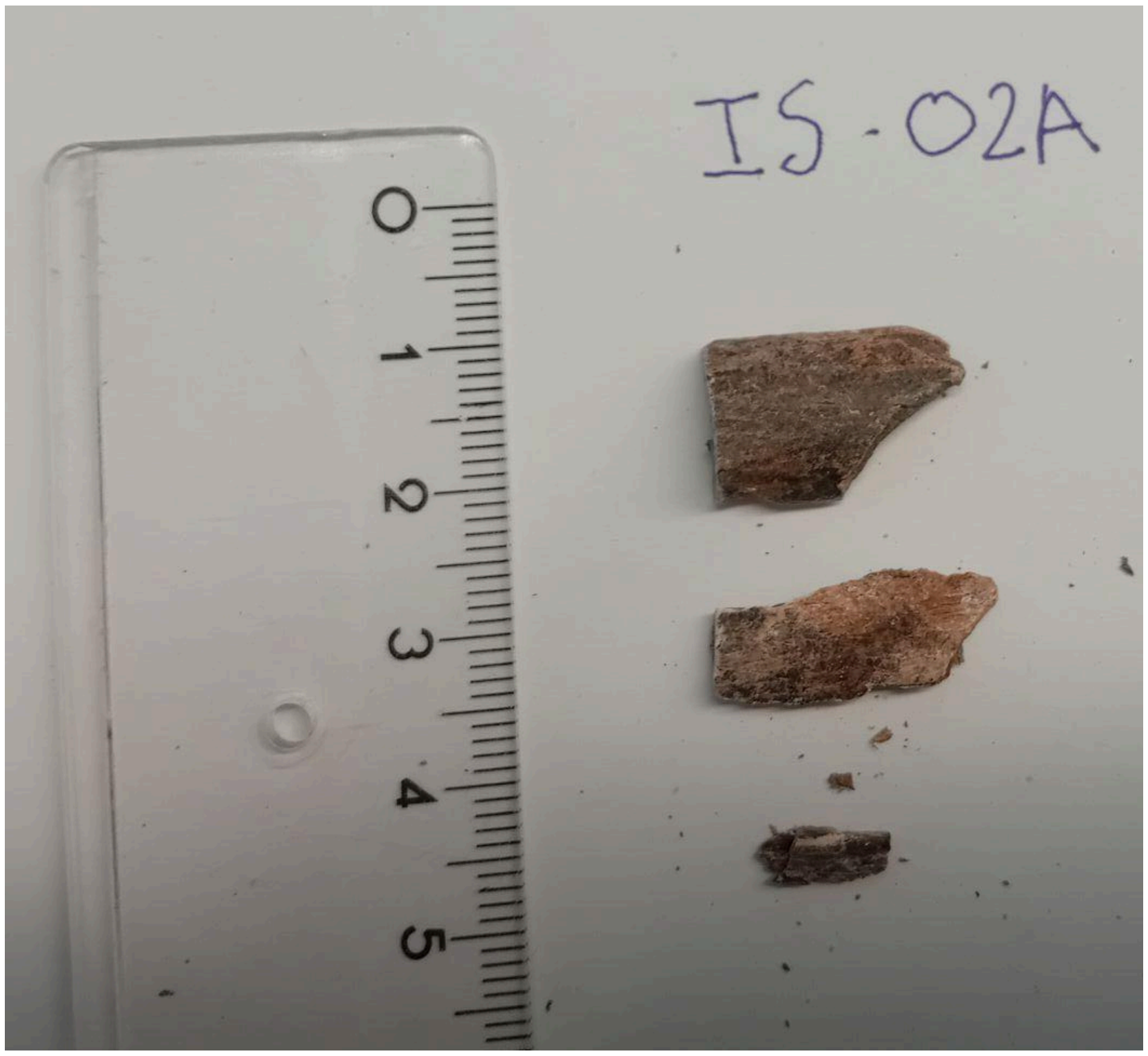

Fig. S7.

IS-02A bone sampled in this study.

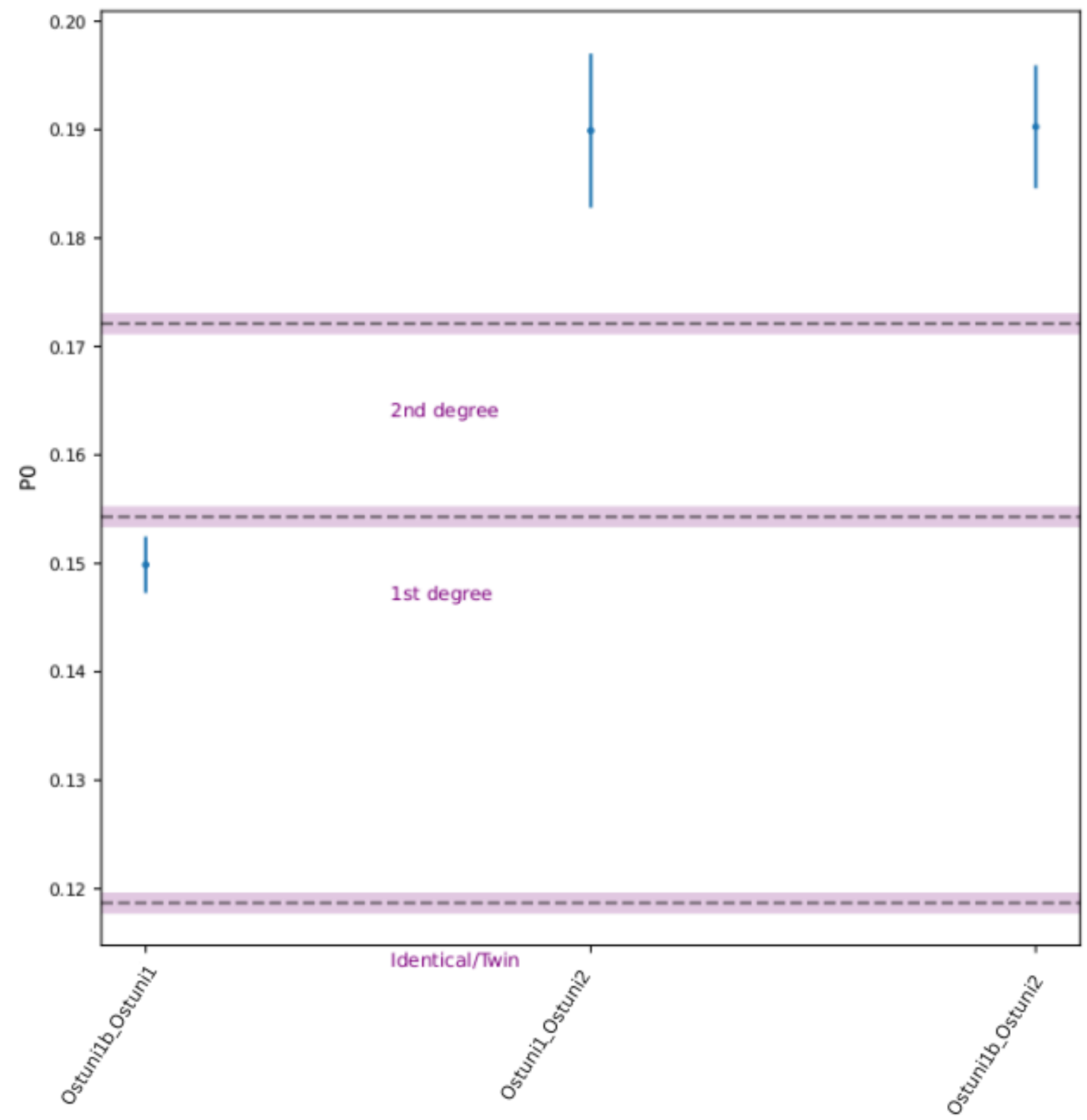

Fig. S8.

Ostuni1-Ostuni1b Kinship: READv2 results of the comparison of Ostuni 1 and Ostuni1b. Ostuni 1b-Ostuni1 are compatible with 1st degree relatives.

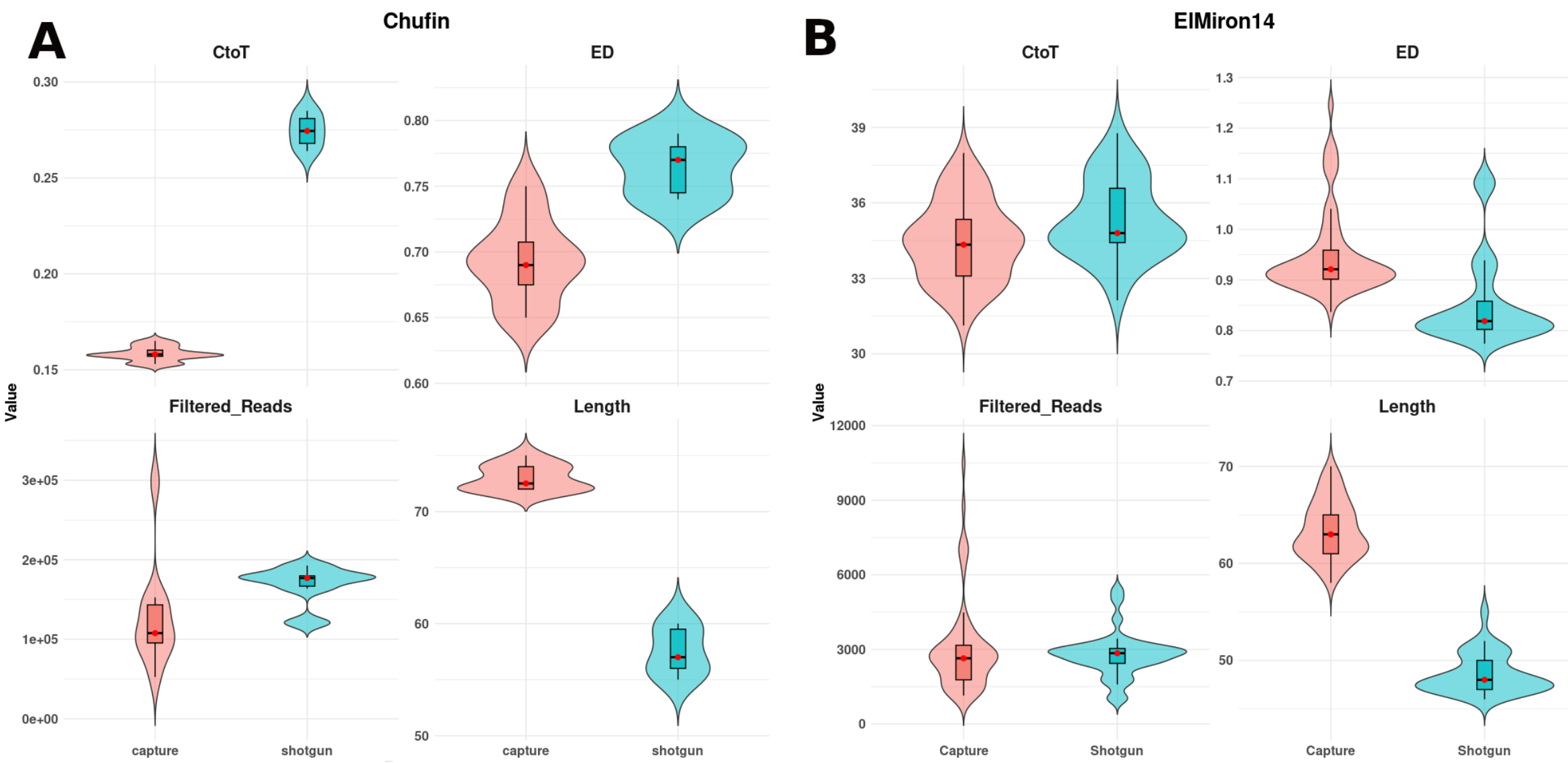

Fig. S9.

Distribution of key metrics used to characterise the ElMiron14 sample (A) and Chufín sample (B). The edit distance (ED), deamination frequency at the 5′ end, recovery of reads, and read length are shown. Red denotes shotgun data, and blue denotes capture data.

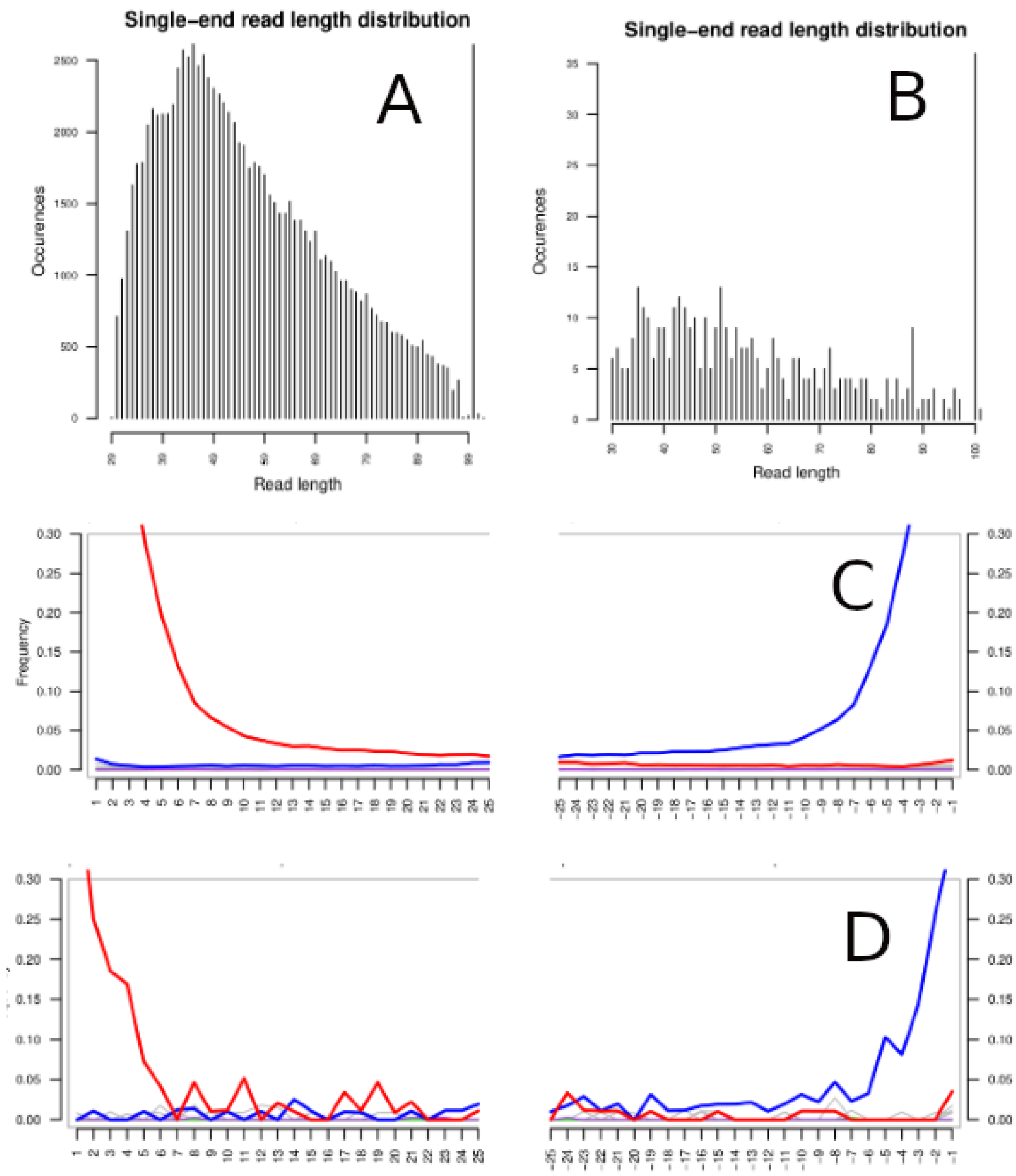

Fig. S10.

ElMiron14 sequencing: A) Distribution of read lengths of ElMiron14_pmd, showing characteristic fragmentation patterns typical of degraded ancient DNA, B) Distribution of read lengths of ElMiron14 mtDNA, showing characteristic fragmentation patterns typical of degraded ancient DNA, C) Cytosine deamination patterns along sequencing reads of ElMiron14_PMD, indicative of ancient DNA authenticity. D) Cytosine deamination patterns along sequencing reads of ElMiron14 mtDNA, indicative of ancient DNA authenticity.

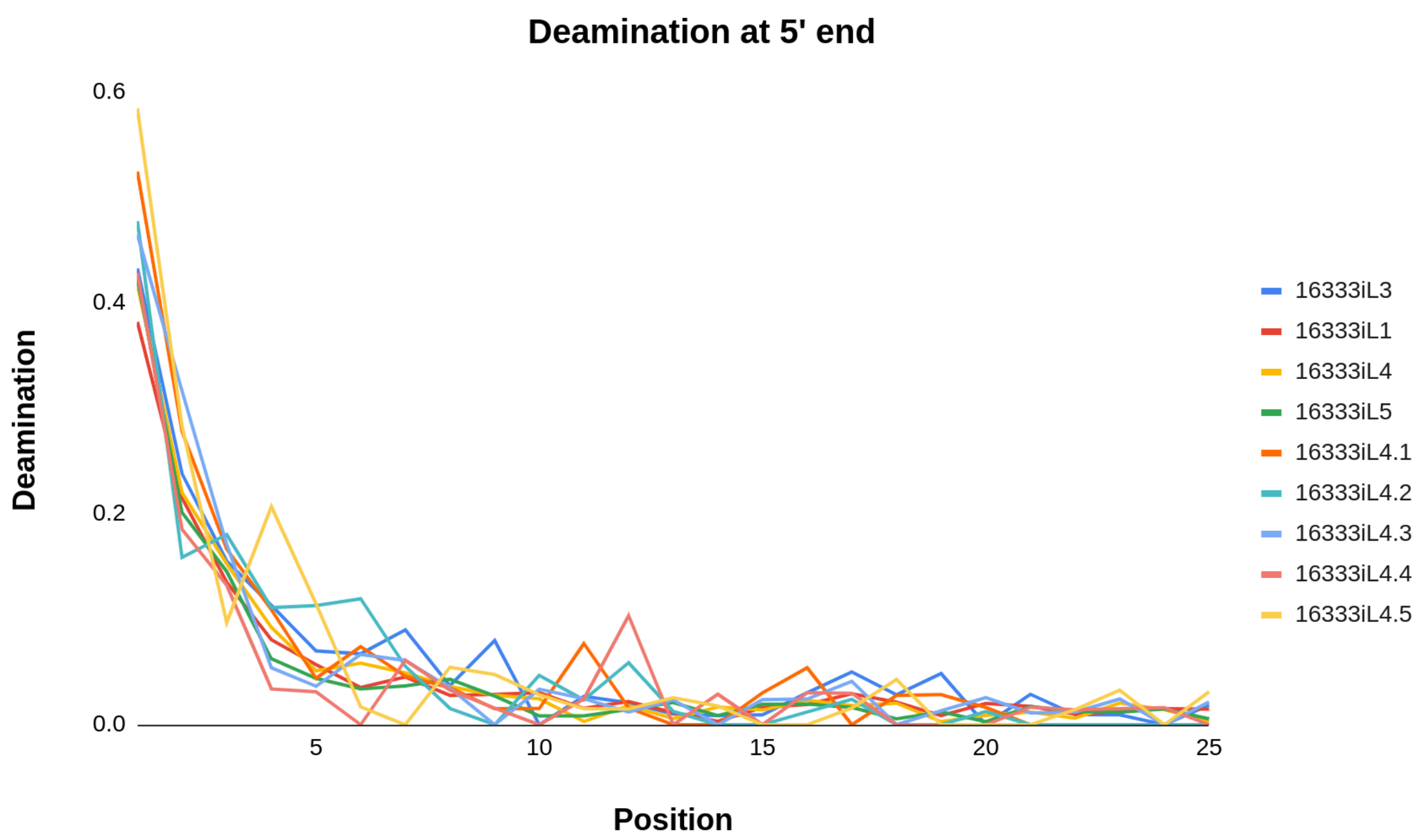

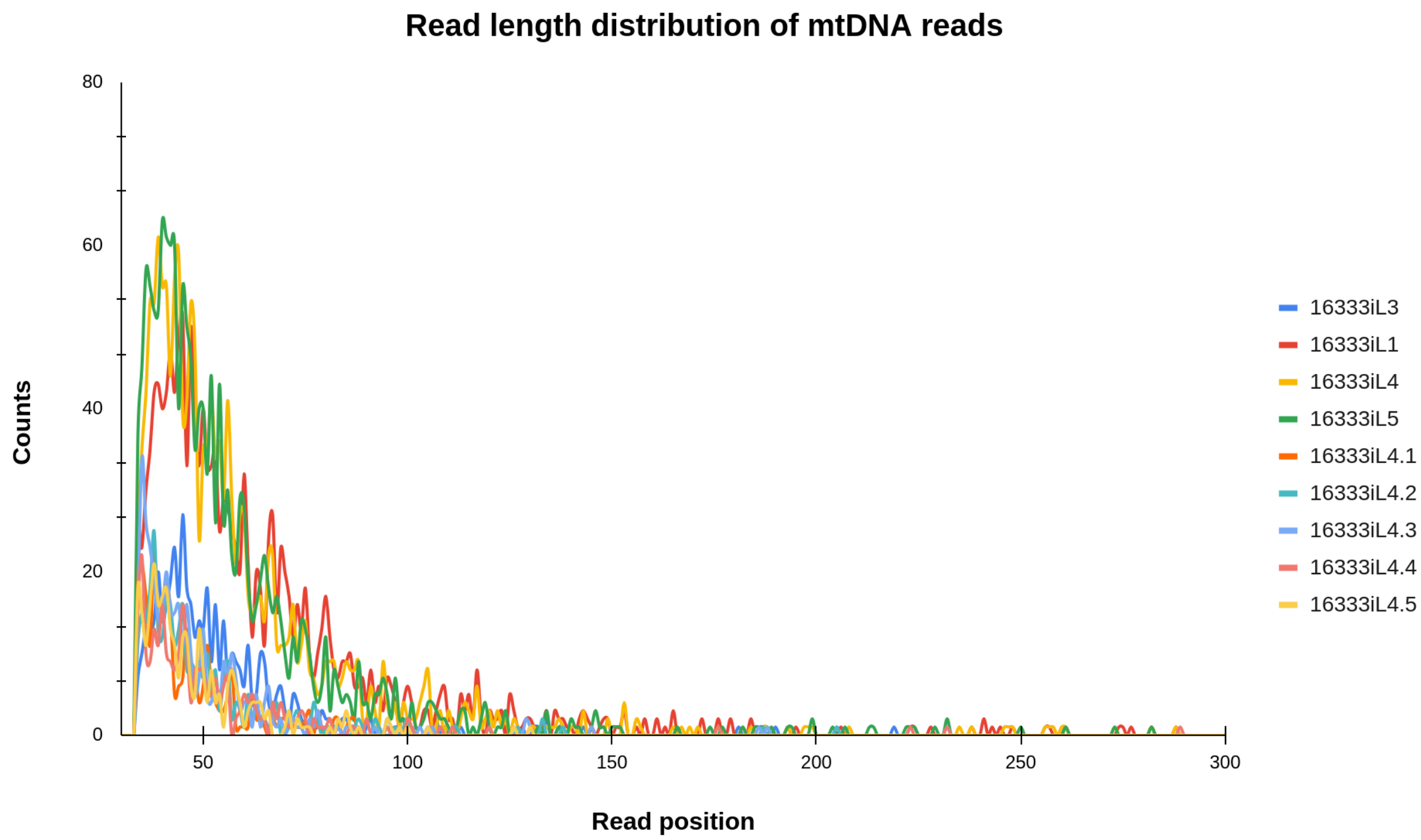

Fig. S11.

Mitochondrial DNA data from ElMiron14: top: Mitochondrial DNA reads of the ElMiron14 samples, the sequenced libraries show deamination and short reads (bottom).

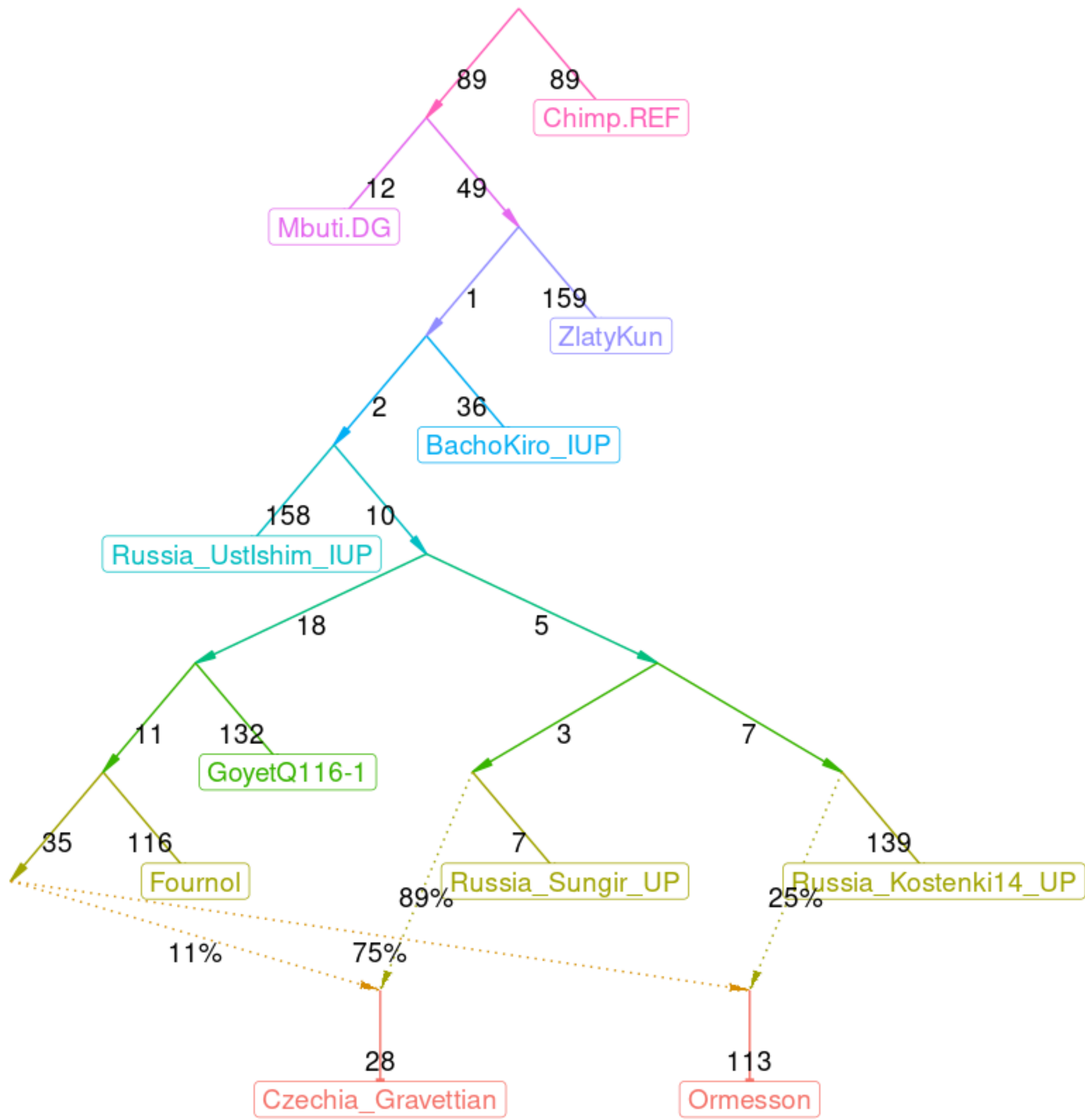

Fig. S12.

Admixture graph of Ormesson individual: Admixture graph fitted with the find_graphs function of ADMIXTOOLS 2, exploring models that could explain the quantitative values of the f-statistics relating the individuals. Across the 44 plausible graphs generated with these populations, Ormesson was consistently placed as an admixture of Fournol and preferably Russia_Kostirnki14_UP in all of these.

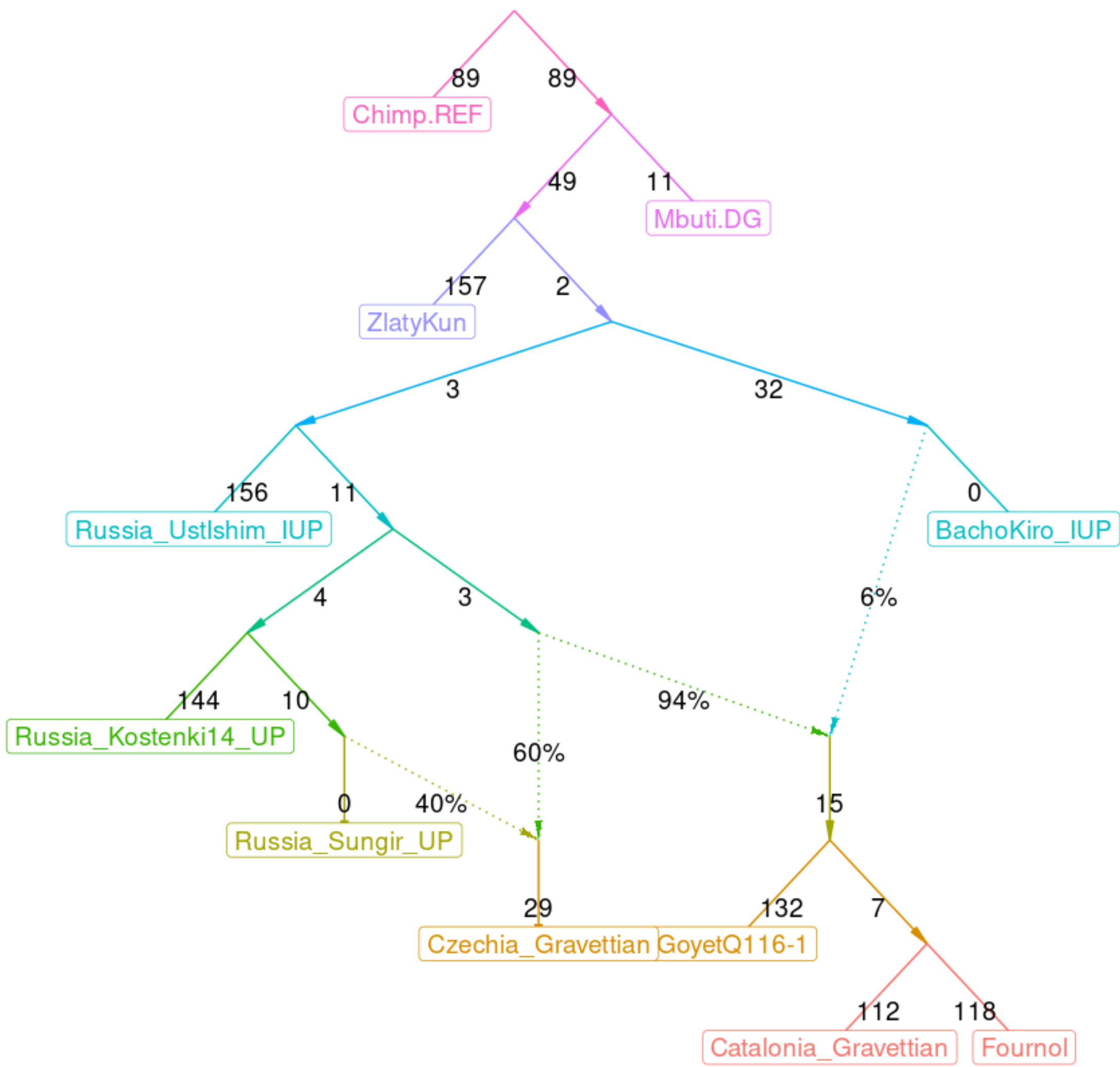

Fig. S13.

Admixture graph of Catalonia_Gravettian individuals: Admixture graph fitted with the find_graphs function of ADMIXTOOLS 2, exploring models that could explain the quantitative values of the f-statistics relating the individuals. Across the 101 plausible graphs generated with these populations, Catalonia_Gravettian was consistently placed as a sister clade of Fournol in all of these.

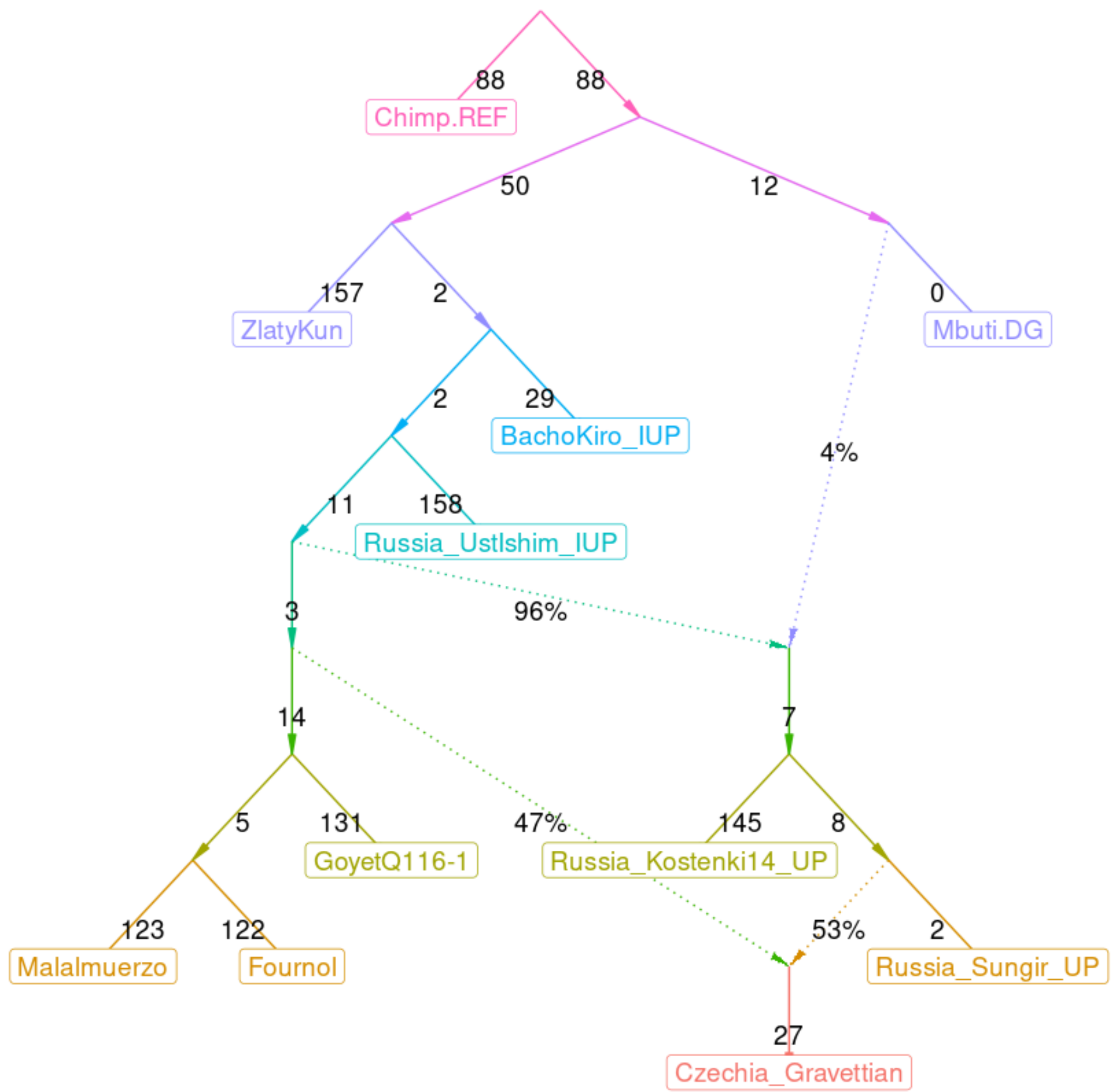

Fig. S14.

Admixture graph of Malalmuerzo individual: Admixture graph fitted with the find_graphs function of ADMIXTOOLS 2, exploring models that could explain the quantitative values of the f-statistics relating the individuals. Across the 159 plausible graphs generated with these populations, Malalmuerzo was consistently placed as a sister clade of Fournol in all of these.

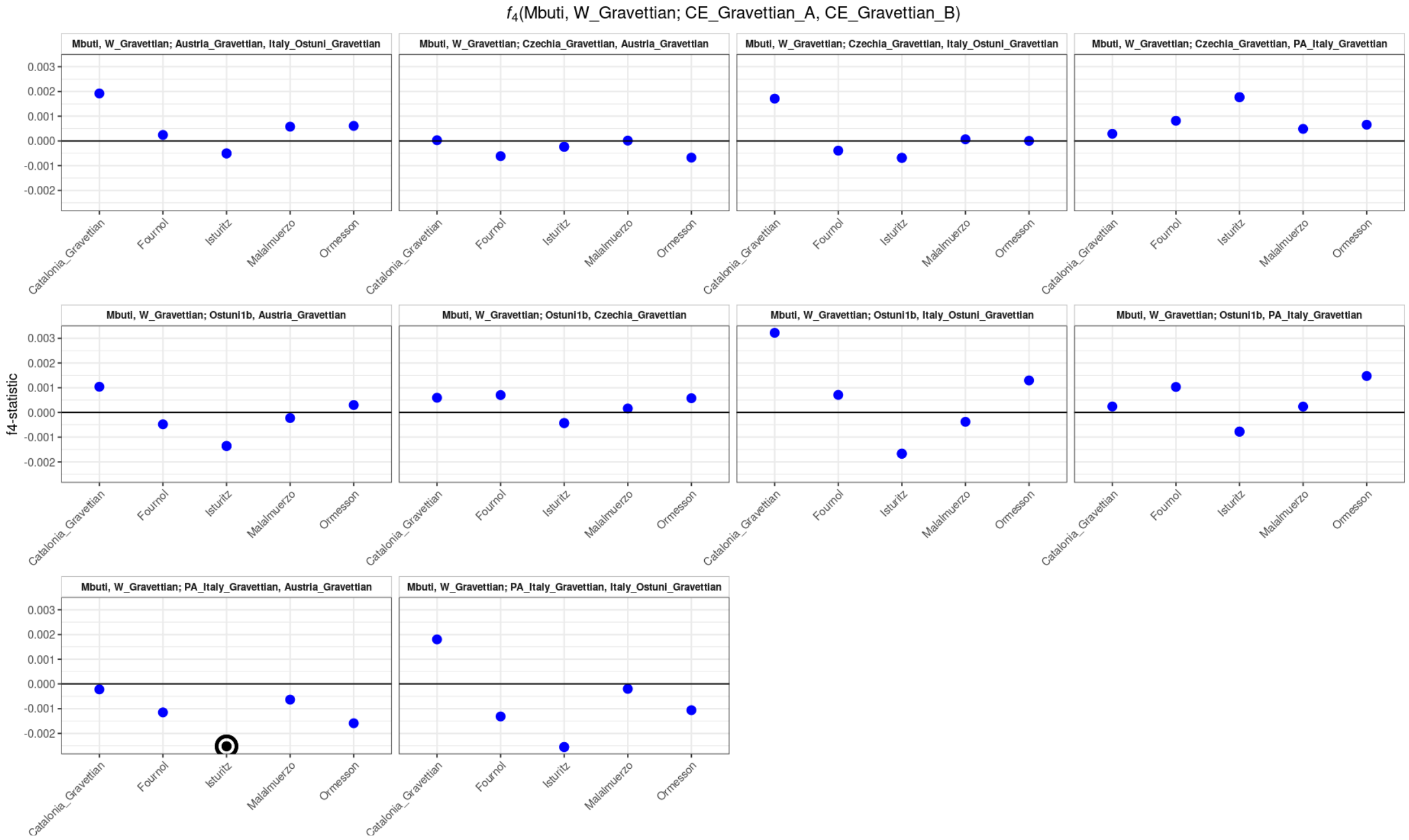

Fig. S15.

Relationship between Fournol-like Gravettians and Vestonice-like ones: f_4-statistics results regarding the comparative affinity of Western Gravettian or Solutrean individuals (with > 50,000 SNPs) in comparison to Eastern and Central Gravettian individuals from the Vestonice cluster. We detect that Isturitz shows a higher statistical affinity to the PA12 individual (PA_Italy_Gravettian) vs. the Austrian Gravettians from Krems (Z = -3.042). In general, the individuals from the West do not appear to share significant differences in comparison to the Eastern and Central. CE referees to Central and Eastern Gravettian individuals and W to Western Gravettian or Solutrean individuals. The black circle means statistical significance.

Table S9.

Library	Point est.	Down	Up
l2517	0.02	0.01	0.03
l2518	0.02	0.01	0.03
l2879	0.02	0.01	0.03
l1812	0.03	0.01	0.05

Table S10.

Library	Positions	Majority	Minority	Point est.	Lower	Upper
l2517	4	1240	59	0.045	0.034	0.056
l2518	4	1177	81	0.064	0.05	0.078
l2879	4	613	37	0.057	0.039	0.075
l1812	0	0	0	NA	NA	NA

Table S11.

Library	Point est.	Lower	Upper
l2517	0.03	0.03	0.04
l2518	0.04	0.03	0.05
l2879	0.07	0.05	0.09
l1812	0.33	0.07	0.93

Table S12.

Method	Mean	Median	SD	Min	Max
Capture	128042	107684	67117	52450	298066
Shotgun	168576	176984	25141	120857	192634

Table S13.

Computed statistics on the Edit distance per Extract ID of ElMiron14.

**ExtractID**	**mean**	**median**	**sd**	**min**	**max**
1987	0.926	0.924	0.109	0.774	1.17
2668	0.916	0.906	0.0928	0.784	1.25

Table S14.

Computed statistics on the Edit distance per Library ID of ElMiron14.

**LibraryID**	**mean**	**median**	**sd**	**min**	**max**
l1980	1.00	0.949	0.147	0.888	1.17
l2236	1.09	1.09	0.0477	1.02	1.16
l2588	0.930	0.932	0.0502	0.837	1.00
l2589	0.873	0.891	0.0499	0.784	0.929
l2590	0.899	0.888	0.117	0.795	1.25
l2591	0.883	0.912	0.0648	0.790	0.954
l2592	0.885	0.905	0.0505	0.798	0.934
l2593	0.875	0.884	0.0426	0.802	0.957
l2594	0.908	0.922	0.0968	0.774	1.11

Table S15.

Computed statistics on the read length per Extract ID of ElMiron14.

ExtractID	mean	median	sd	min	max
1987	61.8	65	7.68	50	70
2668	57.9	61	7.36	46	69

Table S16.

Computed statistics on the read length per Library ID of ElMiron14.

LibraryID	mean	median	sd	min	max
l1980	64.7	69	8.39	55	70
l2236	60.5	62	6.52	51	69
l2588	56.7	60	7.33	47	65
l2589	58.2	61.5	7.62	48	66
l2590	57.6	60	7.95	47	67
l2591	56.4	59.5	7.29	46	64
l2592	58.6	62	8.31	47	68
l2593	57.9	61	7.50	47	65
l2594	61.1	64.5	7.72	50	69

Table S17.

Computed statistics on the deamination at 5’ per extract ID of ElMiron14.

**ExtractID**	**mean**	**median**	**sd**	**min**	**max**
1987	0.412	0.389	0.0453	0.356	0.482
2668	0.410	0.389	0.0574	0.298	0.532

Table S18.

Computed statistics on deamination at 5’ per Library ID of ElMiron14.

**LibraryID**	**mean**	**median**	**sd**	**min**	**max**
l1980	0.405	0.387	0.0597	0.356	0.471
l2236	0.401	0.388	0.0506	0.310	0.472
l2588	0.421	0.389	0.0622	0.358	0.532
l2589	0.392	0.368	0.0641	0.298	0.503
l2590	0.408	0.380	0.0577	0.344	0.519
l2591	0.437	0.422	0.0484	0.381	0.516
l2592	0.411	0.384	0.0613	0.347	0.507
l2593	0.403	0.382	0.0582	0.330	0.505
l2594	0.414	0.394	0.0442	0.366	0.482

Table S19.

Computed statistics on human reads recovery per extract ID.

ExtractID	mean	median	sd	min	max
1987	5294	5000	2154	2870	10480
2668	2523	2612	1015	993	8757

Table S20

Computed statistics on human reads recovery per library ID of ElMiron14.

LibraryID	mean	median	sd	min	max
l1980	7599	6930	2612	5387	10480
l2236	3065	2557	2104	1063	8757
l2588	2334	2487	626	1147	3145
l2589	2344	2622	634	1183	3084
l2590	2450	2500	791	1178	3918
l2591	2550	2623	910	993	4090
l2592	2554	2820	590	1573	3115
l2593	2427	2628	853	1217	4213
l2594	4717	3802	1689	2870	7243

Table S21.

Computed statistics on human reads recovery per sequencing strategy of ElMiron14.

**Method**	**mean_ed**	**median_ed**	**sd_ed**	**min_ed**	**max_ed**
Capture	0.950	0.922	0.0830	0.837	1.25
Shotgun	0.855	0.819	0.0883	0.774	1.10

Table S22.

Computed statistics on human reads length per sequencing strategy of ElMiron14.

**Method**	**mean**	**median**	**sd**	**min**	**max**
Capture	63.4	63	2.92	58	70
Shotgun	48.6	48	2.02	46	55

Table S23.

Computed statistics on human read deamination per sequencing strategy of ElMiron14.

**Method**	**mean**	**median**	**sd**	**min**	**max**
Capture	0.376	0.379	0.0260	0.298	0.442
Shotgun	0.481	0.476	0.0221	0.449	0.532

Table S24.

Computed statistics on human read recovery per sequencing strategy of ElMiron14.

Method	mean	median	sd	min	max
Capture	2976	2636	1852	214	10480
Shotgun	2779	2846	902	993	5387

Table S25.

Results from the PileupCaller on the four samples generating merging ElMiron14 reads in the four combinations presented.

SampleName	TotalSites	Covered calls
Miron14	1233013	49212
Miron14_S	1233013	1515
Miron14_C	1233013	48384
Miron14_90	1233013	42214

Table S26.

Results of the *f3*-statistics computed on ElMiron14 samples and other contemporary and regional genomes.

Pop1	Pop2	Pop3	Value	SD	SNPs
ElMiron14	ElMiron14_C	Mbuti	0.786272	0.007524	30795
ElMiron14	ElMiron14_90	Mbuti	0.783615	0.008001	26803
ElMiron14	ElMiron14_S	Mbuti	0.733932	0.034405	942
ElMiron14_C	ElMiron14_90	Mbuti	0.785554	0.008061	26318
ElMiron14_90	ElMiron14_S	Mbuti	0.714201	0.035299	866
ElMiron14_C	ElMiron14_S	Mbuti	0.740460	0.054262	438
Href.REF	ElMiron14	Mbuti	0.236326	0.006225	32954
Href.REF	ElMiron14_C	Mbuti	0.235931	0.006247	32426
Href.REF	ElMiron14_S	Mbuti	0.248459	0.029919	985
Href.REF	ElMiron14_90	Mbuti	0.241878	0.006726	28213
Href.REF	Isturitz	Mbuti	0.193309	0.003867	153538
Href.REF	GoyetQ116	Mbuti	0.190310	0.003449	411468
Href.REF	GoyetQ2	Mbuti	0.199044	0.003611	293798
Href.REF	Girona_Grav	Mbuti	0.241513	0.004110	136327
Href.REF	ElMiron_Mag	Mbuti	0.205492	0.003555	434089
Href.REF	Fournol	Mbuti	0.212010	0.003654	408064
Href.REF	Malalmuerzo	Mbuti	0.215603	0.004039	151253
Spanish.DG	Isturitz	Mbuti	0.249433	0.003267	165272

Spanish.DG	GoyetQ116	Mbuti	0.245307	0.002884	442897
Spanish.DG	GoyetQ2	Mbuti	0.256118	0.003026	315798
Spanish.DG	Girona_Grav	Mbuti	0.247653	0.003308	148083
Spanish.DG	ElMiron_Mag	Mbuti	0.257933	0.003003	465486
Spanish.DG	Malalmuerzo	Mbuti	0.251052	0.003393	163038
Spanish.DG	Fournol	Mbuti	0.247220	0.003028	441054
Spanish.DG	ElMiron14	Mbuti	0.253243	0.004752	35711
Spanish.DG	ElMiron14_C	Mbuti	0.253971	0.004791	35125
Spanish.DG	ElMiron14_S	Mbuti	0.237300	0.021585	1081
Spanish.DG	ElMiron14_90	Mbuti	0.251281	0.00504	30606

Table S27.

mtDNA sampling attempt to retrieve human mtDNA from the ElMiron14 sample

Sample	Sequenced reads	Mapped reads	Unique reads	% endo.	Q30 Reads	Dupli. rate	Cov.	C>T 5’	G>A 3’
16333iL3	1556751	6636	1230	0.00	889	1.38	1.6	40.20	36.18
16333iL1	1815085	6938	2278	0.07	1926	1.18	6.02	37.71	37.66
16333iL4	1863438	15663	2517	0.12	2061	1.22	5.63	41.10	42.30
16333iL5	3676163	18377	2826	0.07	2237	1.26	5.4	38.19	41.41
16333iL4.1	3650823	31551	1344	0.08	841	1.60	0.8	40.71	42.85
16333iL4.2	1156483	11663	1224	0.12	781	1.57	1.16	35.88	34.96
16333iL4.3	2862850	30896	1514	0.04	955	1.59	1.02	36.41	32.98
16333iL4.4	3021893	12014	1219	0.05	771	1.58	0.90	35.41	37.16
16333iL4.5	1302657	8932	1161	0.09	749	1.55	0.92	50.36	36.00

Table S28.

Summary of classified taxa using QuickSand v.2.5 of ElMiron14. The reported values correspond to extracted reads, k-mer counts, k-mer duplication rate (DupRate), mapped reads, mean read length, and breadth of coverage, we present the results by family.

Taxa	Extracted	Kmers	Kmers DupRate	Mapped	Deduped	Mean Length	Breadth
Rupicapra	20015	23274	8.44	13652	9800	47.5	0.994
Cervus	13353	11917	11.8	7528	5698	49.2	0.95
Canis	6740	20047	3.71	4344	3697	47.0	0.966
Aquila	4779	6386	4.29	1081	870	45.5	0.643
Talpa	1579	4999	1.67	456	430	46.6	0.653
Microtus	4352	5959	1.99	252	232	46.7	0.378
Lagopus	2435	3423	2.35	216	198	45.0	0.388
Homo	437	2813	1.33	200	182	50.9	0.417
Pyrrhocorax	1139	3021	1.71	140	129	44.6	0.273

Table S29.

Dates admixture estimates obtained through running DATES_v4010, the time range has been calculated from the midpoint of the calibrated date range. The * denotes statistically significant results. The - sign means not feasible.

Pop1	Pop2	Target	Generations	CI	Z-score	Time range
Czechia_Gravettian	GoyetQ116	Isturitz	57.865	14.9	3.8*	31600-32450 BP
Czechia_Gravettian	Ormesson	Isturitz	52.939	16.7	3.1*	31370-32450 BP
Russia_Sunghir_UP	GoyetQ116	Isturitz	12.411	36.7	0.338	29729-31785 BP
Russia_Kostenki14_UP	GoyetQ116	Isturitz	99.924	41.3	2.4	32048-34366 BP
Czechia_Gravettian	GoyetQ116	Ormesson	40.053	26.3	1.5	31233-32705 BP
Russia_Sunghir_UP	GoyetQ116	Ormesson	34.918	17.0	2.0	31349-32301 BP
Russia_Kostenki14_UP	GoyetQ116	Ormesson	-	-	-	-
Czechia_Gravettian	GoyetQ116	Fournol	184.273	90.9	2.0	31351-36443 BP
Russia_Sunghir_UP	GoyetQ116	Fournol	213.879	119.3	1.7	31385-38066 BP
Russia_Kostenki14_UP	GoyetQ116	Fournol	338.614	340.7	0.9	28737-47758 BP

Table S1.

Summary of sequencing and mapping statistics for the Isturitz and Ostuni 1b samples

Table S2.

Summary of sequencing and mapping statistics for the Chufin sample

Table S3.

Summary of sequencing and mapping statistics for ElMiron14 sample

Table S4.

Alleles at relevant mtDNA positions in captured mtDNA from the ElMiron14 sample

Table S5.

Dataset used for population genetic comparisons, including sample information (version, group, population label, culture, metapopulation, location, country, publication, calibrated age), geographic coordinates, and the number of SNPs analyzed.

Table S6.

f4-statistic combinations used in the clustering analyses.

Table S7.

Complete values of the f4-statistics reported in the manuscript, including all tests used in the analyses.

Table S8.

Prediction of Runs of Homozygosity and effective population estimates in Aurignacian and Gravettian individuals

REFERENCES

1. J. Wilczyński, T. Goslar, P. Wojtal, M. Oliva, U. B. Göhlich, W. Antl-Weiser, P. Šída, A. Verpoorte, G. Lengyel, New radiocarbon dates for the Late Gravettian in eastern Central Europe. *Radiocarbon* **62**, 243–259 (2020).

2. J. K. Kozłowski, The origin of the gravettian. *Quat. Int.* **359-360**, 3–18 (2015).

3. J. Baker, S. Rigaud, D. Pereira, L. A. Courtenay, F. d'Errico, Evidence from personal ornaments suggest nine distinct cultural groups between 34,000 and 24,000 years ago in Europe. *Nat. Hum. Behav.* **8**, 431–444 (2024).

4. V. Borgia, Hunting high and low: Gravettian hunting weapons from Southern Italy to the Russian Plain. *Open Archaeol.* **3**, 376–391 (2017).

5. A. Mounier, Y. Heuzé, M. Samsel, S. Vasilyev, L. Klaric, S. Villotte, Gravettian cranial morphology and human group affinities during the European Upper Palaeolithic. *Sci. Rep.* **10**, 21931 (2020).

6. H. Rathmann, M. T. Vizzari, J. Beier, S. E. Bailey, S. Ghirotto, K. Harvati, Human population dynamics in Upper Paleolithic Europe inferred from fossil dental phenotypes. *Sci. Adv.* **10**, eadn8129 (2024).

7. Q. Fu, C. Posth, M. Hajdinjak, M. Petr, S. Mallick, D. Fernandes, A. Furtwängler, W. Haak, M. Meyer, A. Mittnik, B. Nickel, A. Peltzer, N. Rohland, V. Slon, S. Talamo, I. Lazaridis, M. Lipson, I. Mathieson, S. Schiffels, P. Skoglund, A. P. Derevianko, N. Drozdov, V. Slavinsky, A. Tsybankov, R. G. Cremonesi, F. Mallegni, B. Gély, E. Vacca, M. R. G. Morales, L. G. Straus, C. Neugebauer-Maresch, M. Teschler-Nicola, S. Constantin, O. T. Moldovan, S. Benazzi, M. Peresani, D. Coppola, M. Lari, S. Ricci, A. Ronchitelli, F. Valentin, C. Thevenet, K. Wehrberger, D. Grigorescu, H. Rougier, I. Crevecoeur, D. Flas, P. Semal, M. A. Mannino, C. Cupillard, H. Bocherens, N. J. Conard, K. Harvati, V. Moiseyev, D. G. Drucker, J. Svoboda, M. P. Richards, D. Caramelli, R. Pinhasi, J. Kelso, N. Patterson, J. Krause, S. Pääbo, D. Reich, The genetic history of Ice Age Europe. *Nature* **534**, 200–205 (2016).

8. C. Posth, H. Yu, A. Ghalichi, H. Rougier, I. Crevecoeur, Y. Huang, H. Ringbauer, A. B. Rohrlach, K. Nägele, V. Villalba-Mouco, R. Radzeviciute, T. Ferraz, A. Stoessel, R. Tukhbatova, D. G. Drucker, M. Lari, A. Modi, S. Vai, T. Saupe, C. L. Scheib, G. Catalano, L. Pagani, S. Talamo, H. Fewlass, L. Klaric, A. Morala, M. Rué, S. Madelaine, L. Crépin, J.-B. Caverne, E. Bocaege, S. Ricci, F. Boschin, P. Bayle, B. Maureille, F. Le Brun-Ricalens, J.-G. Bordes, G. Oxilia, E. Bortolini, O. Bignon-Lau, G. Debout, M. Orliac, A. Zazzo, V. Sparacello, E. Starnini, L. Sineo, J. van der Plicht, L. Pecqueur, G. Merceron, G. Garcia, J.-M. Leuvrey, C. B. Garcia, A. Gómez-Olivencia, M. Połtowicz-Bobak, D. Bobak, M. Le Luyer, P. Storm, C. Hoffmann, J. Kabaciński, T. Filimonova, S. Shnaider, N. Berezina, B. González-Rabanal, M. R. González Morales, A. B. Marín-Arroyo, B. López, C. Alonso-Llamazares, A. Ronchitelli, C. Polet, I. Jadin, N. Cauwe, J. Soler, N. Coromina, I. Rufí, R. Cottiaux, G. Clark, L. G. Straus, M.-A. Julien, S. Renhart, D. Talaa, S. Benazzi, M. Romandini, L. Amkreutz, H. Bocherens, C. Wißing, S. Villotte, J. F.-L. de Pablo, M. Gómez-Puche, M. A. Esquembre-Bebia, P. Bodu, L. Smits, B. Souffi, R. Jankauskas, J. Kozakaitė, C. Cupillard, H. Benthien, K. Wehrberger, R. W. Schmitz, S. C. Feine, T. Schüler, C. Thevenet, D. Grigorescu, F. Lüth, A. Kotula, H. Piezonka, F. Schopper, J. Svoboda, S. Sázelová, A. Chizhevsky, A. Khokhlov, N. J. Conard, F. Valentin, K. Harvati, P. Semal, B. Jungklaus, A. Suvorov, R. Schulting, V. Moiseyev, K. Mannermaa, A. Buzhilova, T. Terberger, D. Caramelli, E. Altena, W. Haak, J. Krause, Palaeogenomics of Upper Palaeolithic to Neolithic European hunter-gatherers. *Nature* **615**, 117–126 (2023).

9. M. Sikora, A. Seguin-Orlando, V. C. Sousa, A. Albrechtsen, T. Korneliussen, A. Ko, S. Rasmussen, I. Dupanloup, P. R. Nigst, M. D. Bosch, G. Renaud, M. E. Allentoft, A. Margaryan, S. V. Vasilyev, E. V. Veselovskaya, S. B. Borutskaya, T. Deviese, D. Comeskey, T. Higham, A. Manica, R. Foley, D. J. Meltzer, R. Nielsen, L. Excoffier, M. Mirazon Lahr, L. Orlando, E. Willerslev, Ancient genomes show social and reproductive behavior of early Upper Paleolithic foragers. *Science* **358**, 659–662 (2017).

10. A. Seguin-Orlando, T. S. Korneliussen, M. Sikora, A.-S. Malaspinas, A. Manica, I. Moltke, A. Albrechtsen, A. Ko, A. Margaryan, V. Moiseyev, T. Goebel, M. Westaway, D. Lambert, V. Khartanovich, J. D. Wall, P. R. Nigst, R. A. Foley, M. M. Lahr, R. Nielsen, L. Orlando, E. Willerslev, Paleogenomics. Genomic structure in Europeans dating back at least 36,200 years. *Science* **346**, 1113–1118 (2014).

11. M. Hajdinjak, F. Mafessoni, L. Skov, B. Vernot, A. Hübner, Q. Fu, E. Essel, S. Nagel, B. Nickel, J. Richter, O. T. Moldovan, S. Constantin, E. Endarova, N. Zahariev, R. Spasov, F. Welker, G. M. Smith, V. Sinet-Mathiot, L. Paskulin, H. Fewlass, S. Talamo, Z. Rezek, S. Sirakova, N. Sirakov, S. P. McPherron, T. Tsanova, J.-J. Hublin, B. M. Peter, M. Meyer, P. Skoglund, J. Kelso, S. Pääbo, Initial Upper Palaeolithic humans in Europe had recent Neanderthal ancestry. *Nature* **592**, 253–257 (2021).

12. J.-J. Hublin, N. Sirakov, V. Aldeias, S. Bailey, E. Bard, V. Delvigne, E. Endarova, Y. Fagault, H. Fewlass, M. Hajdinjak, B. Kromer, I. Krumov, J. Marreiros, N. L. Martisius, L. Paskulin, V. Sinet-Mathiot, M. Meyer, S. Pääbo, V. Popov, Z. Rezek, S. Sirakova, M. M. Skinner, G. M. Smith, R. Spasov, S. Talamo, T. Tuna, L. Wacker, F. Welker, A. Wilcke, N. Zahariev, S. P. McPherron, T. Tsanova, Initial Upper Palaeolithic *Homo sapiens* from Bacho Kiro Cave, Bulgaria. *Nature* **581**, 299–302 (2020).

13. V. Villalba-Mouco, M. S. van de Loosdrecht, A. B. Rohrlach, H. Fewlass, S. Talamo, H. Yu, F. Aron, C. Lalueza-Fox, L. Cabello, P. Cantalejo Duarte, J. Ramos-Muñoz, C. Posth, J. Krause, G.-C. Weniger, W. Haak, A 23,000-year-old southern Iberian individual links human groups that lived in Western Europe before and after the Last Glacial Maximum. *Nat. Ecol. Evol.* **7**, 597–609 (2023).

14. V. Cabrera, F. B. de Quiros, The Solutrean Site of Cueva Chufin (Santander, Spain). *Curr. Anthropol.* **18**, 780–781 (1977).

15. L. G. Straus, The human occupation of Southwestern Europe during the Last Glacial Maximum. *J. Anthropol. Res.* **71**, 465–492 (2015).

16. N. Naudinot, A. Tomasso, C. Tozzi, M. Peresani, Changes in mobility patterns as a factor of 14C date density variation in the Late Epigravettian of Northern Italy and Southeastern France. *J. Archaeol. Sci.* **52**, 578–590 (2014).

17. O. E. Yavuz, G. Oxilia, S. Silvestrini, L. Tassoni, E. Reiter, D. G. Drucker, S. Talamo, F. Fontana, S. Benazzi, C. Posth, Biomolecular analysis of the Epigravettian human remains from Riparo Tagliente in northern Italy. *Commun. Biol.* **7**, 1415 (2024).

18. P. Gelabert, S. Sawyer, A. Bergström, A. Margaryan, T. C. Collin, T. Meshveliani, A. Belfer-Cohen, D. Lordkipanidze, N. Jakeli, Z. Matskevich, G. Bar-Oz, D. M. Fernandes, O. Cheronet, K. T. Özdoğan, V. Oberreiter, R. N. M. Feeney, M. C. Stahlschmidt, P. Skoglund, R. Pinhasi, Genome-scale sequencing and analysis of human, wolf, and bison DNA from 25,000-year-old sediment. *Curr. Biol.* **31**, 3564–3574.e9 (2021).

19. B. Vernot, E. I. Zavala, A. Gómez-Olivencia, Z. Jacobs, V. Slon, F. Mafessoni, F. Romagné, A. Pearson, M. Petr, N. Sala, A. Pablos, A. Aranburu, J. M. B. de Castro, E. Carbonell, B. Li, M. T. Krajcarz, A. I. Krivoshapkin, K. A. Kolobova, M. B. Kozlikin, M. V. Shunkov, A. P. Derevianko, B. Viola, S. Grote, E. Essel, D. L. Herráez, S. Nagel, B. Nickel, J. Richter, A. Schmidt, B. Peter, J. Kelso, R. G. Roberts, J.-L. Arsuaga, M. Meyer, Unearthing Neanderthal population history using nuclear and mitochondrial DNA from cave sediments. *Science* **372**, eabf1667 (2021).

20. Z. Jacobs, E. I. Zavala, B. Li, K. O'Gorman, M. V. Shunkov, M. B. Kozlikin, A. P. Derevianko, V. A. Uliyanov, P. Goldberg, A. K. Agadjanian, S. K. Vasiliev, F. Brink, S. Peyrégne, V. Slon, S. Pääbo, J. Kelso, M. Meyer, R. G. Roberts, Pleistocene chronology and history of hominins and fauna at Denisova Cave. *Nat. Commun.* **16**, 4738 (2025).

21. V. Aldeias, M. C. Stahlschmidt, Sediment DNA can revolutionize archaeology-if it is used the right way. *Proc. Natl. Acad. Sci. U.S.A.* **121**, e2317042121 (2024).

22. P. Gelabert, V. Oberreiter, L. G. Straus, M. R. G. Morales, S. Sawyer, A. B. Marín-Arroyo, J. Geiling, F. Exler, F. Brueck, S. Franz, F. T. Cano, S. Szedlacsek, E. Zelger, M. Hämmerle, B. Zagorc, A. Llanos-Lizcano, O. Cheronet, J.-M. Tejero, T. Rattei, S. M. Kraemer, R. Pinhasi, A sedimentary ancient DNA perspective on human and carnivore persistence through the Late Pleistocene in El Mirón Cave, Spain. *Nat. Commun.* **16**, 107 (2025).

23. E. I. Zavala, Z. Jacobs, B. Vernot, M. V. Shunkov, M. B. Kozlikin, A. P. Derevianko, E. Essel, C. de Fillipo, S. Nagel, J. Richter, F. Romagné, A. Schmidt, B. Li, K. O'Gorman, V. Slon, J. Kelso, S. Pääbo, R. G. Roberts, M. Meyer, Pleistocene sediment DNA reveals hominin and faunal turnovers at Denisova Cave. *Nature* **595**, 399–403 (2021).

24. M.-C. Soulier, "Entre alimentaire et technique: l'exploitation animale aux débuts du paléolithique supérieur: stratégies de subsistance et chaînes opératoires de traitement du gibier à Isturitz, La Quina aval, Roc-de-Combe et Les Abeilles," thesis, Université Toulouse le Mirail (2013).

25. C. Normand, S. A. de Beaune, S. Costamagno, M.-F. Diot, D. Henry-Gambier, N. Goutas, V. Laroulandie, A. Lenoble, M. O'Farrell, W. Rendu, J. R. Garaizar, C. Schwab, A. T. Vinagre, P.-J. Texier, R. White, "Nouvelles données sur la séquence aurignacienne de la grotte d'Isturitz (communes d'Isturitz et de Saint-Martin-d'Arberoue. Pyrénées-Atlantiques)," *Un Siècle de Construction du Discours Scientifique en Préhistoire, vol. III "…Aux Conceptions d'Aujourd'hui," Actes du Congrès Préhistorique de France, XXVIe Session, Congrès du Centenaire, 21-25 Septembre 2004, Avignon* (Mémoires de la Société Préhistorique Française, 2007), pp. 277–293.

26. J.-M. Pétillon, P. Cattelain, Experimental evidence of spear-thrower usage in the late upper Palaeolithic (Upper Magdalenian) from the Isturitz cave site, Pyrénées-atlantiques, France. *Archaeology* **2**, 5–18 (2022).

27. M. Almagro Gorbea, V. Cabrera Valdés, F. B. de Quirós Guidotti, Nuevos hallazgos de arte rupestre en Cueva Chufin: Riclones (Santander). *Trabajos De Prehistoria* **34**, 9–30 (1977).

28. E. Vacca, D. Coppola, The Upper Palaeolithic burial at the cave of Santa Maria di Agnano (Ostuni, Brindisi): Preliminary report. *Rivista di Antropologia* **71**, 275–284 (1993).

29. N. Rohland, S. Mallick, M. Mah, R. Maier, N. Patterson, D. Reich, Three assays for in-solution enrichment of ancient human DNA at more than a million SNPs. *Genome Res.* **32**, 2068–2078 (2022).

30. E. Alaçamlı, T. Naidoo, M. N. Güler, E. Sağlıcan, Ş. Aktürk, I. Mapelli, K. B. Vural, M. Somel, H. Malmström, T. Günther, READv2: Advanced and user-friendly detection of biological relatedness in archaeogenomics. *Genome Biol.* **25**, 216 (2024).

31. R. J. A. Hopkins, L. G. Straus, M. R. González Morales, Assessing the chronostratigraphy of el Mirón cave, Cantabrian Spain. *Radiocarbon* **63**, 821–852 (2021).

32. L. G. Straus, El Solutrense: 40 Anos de Reflexiones por un Arqueologo Norteamericano. *ETFI* **1**, 25–34 (2013).

33. S. Monterrosa, C. Real, A. Sanchis, V. Villaverde, Solutrean macrofauna from Cova de les Cendres (Alicante, Spain): Zooarchaeological and taphonomic analysis. *Archaeofauna* **32**, 10.15366/archaeofauna2023.32.1.003 (2023).

34. L. G. Straus, Pointes solutréennes et l'hypothèse de territorialisme. *Bull. Soc. Préhist. Fr.* **74**, 206–212 (1977).

35. S. Hinguant, P.-E. Moullé, A. Arellano, R. Pigeaud, Pièces osseuses gravées du Solutréen moyen de la grotte Rochefort (Saint-Pierre-sur-Erve, Mayenne, France). *Paléo* **23**, 337–356 (2012).

36. A. Mittnik, C.-C. Wang, S. Pfrengle, M. Daubaras, G. Zariņa, F. Hallgren, R. Allmäe, V. Khartanovich, V. Moiseyev, M. Tõrv, A. Furtwängler, A. Andrades Valtueña, M. Feldman, C. Economou, M. Oinonen, A. Vasks, E. Balanovska, D. Reich, R. Jankauskas, W. Haak, S. Schiffels, J. Krause, The genetic prehistory of the Baltic Sea region. *Nat. Commun.* **9**, 442 (2018).

37. A. M. González, O. García, J. M. Larruga, V. M. Cabrera, The mitochondrial lineage U8a reveals a Paleolithic settlement in the Basque country. *BMC Genomics* **7**, 124 (2006).

38. C. Posth, G. Renaud, A. Mittnik, D. G. Drucker, H. Rougier, C. Cupillard, F. Valentin, C. Thevenet, A. Furtwängler, C. Wißing, M. Francken, M. Malina, M. Bolus, M. Lari, E. Gigli, G. Capecchi, I. Crevecoeur, C. Beauval, D. Flas, M. Germonpré, J. van der Plicht, R. Cottiaux, B. Gély, A. Ronchitelli, K. Wehrberger, D. Grigorescu, J. Svoboda, P. Semal, D. Caramelli, H. Bocherens, K. Harvati, N. J. Conard, W. Haak, A. Powell, J. Krause, Pleistocene mitochondrial genomes suggest a single major dispersal of Non-Africans and a Late Glacial population turnover in Europe. *Curr. Biol.* **26**, 827–833 (2016).

39. N. Patterson, P. Moorjani, Y. Luo, S. Mallick, N. Rohland, Y. Zhan, T. Genschoreck, T. Webster, D. Reich, Ancient admixture in human history. *Genetics* **192**, 1065–1093 (2012).

40. R. Maier, P. Flegontov, O. Flegontova, U. Işıldak, P. Changmai, D. Reich, On the limits of fitting complex models of population history to *f*-statistics. *eLife* **12**, e85492 (2023).

41. Z. Maróti, E. Nyerki, T. Török, G. I. Varga, T. Kalmár, Robust imputation-based method for eye, hair, and skin colour prediction from low-coverage ancient DNA. *Sci. Rep.* **16**, 7371 (2026).

42. J. Lacarrière, P. Bodu, M.-A. Julien, G. Dumarçay, N. Goutas, M. Lejay, C. Peschaux, H.-G. Naton, I. Théry-Parisot, L. Vasiliu, Les Bossats (Ormesson, Paris basin, France): A new early Gravettian bison processing camp. *Quat. Int.* **359-360**, 520–534 (2015).

43. A. B. Marín-Arroyo, J. Rios-Garaizar, L. G. Straus, J. R. Jones, M. de la Rasilla, M. R. González Morales, M. Richards, J. Altuna, K. Mariezkurrena, D. Ocio, Chronological reassessment of the Middle to Upper Paleolithic transition and Early Upper Paleolithic cultures in Cantabrian Spain. *PLOS ONE* **13**, e0194708 (2018).

44. C. Barshay-Szmidt, C. Normand, D. Flas, M.-C. Soulier, Radiocarbon dating the Aurignacian sequence at Isturitz (France): Implications for the timing and development of the Protoaurignacian and Early Aurignacian in Western Europe. *J. Archaeol. Sci. Rep.* **17**, 809–838 (2018).

45. K. McGrath, L. G. van der Sluis, A. Lefebvre, A. Charpentier, A. S. L. Rodrigues, E. Álvarez-Fernández, F. Baleux, E. Berganza, F.-X. Chauvière, M. Dachary, E. Duarte Matías, C. Houmard, A. B. Marín-Arroyo, M. de la Rasilla Vives, J. Tapia, F. Thil, O. Tombret, L. Torres-Iglesias, C. Speller, A. Zazzo, J.-M. Pétillon, Late Paleolithic whale bone tools reveal human and whale ecology in the Bay of Biscay. *Nat. Commun.* **16**, 4646 (2025).

46. P. G. Bahn, Inter-site and inter-regional links during the Upper Palaeolithic: The Pyrenean evidence. *Oxf. J. Archaeol.* **1**, 247–268 (1982).

47. L. Straus, Late Würm adaptive systems in Cantabrian Spain: The case of Eastern Asturias. *J. Anthropol. Archaeol.* **5**, 330–368 (1986).

48. M. W. Conkey, A. Beltrán, G. A. Clark, J. G. Echegaray, M. G. Guenther, J. Hahn, B. Hayden, K. Paddayya, L. G. Straus, K. Valoch, The identification of prehistoric Hunter-gatherer aggregation sites: The case of Altamira [and comments and reply]. *Curr. Anthropol.* **21**, 609–630 (1980).

49. J. Dabney, M. Meyer, "Extraction of highly degraded DNA from ancient bones and teeth," in *Ancient DNA: Methods and Protocols*, B. Shapiro, A. Barlow, P. D. Heintzman, M. Hofreiter, J. L. A. Paijmans, A. E. R. Soares, Eds. (Springer, 2019), pp. 25–29.

50. P. Korlević, T. Gerber, M.-T. Gansauge, M. Hajdinjak, S. Nagel, A. Aximu-Petri, M. Meyer, Reducing microbial and human contamination in DNA extractions from ancient bones and teeth. *Biotechniques* **59**, 87–93 (2015).

51. M. Meyer, M. Kircher, Illumina sequencing library preparation for highly multiplexed target capture and sequencing. *Cold Spring Harb. Protoc.* **2010**, pdb.prot5448 (2010).

52. M. Kircher, S. Sawyer, M. Meyer, Double indexing overcomes inaccuracies in multiplex sequencing on the Illumina platform. *Nucleic Acids Res.* **40**, e3 (2012).

53. M. Martin, Cutadapt removes adapter sequences from high-throughput sequencing reads. *EMBnet J.* **17**, 10–12 (2011).

54. S. Chen, Y. Zhou, Y. Chen, J. Gu, fastp: An ultra-fast all-in-one FASTQ preprocessor. *Bioinformatics* **34**, i884–i890 (2018).

55. H. Li, R. Durbin, Fast and accurate short read alignment with Burrows-Wheeler transform. *Bioinformatics* **25**, 1754–1760 (2009).

56. Picard Tools; http://broadinstitute.github.io/picard.

57. K. Okonechnikov, A. Conesa, F. García-Alcalde, Qualimap 2: Advanced multi-sample quality control for high-throughput sequencing data. *Bioinformatics* **32**, 292–294 (2016).

58. H. Jónsson, A. Ginolhac, M. Schubert, P. L. F. Johnson, L. Orlando, mapDamage2.0: Fast approximate Bayesian estimates of ancient DNA damage parameters. *Bioinformatics* **29**, 1682–1684 (2013).

59. H. Li, B. Handsaker, A. Wysoker, T. Fennell, J. Ruan, N. Homer, G. Marth, G. Abecasis, R. Durbin, 1000 Genome Project Data Processing Subgroup, The Sequence Alignment/Map format and SAMtools. *Bioinformatics* **25**, 2078–2079 (2009).

60. S. F. Altschul, W. Gish, W. Miller, E. W. Myers, D. J. Lipman, Basic local alignment search tool. *J. Mol. Biol.* **215**, 403–410 (1990).

61. D. H. Huson, A. F. Auch, J. Qi, S. C. Schuster, MEGAN analysis of metagenomic data. *Genome Res.* **17**, 377–386 (2007).

62. J. Zhang, K. Kobert, T. Flouri, A. Stamatakis, PEAR: A fast and accurate Illumina Paired-End reAd mergeR. *Bioinformatics* **30**, 614–620 (2014).

63. G. Renaud, V. Slon, A. T. Duggan, J. Kelso, Schmutzi: Estimation of contamination and endogenous mitochondrial consensus calling for ancient DNA. *Genome Biol.* **16**, 224 (2015).

64. S. Schönherr, H. Weissensteiner, F. Kronenberg, L. Forer, Haplogrep 3 - an interactive haplogroup classification and analysis platform. *Nucleic Acids Res.* **51**, W263–W268 (2023).

65. M. Szymanski, J. Visagie, F. Romagné, M. Meyer, J. Kelso, Quick analysis of sedimentary ancient DNA using quicksand. *Mol. Biol. Evol.* **42**, msaf305 (2025).

66. J. Krause, Q. Fu, J. M. Good, B. Viola, M. V. Shunkov, A. P. Derevianko, S. Pääbo, The complete mitochondrial DNA genome of an unknown hominin from southern Siberia. *Nature* **464**, 894–897 (2010).

67. T. S. Korneliussen, A. Albrechtsen, R. Nielsen, ANGSD: Analysis of next generation sequencing data. *BMC Bioinformatics* **15**, 356 (2014).

68. P. Skoglund, B. H. Northoff, M. V. Shunkov, A. P. Derevianko, S. Pääbo, J. Krause, M. Jakobsson, Separating endogenous ancient DNA from modern day contamination in a Siberian Neandertal. *Proc. Natl. Acad. Sci. U.S.A.* **111**, 2229–2234 (2014).

69. A. McKenna, M. Hanna, E. Banks, A. Sivachenko, K. Cibulskis, A. Kernytsky, K. Garimella, D. Altshuler, S. Gabriel, M. Daly, M. A. DePristo, The genome analysis toolkit: A MapReduce framework for analyzing next-generation DNA sequencing data. *Genome Res.* **20**, 1297–1303 (2010).

70. bamUtil, Github; https://github.com/statgen/bamUtil.

71. S. Schiffels, W. Haak, P. Paajanen, B. Llamas, E. Popescu, L. Loe, R. Clarke, A. Lyons, R. Mortimer, D. Sayer, C. Tyler-Smith, A. Cooper, R. Durbin, Iron age and Anglo-Saxon genomes from East England reveal British migration history. *Nat. Commun.* **7**, 10408 (2016).

72. S. Purcell, B. Neale, K. Todd-Brown, L. Thomas, M. A. R. Ferreira, D. Bender, J. Maller, P. Sklar, P. I. W. de Bakker, M. J. Daly, P. C. Sham, PLINK: A tool set for whole-genome association and population-based linkage analyses. *Am. J. Hum. Genet.* **81**, 559–575 (2007).

73. A. Ralf, D. Montiel González, K. Zhong, M. Kayser, Yleaf: Software for human Y-chromosomal haplogroup inference from next-generation sequencing data. *Mol. Biol. Evol.* **35**, 1291–1294 (2018).

74. S. Mallick, A. Micco, M. Mah, H. Ringbauer, I. Lazaridis, I. Olalde, N. Patterson, D. Reich, The Allen Ancient DNA Resource (AADR) a curated compendium of ancient human genomes. *Sci. Data* **11**, 182 (2024).

75. R Core Team, R: A language and environment for statistical computing (R Foundation for Statistical Computing, 2013); www.R-project.org/.

76. M. Chintalapati, N. Patterson, P. Moorjani, The spatiotemporal patterns of major human admixture events during the European Holocene. *eLife* **11**, e77625 (2022).

77. H. Ringbauer, J. Novembre, M. Steinrücken, Parental relatedness through time revealed by runs of homozygosity in ancient DNA. *Nat. Commun.* **12**, 5425 (2021).

78. T. F. G. Higham, R. M. Jacobi, C. B. Ramsey, AMS radiocarbon dating of ancient bone using ultrafiltration. *Radiocarbon* **48**, 179–195 (2006).

79. F. Brock, T. Higham, P. Ditchfield, C. B. Ramsey, Current pretreatment methods for AMS radiocarbon dating at the Oxford Radiocarbon Accelerator Unit (ORAU). *Radiocarbon* **52**, 103–112 (2010).

80. G. Jun, M. K. Wing, G. R. Abecasis, H. M. Kang, An efficient and scalable analysis framework for variant extraction and refinement from population-scale DNA sequence data. *Genome Res.* **25**, 918–925 (2015).

81. C. Normand, "La grotte d'Isturitz: Histoire de la recherche et des occupations humaines," in *L'art Pariétal Des Grottes de La Colline de Gazteul (Pays Basque)*, vol. 2 of *Anthropology and Archaeology Series*, D. Garate Maidagan, O. Rivero Vilá, Eds. (Munibe Monographs, 2024), pp. 61–81.

82. K. Prüfer, C. Posth, H. Yu, A. Stoessel, M. A. Spyrou, T. Deviese, M. Mattonai, E. Ribechini, T. Higham, P. Velemínský, J. Brůžek, J. Krause, A genome sequence from a modern human skull over 45,000 years old from Zlatý kůň in Czechia. *Nat. Ecol. Evol.* **5**, 820–825 (2021).

83. H. Ringbauer, J. Novembre, M. Steinrücken, Detecting runs of homozygosity from low-coverage ancient DNA. bioRxiv 126912 [Preprint] (2020). https://doi.org/10.1101/2020.05.31.126912.

84. E. Vacca, V. Formicola, V. Pesce Delfino, D. Coppola, "I resti scheletrici umani delle sepolture paleolitiche di Grotta Santa Maria d'Agnano-Ostuni (BR)," in *Il Riparo di Agnano nel Paleolitico Superiore* (Università di Roma Tor Vergata, 2012), pp. 205–361.

85. A. Nava, A. Coppa, D. Coppola, L. Mancini, D. Dreossi, F. Zanini, F. Bernardini, C. Tuniz, L. Bondioli, Virtual histological assessment of the prenatal life history and age at death of the Upper Paleolithic fetus from Ostuni (Italy). *Sci. Rep.* **7**, 9427 (2017).

86. R. Pinhasi, D. M. Fernandes, K. Sirak, O. Cheronet, Isolating the human cochlea to generate bone powder for ancient DNA analysis. *Nat. Protoc.* **14**, 1194–1205 (2019).

87. N. Rohland, E. Harney, S. Mallick, S. Nordenfelt, D. Reich, Partial uracil–DNA–glycosylase treatment for screening of ancient DNA. *Philos. Trans. R. Soc. B* **370**, 20130624 (2015).

88. L. G. Straus, M. R. G. Morales, A window into 40,000 years of the prehistory of Iberia: The long excavation of El mirón cave, Cantabrian Spain. *J. Anthropol. Res.* **82**, 1–42 (2026).

89. I. Mathieson, S. Alpaslan-Roodenberg, C. Posth, A. Szécsényi-Nagy, N. Rohland, S. Mallick, I. Olalde, N. Broomandkhoshbacht, F. Candilio, O. Cheronet, D. Fernandes, M. Ferry, B. Gamarra, G. G. Fortes, W. Haak, E. Harney, E. Jones, D. Keating, B. Krause-Kyora, I. Kucukkalipci, M. Michel, A. Mittnik, K. Nägele, M. Novak, J. Oppenheimer, N. Patterson, S. Pfrengle, K. Sirak, K. Stewardson, S. Vai, S. Alexandrov, K. W. Alt, R. Andreescu, D. Antonović, A. Ash, N. Atanassova, K. Bacvarov, M. B. Gusztáv, H. Bocherens, M. Bolus, A. Boroneanţ, Y. Boyadzhiev, A. Budnik, J. Burmaz, S. Chohadzhiev, N. J. Conard, R. Cottiaux, M. Čuka, C. Cupillard, D. G. Drucker, N. Elenski, M. Francken, B. Galabova, G. Ganetsovski, B. Gély, T. Hajdu, V. Handzhyiska, K. Harvati, T. Higham, S. Iliev, I. Janković, I. Karavanić, D. J. Kennett, D. Komšo, A. Kozak, D. Labuda, M. Lari, C. Lazar, M. Leppek, K. Leshtakov, D. L. Vetro, D. Los, I. Lozanov, M. Malina, F. Martini, K. McSweeney, H. Meller, M. Menđušić, P. Mirea, V. Moiseyev, V. Petrova, T. D. Price, A. Simalcsik, L. Sineo, M. Šlaus, V. Slavchev, P. Stanev, A. Starović, T. Szeniczey, S. Talamo, M. Teschler-Nicola, C. Thevenet, I. Valchev, F. Valentin, S. Vasilyev, F. Veljanovska, S. Venelinova, E. Veselovskaya, B. Viola, C. Virag, J. Zaninović, S. Zäuner, P. W. Stockhammer, G. Catalano, R. Krauß, D. Caramelli, G. Zariņa, B. Gaydarska, M. Lillie, A. G. Nikitin, I. Potekhina, A. Papathanasiou, D. Borić, C. Bonsall, J. Krause, R. Pinhasi, D. Reich, The genomic history of southeastern Europe. *Nature* **555**, 197–203 (2018).

90. D. Reich, R. E. Green, M. Kircher, J. Krause, N. Patterson, E. Y. Durand, B. Viola, A. W. Briggs, U. Stenzel, P. L. F. Johnson, T. Maricic, J. M. Good, T. Marques-Bonet, C. Alkan, Q. Fu, S. Mallick, H. Li, M. Meyer, E. E. Eichler, M. Stoneking, M. Richards, S. Talamo, M. V. Shunkov, A. P. Derevianko, J.-J. Hublin, J. Kelso, M. Slatkin, S. Pääbo, Genetic history of an archaic hominin group from Denisova Cave in Siberia. *Nature* **468**, 1053–1060 (2010).

91. I. Mathieson, I. Lazaridis, N. Rohland, S. Mallick, N. Patterson, S. A. Roodenberg, E. Harney, K. Stewardson, D. Fernandes, M. Novak, K. Sirak, C. Gamba, E. R. Jones, B. Llamas, S. Dryomov, J. Pickrell, J. L. Arsuaga, J. M. B. de Castro, E. Carbonell, F. Gerritsen, A. Khokhlov, P. Kuznetsov, M. Lozano, H. Meller, O. Mochalov, V. Moiseyev, M. A. R. Guerra, J. Roodenberg, J. M. Vergès, J. Krause, A. Cooper, K. W. Alt, D. Brown, D. Anthony, C. Lalueza-Fox, W. Haak, R. Pinhasi, D. Reich, Genome-wide patterns of selection in 230 ancient Eurasians. *Nature* **528**, 499–503 (2015).

92. M. Rivollat, C. Jeong, S. Schiffels, İ. Küçükkalıpçı, M.-H. Pemonge, A. B. Rohrlach, K. W. Alt, D. Binder, S. Friederich, E. Ghesquière, D. Gronenborn, L. Laporte, P. Lefranc, H. Meller, H. Réveillas, E. Rosenstock, S. Rottier, C. Scarre, L. Soler, J. Wahl, J. Krause, M.-F. Deguilloux, W. Haak, Ancient genome-wide DNA from France highlights the complexity of interactions between Mesolithic hunter-gatherers and Neolithic farmers. *Sci. Adv.* **6**, eaaz5344 (2020).

93. K. Prüfer, F. Racimo, N. Patterson, F. Jay, S. Sankararaman, S. Sawyer, A. Heinze, G. Renaud, P. H. Sudmant, C. de Filippo, H. Li, S. Mallick, M. Dannemann, Q. Fu, M. Kircher, M. Kuhlwilm, M. Lachmann, M. Meyer, M. Ongyerth, M. Siebauer, C. Theunert, A. Tandon, P. Moorjani, J. Pickrell, J. C. Mullikin, S. H. Vohr, R. E. Green, I. Hellmann, P. L. F. Johnson, H. Blanche, H. Cann, J. O. Kitzman, J. Shendure, E. E. Eichler, E. S. Lein, T. E. Bakken, L. V. Golovanova, V. B. Doronichev, M. V. Shunkov, A. P. Derevianko, B. Viola, M. Slatkin, D. Reich, J. Kelso, S. Pääbo, The complete genome sequence of a Neanderthal from the Altai Mountains. *Nature* **505**, 43–49 (2014).

94. E. R. Jones, G. Gonzalez-Fortes, S. Connell, V. Siska, A. Eriksson, R. Martiniano, R. L. McLaughlin, M. Gallego Llorente, L. M. Cassidy, C. Gamba, T. Meshveliani, O. Bar-Yosef, W. Müller, A. Belfer-Cohen, Z. Matskevich, N. Jakeli, T. F. G. Higham, M. Currat, D. Lordkipanidze, M. Hofreiter, A. Manica, R. Pinhasi, D. G. Bradley, Upper Palaeolithic genomes reveal deep roots of modern Eurasians. *Nat. Commun.* **6**, 8912 (2015).

95. S. Brace, Y. Diekmann, T. J. Booth, L. van Dorp, Z. Faltyskova, N. Rohland, S. Mallick, I. Olalde, M. Ferry, M. Michel, J. Oppenheimer, N. Broomandkhoshbacht, K. Stewardson, R. Martiniano, S. Walsh, M. Kayser, S. Charlton, G. Hellenthal, I. Armit, R. Schulting, O. E.

Craig, A. Sheridan, M. Parker Pearson, C. Stringer, D. Reich, M. G. Thomas, I. Barnes, Ancient genomes indicate population replacement in Early Neolithic Britain. *Nat. Ecol. Evol.* **3**, 765–771 (2019).

96. I. Olalde, S. Mallick, N. Patterson, N. Rohland, V. Villalba-Mouco, M. Silva, K. Dulias, C. J. Edwards, F. Gandini, M. Pala, P. Soares, M. Ferrando-Bernal, N. Adamski, N. Broomandkhoshbacht, O. Cheronet, B. J. Culleton, D. Fernandes, A. M. Lawson, M. Mah, J. Oppenheimer, K. Stewardson, Z. Zhang, J. M. Jiménez Arenas, I. J. Toro Moyano, D. C. Salazar-García, P. Castanyer, M. Santos, J. Tremoleda, M. Lozano, P. García Borja, J. Fernández-Eraso, J. A. Mujika-Alustiza, C. Barroso, F. J. Bermúdez, E. Viguera Mínguez, J. Burch, N. Coromina, D. Vivó, A. Cebrià, J. M. Fullola, O. García-Puchol, J. I. Morales, F. X. Oms, T. Majó, J. M. Vergès, A. Díaz-Carvajal, I. Ollich-Castanyer, F. J. López-Cachero, A. M. Silva, C. Alonso-Fernández, G. Delibes de Castro, J. Jiménez Echevarría, A. Moreno-Márquez, G. Pascual Berlanga, P. Ramos-García, J. Ramos-Muñoz, E. Vijande Vila, G. Aguilella Arzo, Á. Esparza Arroyo, K. T. Lillios, J. Mack, J. Velasco-Vázquez, A. Waterman, L. B. de Lugo Enrich, M. B. Sánchez, B. Agustí, F. Codina, G. de Prado, A. Estalrrich, Á. Fernández Flores, C. Finlayson, G. Finlayson, S. Finlayson, F. Giles-Guzmán, A. Rosas, V. Barciela González, G. García Atiénzar, M. S. Hernández Pérez, A. Llanos, Y. Carrión Marco, I. Collado Beneyto, D. López-Serrano, M. Sanz Tormo, A. C. Valera, C. Blasco, C. Liesau, P. Ríos, J. Daura, M. J. de Pedro Michó, A. A. Diez-Castillo, R. Flores Fernández, J. Francès Farré, R. Garrido-Pena, V. S. Gonçalves, E. Guerra-Doce, A. M. Herrero-Corral, J. Juan-Cabanilles, D. López-Reyes, S. B. McClure, M. M. Pérez, A. O. Foix, M. Sanz Borràs, A. C. Sousa, J. M. V. Encinas, D. J. Kennett, M. B. Richards, K. W. Alt, W. Haak, R. Pinhasi, C. Lalueza-Fox, D. Reich, The genomic history of the Iberian Peninsula over the past 8000 years. *Science* **363**, 1230–1234 (2019).

97. I. Lazaridis, N. Patterson, A. Mittnik, G. Renaud, S. Mallick, K. Kirsanow, P. H. Sudmant, J. G. Schraiber, S. Castellano, M. Lipson, B. Berger, C. Economou, R. Bollongino, Q. Fu, K. I. Bos, S. Nordenfelt, H. Li, C. de Filippo, K. Prüfer, S. Sawyer, C. Posth, W. Haak, F. Hallgren, E. Fornander, N. Rohland, D. Delsate, M. Francken, J.-M. Guinet, J. Wahl, G. Ayodo, H. A. Babiker, G. Bailliet, E. Balanovska, O. Balanovsky, R. Barrantes, G. Bedoya, H. Ben-Ami, J. Bene, F. Berrada, C. M. Bravi, F. Brisighelli, G. B. J. Busby, F. Cali, M. Churnosov, D. E. C. Cole, D. Corach, L. Damba, G. van Driem, S. Dryomov, J.-M.

Dugoujon, S. A. Fedorova, I. Gallego Romero, M. Gubina, M. Hammer, B. M. Henn, T. Hervig, U. Hodoglugil, A. R. Jha, S. Karachanak-Yankova, R. Khusainova, E. Khusnutdinova, R. Kittles, T. Kivisild, W. Klitz, V. Kučinskas, A. Kushniarevich, L. Laredj, S. Litvinov, T. Loukidis, R. W. Mahley, B. Melegh, E. Metspalu, J. Molina, J. Mountain, K. Näkkäläjärvi, D. Nesheva, T. Nyambo, L. Osipova, J. Parik, F. Platonov, O. Posukh, V. Romano, F. Rothhammer, I. Rudan, R. Ruizbakiev, H. Sahakyan, A. Sajantila, A. Salas, E. B. Starikovskaya, A. Tarekegn, D. Toncheva, S. Turdikulova, I. Uktveryte, O. Utevska, R. Vasquez, M. Villena, M. Voevoda, C. A. Winkler, L. Yepiskoposyan, P. Zalloua, T. Zemunik, A. Cooper, C. Capelli, M. G. Thomas, A. Ruiz-Linares, S. A. Tishkoff, L. Singh, K. Thangaraj, R. Villems, D. Comas, R. Sukernik, M. Metspalu, M. Meyer, E. E. Eichler, J. Burger, M. Slatkin, S. Pääbo, J. Kelso, D. Reich, J. Krause, Ancient human genomes suggest three ancestral populations for present-day Europeans. *Nature* **513**, 409–413 (2014).

98. S. Brunel, E. A. Bennett, L. Cardin, D. Garraud, H. Barrand Emam, A. Beylier, B. Boulestin, F. Chenal, E. Ciesielski, F. Convertini, B. Dedet, S. Desbrosse-Degobertiere, S. Desenne, J. Dubouloz, H. Duday, G. Escalon, V. Fabre, E. Gailledrat, M. Gandelin, Y. Gleize, S. Goepfert, J. Guilaine, L. Hachem, M. Ilett, F. Lambach, F. Maziere, B. Perrin, S. Plouin, E. Pinard, I. Praud, I. Richard, V. Riquier, R. Roure, B. Sendra, C. Thevenet, S. Thiol, E. Vauquelin, L. Vergnaud, T. Grange, E.-M. Geigl, M. Pruvost, Ancient genomes from present-day France unveil 7,000 years of its demographic history. *Proc. Natl. Acad. Sci. U.S.A.* **117**, 12791–12798 (2020).

99. M. L. Antonio, Z. Gao, H. M. Moots, M. Lucci, F. Candilio, S. Sawyer, V. Oberreiter, D. Calderon, K. Devitofranceschi, R. C. Aikens, S. Aneli, F. Bartoli, A. Bedini, O. Cheronet, D. J. Cotter, D. M. Fernandes, G. Gasperetti, R. Grifoni, A. Guidi, F. La Pastina, E. Loreti, D. Manacorda, G. Matullo, S. Morretta, A. Nava, V. Fiocchi Nicolai, F. Nomi, C. Pavolini, M. Pentiricci, P. Pergola, M. Piranomonte, R. Schmidt, G. Spinola, A. Sperduti, M. Rubini, L. Bondioli, A. Coppa, R. Pinhasi, J. K. Pritchard, Ancient Rome: A genetic crossroads of Europe and the Mediterranean. *Science* **366**, 708–714 (2019).

100. L. M. Cassidy, R. Ó. Maoldúin, T. Kador, A. Lynch, C. Jones, P. C. Woodman, E. Murphy, G. Ramsey, M. Dowd, A. Noonan, C. Campbell, E. R. Jones, V. Mattiangeli, D. G. Bradley, A dynastic elite in monumental Neolithic society. *Nature* **582**, 384–388 (2020).

101. M. A. Yang, X. Gao, C. Theunert, H. Tong, A. Aximu-Petri, B. Nickel, M. Slatkin, M. Meyer, S. Pääbo, J. Kelso, Q. Fu, 40,000-Year-old individual from Asia provides insight into early population structure in Eurasia. *Curr. Biol.* **27**, 3202–3208.e9 (2017).

102. Q. Fu, M. Hajdinjak, O. T. Moldovan, S. Constantin, S. Mallick, P. Skoglund, N. Patterson, N. Rohland, I. Lazaridis, B. Nickel, B. Viola, K. Prüfer, M. Meyer, J. Kelso, D. Reich, S. Pääbo, An early modern human from Romania with a recent Neanderthal ancestor. *Nature* **524**, 216–219 (2015).
